# Synthesis and Biological Evaluation of Seco-Coumarin/Furoxan Hybrids as Potent Anti-Tumor Agents to Overcome Multidrug Resistance via Multiple Mechanisms

**DOI:** 10.3390/molecules30112341

**Published:** 2025-05-27

**Authors:** Feng Qu, Jiachen Weng, Xiufan Wu, Shuquan Zhang, La Li, Xuqin Guo, Hongrui Liu, Ying Chen

**Affiliations:** 1 Department of Medicinal Chemistry, School of Pharmacy, Fudan University, Shanghai 201203, China; 21211030007@m.fudan.edu.cn (F.Q.); 23211030035@m.fudan.edu.cn (X.W.); 22211030103@m.fudan.edu.cn (S.Z.); 24211030068@m.fudan.edu.cn (L.L.); 24111030029@m.fudan.edu.cn (X.G.); 2 Department of Pharmacology, School of Pharmacy, Fudan University, Shanghai 201203, China; jachen_weng@126.com

**Keywords:** nitric oxide, collateral sensitivity, structure–activity relationships, lysosomes, autophagy

## Abstract

In this study, twenty-four new furoxan and seco-coumarin hybrids were synthesized, and their antiproliferative activities against four breast cancer cells (MCF-7/ADR, MCF-7, MDA-MB-231, and MDA-MB-468) were evaluated. Among them, compound **9e** exhibited significant toxicity against MCF-7/ADR cells compared to MCF-7 cells, with a 1401-fold increase, indicating its high collateral sensitivity. Meanwhile, **9e** exhibited relatively lower toxicity to normal cell lines and improved solubility compared to the previous active compound, **4A93**, which features a coumarin integrity core. Preliminary pharmacological studies revealed that **9e** might be a potential P-glycoprotein substrate, which enters the lysosomes of MCF-7/ADR to release effective concentrations of nitric oxide, producing reactive oxygen species and inducing apoptosis. Moreover, laser confocal microscopy and Western Blot experiments showed that **9e** could induce autophagy in MCF-7/ADR cells. Additionally, the anti-tumor activity of compound **9e** could be inhibited by the ferroptosis inhibitor Fer-1. These results suggest that the remarkable antiproliferative potency of these hybrids in MCF-7/ADR may be related to multiple anticancer mechanisms. As a novel nitric oxide donor, compound **9e** was used to explore the potential development of an anti-tumor candidate with special pharmacological mechanisms to overcome multidrug resistance in breast cancer.

## 1. Introduction

Breast cancer (BC) remains a serious issue worldwide, and the American Cancer Society (ASC) estimated that new cases of BC will account for 32% of the total female cases in the United States in 2025 [1]. Treatments for BC, such as surgery, radiotherapy, chemotherapy, endocrine therapy, targeted therapy, and antibody–drug conjugates, have made great progress in the past 25 years [2,3,4,5], with chemotherapy remaining the main treatment method. However, multidrug resistance (MDR) is responsible for almost 90% of deaths during chemotherapy [6]. Among diverse and complex mechanisms causing MDR, ATP-binding cassette (ABC) transporters—including P-glycoprotein (P-gp, ABCB1), ABCC1, and ABCG2—have been confirmed to be closely associated with MDR and overexpressed in many MDR cancers [7,8,9]. Lina et al. reported that tumor cells under stress not only upregulate the expression of P-gp but also localize P-gp from the plasma membrane to the surface of lysosomes via endocytosis, leading to the capture of anti-tumor drugs such as doxorubicin (DOX) by lysosomes [10]. Jansson et al. found that di-2-pyridylketone-4,4-dimethyl-3-thiosemicarbazone (Dp44mT) and its derivatives were able to accumulate in the lysosomes of drug-resistant tumor cells by P-gp on lysosomes [11]. It is interesting that Dp44mT formed complexes to produce reactive oxygen species (ROS) through redox reactions in lysosomes, which further caused an increase in lysosomal membrane permeability and damage, ultimately inducing tumor cell apoptosis [10]. The lysosomal P-gp hijacking mechanism has piqued our interest and sparked ideas for in-depth research.

**Figure 1:**
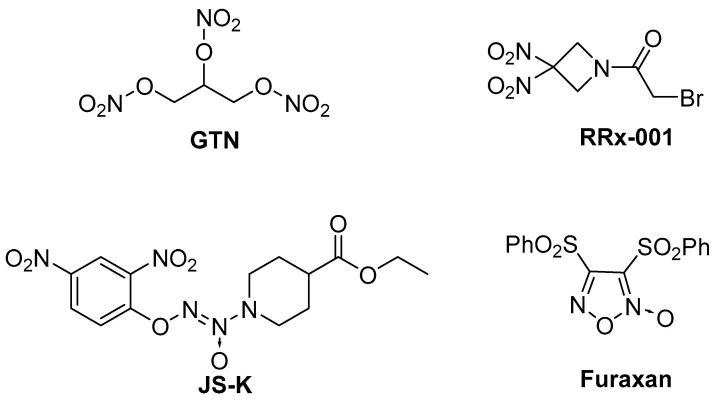
The chemical structures of several representative NO donors.

It is well known that nitric oxide (NO) is a special small molecule that easily forms ROS in vivo and influences both physiological and pathological processes [12,13]. One study showed that high concentrations of NO could suppress tumor growth by triggering DNA damage and apoptotic pathways [14]. Diverse NO donor compounds have been explored for therapeutic applications (Figure 1) [15], with furoxan and benzofuroxan hybrids emerging as prominent candidates in anti-cancer drug development. NOBF_4_ was reported as a mild nitrosating agent to selectively diazotizate 4-aminofuroxan, and the obtained diazonium salt was easy to separate and could be subsequently coupled with electron-donating aromatic hydrocarbons or acids [16]. Due to high levels of NO, furoxan derivatives can downregulate HIF-1α expression and AKT, ERK, and NF-κB activation, thereby overcoming MDR in tumors [17].

**Figure 2:**
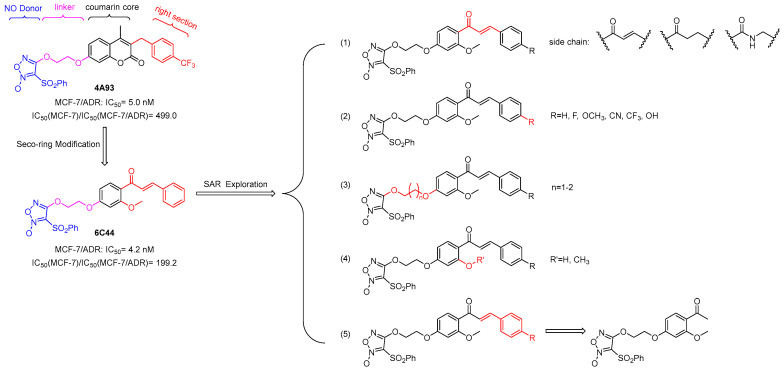
Structural modification strategies for compounds **4A93** and **6C44**.

In our previous research, coumarin/furoxan hybrids demonstrated potent antiproliferative activity against multiple tumor cell lines [18]. Through systematic structure–activity relationship (SAR) investigations of these compounds, we designed and synthesized compound **4A93**, a hybrid of furoxan and 4-trifluoromethyl coumarin. Intriguingly, this compound exhibited significant collateral sensitivity in drug-resistant MCF-7/ADR compared to MCF-7 (Figure 2) [19]. Its pharmacological mechanism was related to NO release and ROS formation in lysosomes, finally inducing cancer cell apoptosis [19]. However, the poor solubility of the coumarin/furoxan hybrids precluded more extensive mechanistic studies. Meanwhile, whether the coumarin skeleton is a crucial factor in maintaining high selective inhibition activity is still unclear. Hence, we tried to open the coumarin ring and synthesized seco-coumarin/furoxan derivative **6C44** (Figure 2) [20]. This compound also had a good selective inhibition activity, suggesting that the integrity of the coumarin skeleton is not a necessary condition for maintaining its highly selective inhibitory activity [20]. Therefore, this study aimed to optimize the SAR discussions, improve water solubility, and elucidate detailed pharmacological mechanisms of these seco-coumarin/furoxan hybrids. The specific research contents are summarized as follows: 1. Various side chains, such as *α*, *β*-unsaturated carbonyl groups, *α*, *β*-saturated carbonyl groups, and amide linkers, were used to sustain the spatial skeleton like the right side of coumarin. 2. Substituents that were previously reported as favorable for improving collateral sensitivity to MCF-7/ADR were incorporated. 3. The linker length between the left benzene ring and furoxan was extended to 3C. 4. The methoxy group was replaced with a hydroxy group to evaluate its impact on biological activity and solubility. 5. The right-side phenyl ring was removed to assess its necessity for maintaining both inhibitory activity and compound selectivity against MCF-7/ADR cells. Herein, twenty-four novel compounds were synthesized, and their cytotoxicity in vitro and water solubility were tested. The pharmacological mechanism of the desirable lead was further explored to develop novel structural compounds with better collateral sensitivity and water solubility to MDR cancer.

## 2. Results and Discussion

### 2.1. Chemistry

**Scheme 1:**
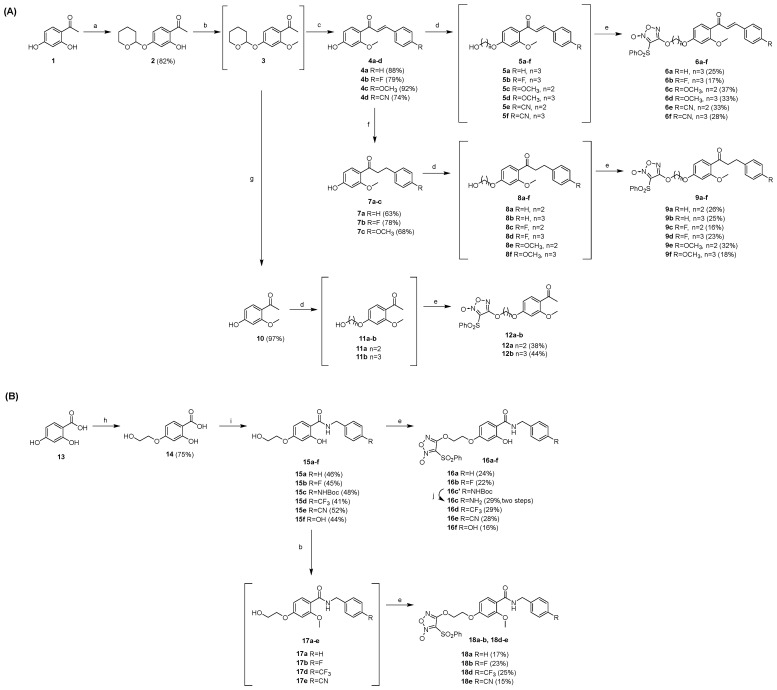
(**A**) Synthesis of target compounds **6a**-**f**, **9a**-**f**, **and 12a**-**b**: (a) DHP, PPTS, DCM, r.t., 12 h; (b) K_2_CO_3_, CH_3_I, DMF, 80 °C, 2 h; (c) benzaldehyde/4-fluorobenzaldehyde/4-methoxybenzaldehyde/4-formylbenzonitrile, NaOH, MeOH, 40 °C, 1.5 d; then 4 M HCl, r.t., 12 h; (d) bromoethanol/bromopropanol, K_2_CO_3_, NaI, DMF, 90 °C, 18 h; (e) phenylsulfonylfuroxan, dry DCM, DBU, r.t., 16–20 h; (f) 5%Pd/C, H_2_, Ph_2_S, MeOH, 12 h; (g) 4 M HCl, MeOH, r.t., 12 h. (**B**) Synthesis of target compounds **16a**-**f**, **18a**-**b**, and **18d**-**e**: (h) bromoethanol, NaOH, NaI, EtOH, 60 °C, 12 h; (i) NHS, DCC, 1,4-dioxane, N_2_; then benzylamine/4-fluorobenzylamine/*N*-Boc-4-aminobenzylamine/4-(trifluoromethyl)benzylamine/4-cyanobenzylamine/4-hydroxybenzylamine, NaHCO_3_, H_2_O, 60 °C, 18 h; (j) TFA, r.t., 2 h.

Synthesis routes of twenty-four target compounds (**6a**-**f**, **9a**-**f**, **12a**-**b**, **16a**-**f**, **18a**-**b**, and **18d**-**e**) are illustrated in Scheme 1. 2,4-Dihydroxyacetophenone (**1**) was treated with 3,4-dihydro-2*H*-pyran (DHP) to get 4-hydroxy protected compound **2** under the catalysis of pyridinium p-toluenesulphonate (PPTS). **2** was methylated with CH_3_I to give **3**, which was followed by an aldol condensation with several benzaldehydes containing different substituents. Seco-coumarin intermediates **4a**-**d** with *α*, *β*-unsaturated ketone side chains were obtained through the removal of the protecting group of the above condensation products under 4 M HCl. In the presence of K_2_CO_3_ and NaI, intermediates **4a**-**d** underwent etherification with 2-bromoethanol or 3-bromo-1-propanol to give **5a**-**f**, which were coupled with phenylsulfonylfuroxan to get target compounds **6a**-**f** under the catalysis of 1,8-diazabicyclo[5.4.0]-7-undecene (DBU) in dichloromethane at room temperature, yielding 17–37% (two steps). To obtain compounds with *α*, *β*-saturated ketone side chain, **4a**-**c** were reduced in a hydrogen atmosphere with 5% Pd/C and diphenyl sulfide of catalytic amount, getting **7a**-**c**. After the same procedures as **4a**-**d** to **6a**-**f**, target seco-coumarin/furoxan coupling compounds **9a**-**f** were synthesized from the intermediates **7a**-**c**, yielding 16–32% (two steps). Moreover, intermediate **3** was removed from its protecting group directly under 4M HCl, getting **10**, and followed by etherification and a coupling reaction to form target compounds **12a**-**b**, yielding 38–44% (two steps) (Scheme 1A).

Meanwhile, 2,4-dihydroxybenzoic acid (**13**) reacted with 2-bromoethanol at 60 °C in ethanol to gain 2-hydroxy-4-(2-hydroxyethoxy) benzoic acid (**14**). Then, in the presence of *N*, *N*-dicyclohexylcarbodiimide (DCC), **14** was condensed with *N*-hydroxysuccinimide (NHS) to form an activated ester, which directly reacted with differently substituted benzylamines after filtration to produce key intermediates **15a**-**f** with an amide side chain. After coupling with phenylsulfonylfuroxan, intermediates **15a**-**f** were transformed to compounds **16a**-**b**, **16c’**, and **16d**-**f**, yielding 16–29%, where **16c’** was subsequently deprotected in TFA to afford the target compound **16c**. Furthermore, **15a**-**b** and **15d**-**e** were treated with CH_3_I, under the catalysis of K_2_CO_3_ in *N*, *N*-dimethylformamide (DMF) at room temperature to provide 2-methoxy seco-coumarin derivatives **17a**-**b** and **17d**-**e**. Finally, target compounds **18a**-**b** and **18d**-**e** were synthesized through a coupling reaction, yielding 15–25% (two steps) (Scheme 1B). All target compound structures have been assessed by ^1^H NMR, ^13^C NMR, and HRMS(ESI).

### 2.2. In Vitro Cytotoxicity Activity

Twenty-four seco-coumarin/furoxan hybrids (**6a**-**f**, **9a**-**f**, **12a**-**b**, **16a**-**f**, **18a**-**b**, and **18d**-**e**) were tested for their antiproliferative activity in MCF-7 (human breast cancer cell line), MCF-7/ADR (doxorubicin-resistant human breast cancer cell line), MDA-MB-231, and MDA-MB-468 (human triple-negative breast cancer cell line), with **4A93** and doxorubicin as references. As listed in Table 1, compared to **4A93**, most of the newly synthesized compounds showed similar or higher antiproliferative activity with respect to MCF-7/ADR, with IC_50_ values ranging from 1.09 to 91.1 nM. Notably, compounds with *α*, *β*-unsaturated ketone (**6a**-**f**) or *α*, *β*-saturated ketone side chain (**9a**-**f**) exhibited significant inhibitory activity, with all their IC_50_ values lower than 10 nM except for compound **6c**. Conversely, compounds **12a**-**b**, whose benzene rings were removed from the side chains, showed an obvious decrease in antiproliferative activity. Their IC_50_ values were 25–50-fold lower than that of the corresponding compounds **6a** and **9a**-**b**. These results implied that the side chain, including a phenyl group, might play an important role in seco-coumarin/furoxan derivatives inhibiting MCF-7/ADR. Moreover, when replacing the coumarin ring with an amide side chain, the compounds **16a**-**f**, **18a**-**b**, and **18d**-**e** showed strong antiproliferative activity as well. Although most seco-coumarin/furoxan derivatives (**16a**-**f**, **18a**-**b,** and **18d**-**e**) with the amide side chain exhibited lower antiproliferative activity against MCF-7/ADR than the two series of compounds with *α*, *β*-unsaturated ketones or saturated ketone side chains mentioned above, four compounds (**16d**-**e** and **18d**-**e**) with 4-trifluoromethyl or cyanide substituted at phenyl on the amide side chain presented comparable activity, with IC_50_ values of 10.53, 5.36, 6.56, and 19.87 nM, respectively.

Regarding MDA-MB-231 and MDA-MB-468 cell lines, all target compounds exhibited moderate cytotoxicity against both. Many of them had IC_50_ values between 1 and 6 μM toward MDA-MB-231 and 80 and 780 nM for MDA-MB-468, which were far lower than the IC_50_ values in MCF-7/ADR. These results indicated that newly synthesized compounds retain high selective cytotoxicity against MCF-7/ADR cell lines.

Tumor cells that develop resistance to certain drugs may exhibit increased sensitivity to other drugs, which is known as collateral sensitivity [19]. In this research, the ratio of IC_50_ against MCF-7 and MCF-7/ADR was calculated to evaluate compounds’ collateral sensitivity. As depicted in Table 1, compounds with the *α*, *β*-saturated ketone side chain **9a**-**f** showed the best selectivity between MCF-7/ADR and MCF-7, among which **9a**-**b** and **9d**-**e** had a selectivity ratio (IC_50(MCF-7)_/IC_50(MCF-7/ADR)_) exceeding 1000-fold. Meanwhile, despite the similar antiproliferative activity towards MCF-7/ADR of **6a**-**f** and **9a**-**f**, the collateral sensitivity of the compounds containing saturated ketone side chains was clearly higher than that of those including *α*, *β*-unsaturated ketone side chain. Part of the reason for this result could be attributed to the compounds **6a**-**f** having higher cytotoxicity in MCF-7 than that of **9a**-**f**, while there was no significant difference in MCF-7/ADR. And in compounds with amide side chains **16a**-**f**, **18a**-**b**, and **18d**-**e**, the collateral sensitivity of them was generally decreased. But 4-cyanide phenyl and amide side chain substitutional compound **16e** had a selectivity ratio of 561.9-fold, which was higher than the 499-fold of reference **4A93**. Given that both amide side chains and cyanide groups are beneficial for improving solubility, this series of seco-coumarin/furoxan derivatives deserves further optimization in future research.

The cytotoxicity of twelve compounds (**6a**, **6c**-**e**, **9a**, **9e**-**f**, **12a**, **16d**-**e**, and **18d**-**e**) covering different side chains, substituents, and linker lengths was further evaluated in human normal mammary epithelial cell lines MCF-10A. As displayed in Table 2, the data revealed that all selected seco-coumarin/furoxan derivatives had low toxicity, with IC_50_ values ranging from 1.8 to 13.4 μM, which were more than 100 times lower than the IC_50_ values for MCF-7/ADR, showing good safety. Among them, compounds **6d** and **9e** had the lowest cytotoxicity. Their IC_50_ values were 13.4 and 13.2 μM, separately. Moreover, six compounds (**6a**, **6d**-**e**, **9a**, **9e**, and **12a**) were screened for cytotoxicity in HUVEC (human umbilical vein endothelial cell lines) at a 500 nM concentration (see Figure S1). The data showed that compounds **9e** and **12a** exhibited the highest safety, with a cell survival probability over 90%.

Based on the antiproliferative activity, preliminary SAR could be inferred. First, compared to compounds (**16a**-**f**, **18a**-**b**, and **18d**-**e**) with amide side chains, seco-coumarin/furoxan derivatives with *α*, *β*-unsaturated ketone (**6a**-**f**) or *α*, *β*-saturated side chains (**9a**-**f**) exhibited stronger antiproliferative activities against drug-resistant MCF-7/ADR, while removing the phenyl from the side chain, as compounds **12a**-**b**, would lead to a decrease in the activity. Second, most synthesized compounds retained collateral sensitivity in MCF-7/ADR. Significantly, compounds **9a**-**b** and **9d**-**e** revealed an over 1000-fold selectivity ratio against MCF-7/ADR and MCF-7, which is higher than the ratio of lead **4A93** of 499-fold, indicating that the integrity of the coumarin lactone ring is not a necessary condition for maintaining compound activity and collateral sensitivity. Third, compounds with a 3C-linker (**6d**, **6f**, **9d** and **9f**) generally showed higher cytotoxicity towards both MA and MCF-7 cell lines than compounds with a 2C-linker (**6c**, **6e**, **9c**, and **9e**), but the selectivity ratio (IC_50 (MCF-7)_/IC_50 (MCF-7/ADR)_) increases unevenly. Fourth, the toxicity against MCF-10A of compounds containing electron-donating groups, including methoxy (**6d** and **9e**), on the phenyl of the side chain is lower than that of compounds bearing electron-withdrawing groups, including cyano and trifluoromethyl (**16d**-**e** and **18d**-**e**). As to HUVEC, the cytotoxicity of the tested compounds is like that of MCF-10A, showing overall low toxicity and good safety.

### 2.3. Solubility

To find a lead compound with better solubility than **4A93**, the solubility of all twenty-four synthesized compounds in a mixture of water and acetonitrile was tested, since their solubility was too poor to be measured in pure water. Compared to **4A93**, the synthesized compounds showed 24- to 775-fold higher solubility (Table 3). Among them, compounds **16c** and **18c** containing an amino group on the benzene ring of the amide side chain showed excellent solubility with 409- and 775-fold higher than that of **4A93**, indicating that the introduction of hydrophilic group might be favorable to further enhance solubility.

Although the solubility of compound **9e** with 1402-fold collateral sensitivity was relatively weaker than that of compounds with amide chains, it still had an obvious improvement of 71-fold over that of **4A93**. Overall, due to their flexible side-chain skeletal structure, seco-coumarin/furoxan derivatives had better solubility while retaining significant antiproliferative activity. Such a seco-skeleton might be a more promising structure than that of the coumarin core for further exploring and developing a desirable lead.

Therefore, considering factors such as antiproliferative activity, collateral sensitivity, safety for normal cells, and solubility, compound **9e**, which has 4-methoxy phenyl on the *α*, *β*-saturated ketone side chain and a 2C linker, was selected as the optimization compound for subsequent pharmacological mechanism studies.

### 2.4. Pharmacological Study

#### 2.4.1. Compounds Released NO to Exert Anti-Tumor Proliferation Activity

**Figure 3:**
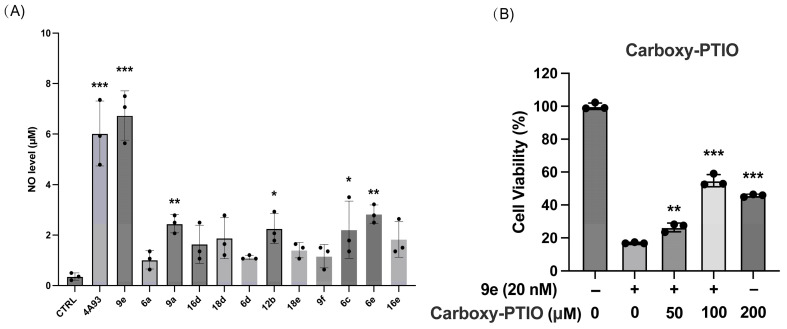
(**A**) The amount of nitric oxide released in MCF-7/ADR. (**B**) In MCF-7/ADR Carboxy-PTIO reduced the toxic effects of **9e**. Data (n = 3) were presented as mean ±SD, * *p* < 0.05, ** *p* < 0.01, *** *p* < 0.001, compared to the control group.

Since furoxan is a well-known NO donor, the total NO level of MCF-7/ADR incubated with twelve seco-coumarin/furoxan derivatives, **6a**, **6c**-**e**, **9a**, **9e**-**f**, **12a**, **16d**-**e**, and **18d**-**e**, was detected using the Griess assay. After the treatment of selected compounds in a dose of 20 nM, as depicted in Figure 3A, the NO concentrations were clearly higher than the original NO release level of the cells. The results suggested that there was a certain relationship between the anticancer potency of these NO donors and their release of NO. Furthermore, a NO clearance experiment was conducted using the NO scavenger Carboxy-PTIO (Figure 3B). The data showed that Carboxy-PTIO could concentration-dependently reduce the anti-tumor activity of **9e**. After treating with a 100 μM dose of Carboxy-PTIO, the antiproliferative activity of 20 nM concentration **9e** on MCF-7/ADR was obviously suppressed, with a cell survival rate of 53.7%. The experimental results indirectly indicated that the potency of **9e** against MCF-7/ADR was associated with its NO release level. A systematic investigation of the fundamental mechanisms of their collateral sensitivity to MCF-7/ADR remains a matter of great urgency.

#### 2.4.2. Compound **9e** Promoted Rh-123 Accumulation in MCF-7/ADR

**Figure 4:**
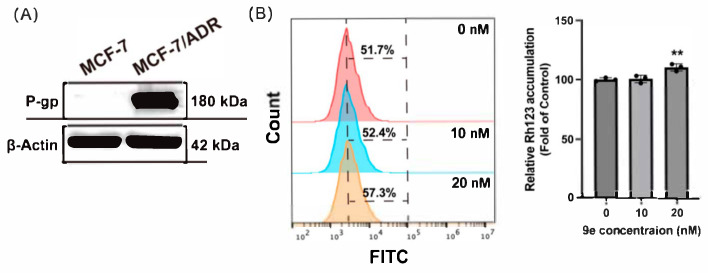
(**A**) P-gp was overexpressed in MCF-7/ADR. (**B**) **9e** enhanced the intracellular accumulation of Rh-123 in MCF-7/ADR. Data (n = 3) were presented as mean ± SD, ** *p* < 0.01, compared to the control group.

Rhodamine 123 (Rh-123) is a typical P-gp substrate that can be actively pumped out of cells by this transporter. As MCF-7/ADR exhibits P-gp overexpression (Figure 4A), we hypothesized that the antiproliferative activity of these compounds also contributed to the overexpression of P-gp, similar to **4A93**. Hence, using DMSO as a blank control, we performed the Rh-123 accumulation assay in MCF-7/ADR. The results showed that Rh-123 accumulated significantly when culturing cells with **9e** at a dose of 20 nM (Figure 4B), indicating that **9e** was a potential P-gp substrate. This suggested that the high collateral sensitivity of the synthesized compounds in MCF-7/ADR might rely on the mechanism whereby the compounds entered lysosomes by overexpressing P-gp and inducing apoptosis, as previously reported. Next, we indirectly observed whether compound **9e** entered the lysosomes using a Lyso-NO probe.

#### 2.4.3. Compound **9e** Selectively Emitted NO Within Lysosomes of MCF-7/ADR

**Figure 5:**
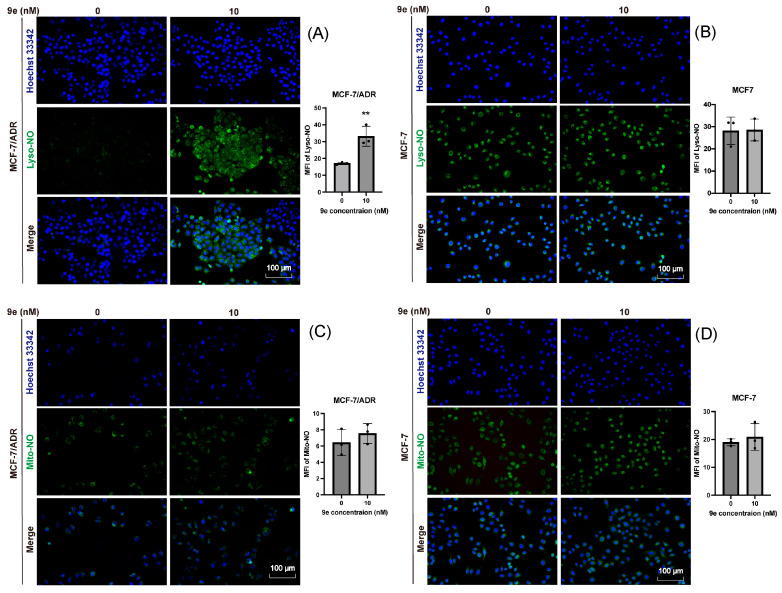
The fluorescence intensity of (**A**) Lyso-NO probe in MCF-7/ADR. (**B**) Lyso-NO probe in MCF-7. (**C**) Mito-NO probe in MCF-7/ADR. (**D**) Mito-NO probe in MCF-7. The NO probe is shown in green and nuclei are shown in blue. Scare bar = 100 μm. Data (n = 3) were presented as mean ± SD, ** *p* < 0.01, compared to the control group.

To investigate the specific subcellular organelles affected by **9e**, Lyso-NO probe and Mito-NO probe were used to selectively capture NO in the lysosomes or mitochondria of MCF-7/ADR, respectively. A significant rise in the Lyso-NO probe signal was observed following 10 nM **9e** treatment, demonstrating lysosomal NO generation compared to vehicle-treated cells. However, the Mito-NO probe signal showed no difference between the administration group and the control, indicating that **9e** accumulated in the lysosomes of MCF-7/ADR rather than in the mitochondria (Figure 5A,C). The experimental results further demonstrated that **9e** was a potential substrate of P-gp and might accumulate in lysosomes through the overexpressed P-gp to exert its antiproliferative activity in MCF-7/ADR. Meanwhile, no increase in fluorescence intensity was observed in either lysosomes or mitochondria of MCF-7 cell lines (Figure 5B,D), which was consistent with the low expression of P-gp on the lysosomal membrane of MCF-7 cell lines and the poor cytotoxicity of **9e** in MCF-7 (IC_50_ = 4227 nM). The outcomes indicated that the high collateral sensitivity of **9e** was closely related to its entry into lysosomes, releasing NO.

#### 2.4.4. Compound **9e** Emitted NO to Generate ROS and Induce Apoptosis in MCF-7/ADR

**Figure 6:**
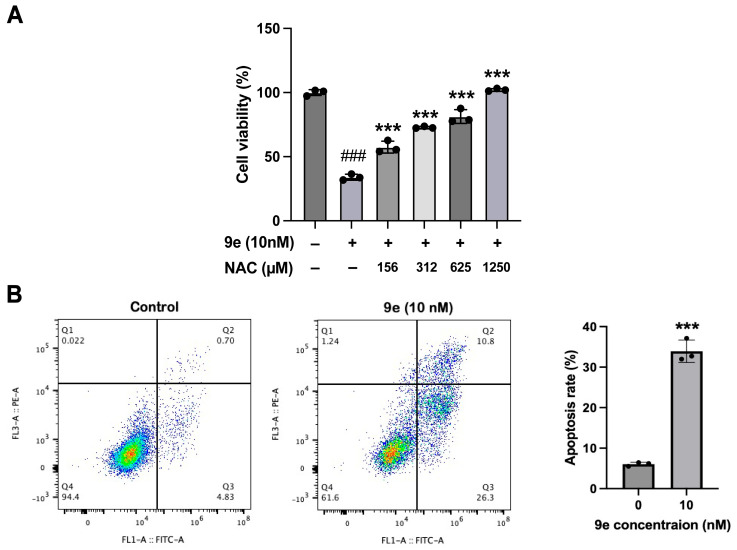
(**A**) The cytotoxicity of **9e** was reduced by NAC in MCF-7/ADR. Data (n = 3) were presented as mean ± SD, ### *p* < 0.001 compared to the control group, *** *p* < 0.001, compared to the 9e-only group. (**B**) **9e** promoted cell apoptosis in MCF-7/ADR, the summation of upper-right and lower-right quadrants were considered apoptotic populations. Data (n = 3) were presented as mean ± SD, *** *p* < 0.001, compared to the control group.

Given NO’s dual role as a signaling molecule and regulator of ROS homeostasis, a typical ROS scavenger, NAC, was used to examine the relationship between ROS concentration and the inhibitory activity of **9e**. After the pretreatment of MCF-7/ADR with NAC, the proliferation inhibition rate of **9e** significantly decreased and positively correlated with the NAC concentration (Figure 6A). When the concentration of NAC reached 1250 μM, **9e** at a concentration of 10 nM completely lost its inhibitory activity on MCF-7/ADR cells, which indicated that the inhibitory activity of **9e** had a close relationship with the formation of ROS. According to our previous research [19], ROS can break the stability of the lysosomal membrane and promote cell apoptosis. Again, flow cytometry was conducted using MCF-7/ADR treated with 10 nM of **9e** and stained with Propidium Iodide (PI) and Annexin V-FITC (Figure 6B). The results showed that **9e** significantly promotes the apoptosis of MCF-7/ADR from 5.53% to 37.1% (Q2+Q3). Overall, we speculated that the pharmacological mechanism of **9e** might contribute to its entry into lysosomes to release NO, further producing ROS and inducing apoptosis.

#### 2.4.5. Effect of P-gp Inhibitor on Antiproliferative Activity of **9e**

**Figure 7:**
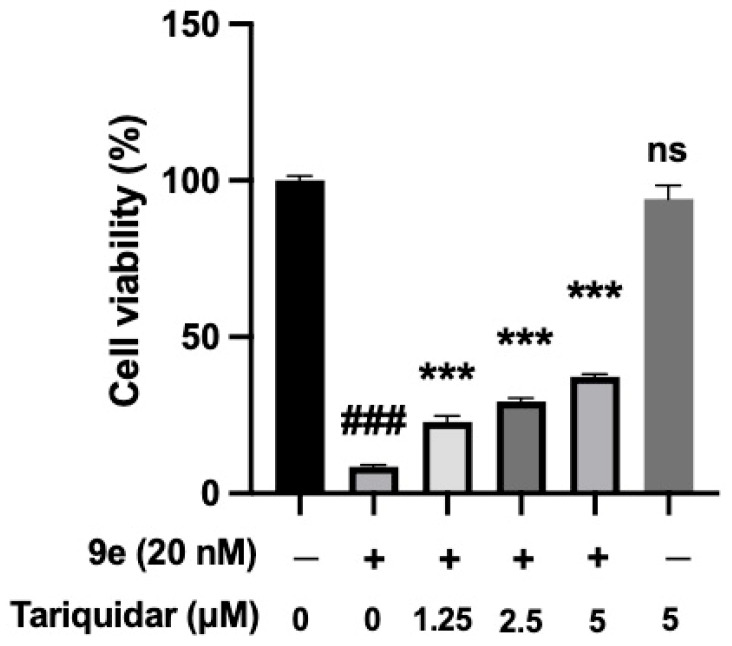
The cytotoxicity of **9e** was partly reduced by tariquidar in MCF-7/ADR. Data (n = 3) were presented as mean ± SD, ### *p* < 0.001 compared to the control group, *** *p* < 0.001, compared to the **9e**-only group.

To further investigate the relationship between P-gp and the antiproliferative activity of **9e**, we treated cells with tariquidar, a common P-gp inhibitor, to find whether the cytotoxicity of **9e** could be blocked by the inhibitor (Figure 7). However, even when the concentration of tariquidar reached 5 μM, the activity of **9e** was not completely lost. A total of 62% of MCF-7/ADR cells were still inhibited. The results indicated the addition to the pathway related to P-gp, which prompted us to explore more pharmacological mechanisms involving autophagy and ferroptosis in the next study.

#### 2.4.6. Compound **9e** Induced Autophagy in MCF-7/ADR

**Figure 8:**
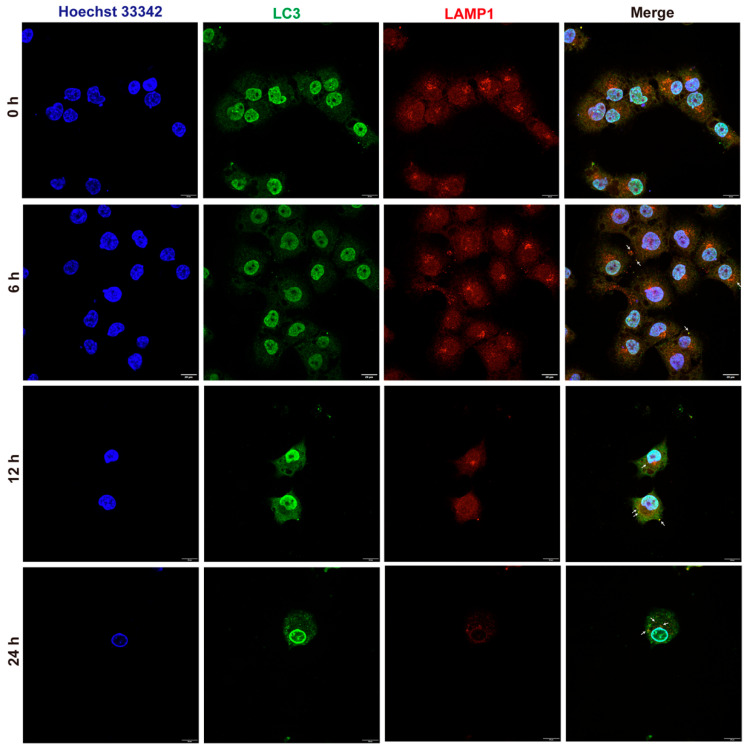
Autophagy flow in MCF-7/ADR. Scare bar = 20 μm.

Autophagy is the process by which autophagosomes combine with lysosomes, degrading their contents and maintaining cellular homeostasis. Since **9e** was highly likely to impact the lysosomes of MCF-7/ADR, laser confocal microscopy was used to observe the dynamic changes in the autophagy process in MCF-7/ADR induced by **9e** (Figure 8). After co-incubating a 20 nM concentration of **9e** with MCF-7/ADR for 0, 6, 12, and 24 h, the cells were subjected to immunofluorescence staining using LC3 and LAMP1 antibodies. At 6 h, there was no significant change in the fluorescence intensity of LC3 and LAMP1, but autophagosomes gradually showed good colocalization with lysosomes (white arrow), indicating that MCF-7/ADR produced autophagosomes under the induction of **9e**. At 12 and 24 h, the fluorescence of LC3 increased, indicating the accumulation of autophagosomes. Simultaneously, the fluorescence of LAMP1 weakened, indicating a decrease in lysosomal activity. It is inferred that compound **9e** could induce the production of LC3 autophagosomes in MCF-7/ADR. However, due to the dysfunction of lysosomes caused by NO accumulation, damaged lysosomes may undergo lysosomal autophagy and fail to degrade the LC3-II protein on the inner membrane of autophagosomes, resulting in the accumulation of autophagosomes and weakened lysosomal activity.

**Figure 9:**
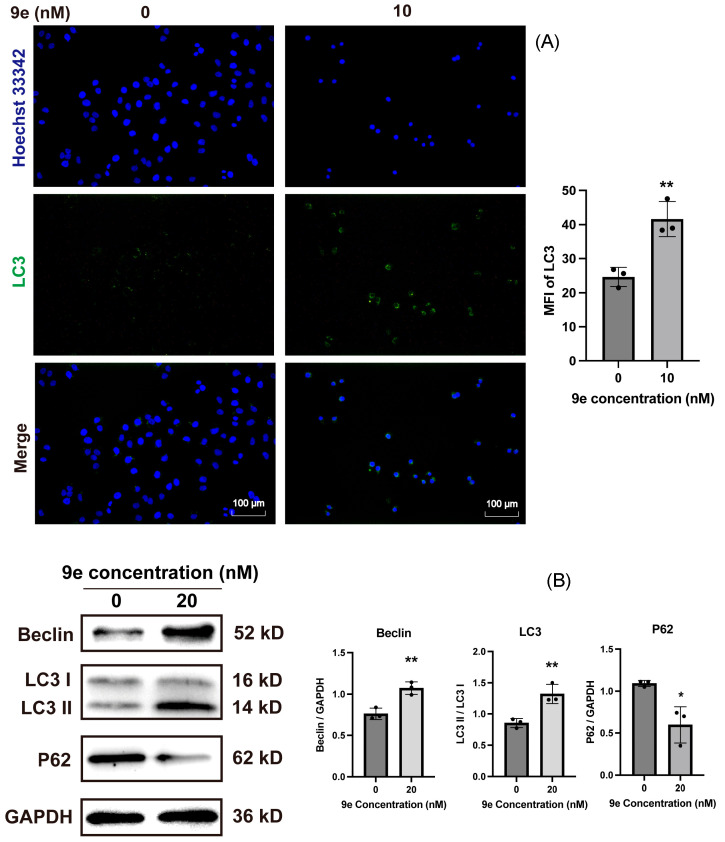
(**A**) **9e** induced the increase in LC3 in MCF-7/ADR. The nuclei are shown in blue and LC3 is shown in green. Scare bar = 100 μm. (**B**) **9e** regulated the expression of autophagy-related proteins. Data (n = 3) were presented as mean ± SD, * *p* < 0.05, ** *p* < 0.01, compared to the control group.

LC3 is a critical marker protein in the autophagy signaling pathway associated with the formation, fusion, and degradation of autophagosomes. After treatment of MCF-7/ADR cells with **9e** (10 nM) for 24 hours, the fluorescence intensity of LC3 significantly increased (Figure 9A). Furthermore, it was observed that **9e** regulated the expression of autophagy-related proteins such as LC3I, LC3II, and P62 (Figure 9B). These results indicated that a mechanism of **9e**, by inducing autophagy, might also contribute to its strong proliferation activity against MCF-7/ADR.

#### 2.4.7. Compound **9e** Promoted Ferroptosis in MCF-7/ADR

**Figure 10:**
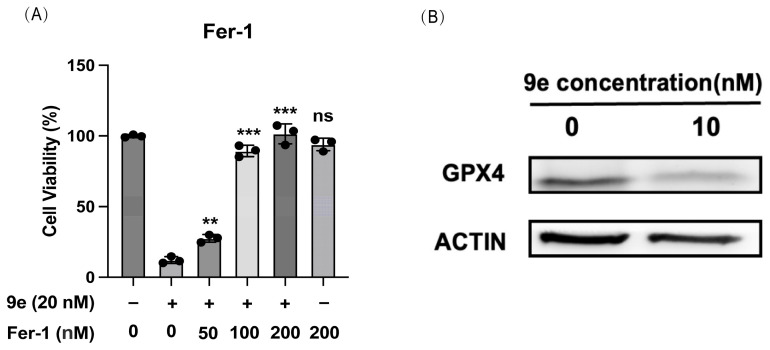
(**A**) The cytotoxicity of **9e** was reduced by Fer-1 in MCF-7/ADR. Data (n = 3) were presented as mean ± SD, ** *p* < 0.01, *** *p* < 0.001, compared to the control group. (**B**) **9e** down-regulated GPX4 expression.

According to the experiments mentioned above, the release of an effective concentration of NO played an important role in the anticancer action of **9e**. In our previous research, we explored and found that the antiproliferative of coumarin–furoxan hybrids might depend on the ferroptosis pathway in non-small cell lung cancer [21]. Therefore, we proposed a mechanism whereby compound **9e** could also consume intracellular GSH to release NO, disrupt the intracellular redox balance, and induce ferroptosis. As depicted in Figure 10A, the proliferation inhibition rate of **9e** gradually decreased as the concentration of a ferroptosis inhibitor, Fer-1, increased. When cells were treated with **9e** in a dose of 20 nM, the proliferation inhibition rate was 89%. After pretreatment with Fer-1 at a concentration of 200 nM on MCF-7/ADR, the rate decreased to 2%, indicating that the antiproliferative activity of **9e** was almost lost. Moreover, the expression of GPX4, a ferroptosis-related protein, clearly decreased after the treatment of **9e** (Figure 10B), suggesting that **9e** might inhibit the production of GSH by downregulating GPX4, further inducing lipid peroxide accumulation, and causing ferroptosis.

#### 2.4.8. Compound **9e** Inhibition of hERG Potassium Channels

hERG potassium channels underlie the cardiac rapid delayed rectifier (IKr) potassium current [22], an important target for evaluating the cardiac toxicity of compounds. Therefore, the inhibition of **9e** against hERG potassium channels was tested using the patch clamp method with Cisapride as a reference compound. As listed in Table 4, compound **9e** exhibited an IC_50_ value exceeding 40 μM, indicating weak cardiac toxicity and excellent in vivo safety.

## 3. Experimental Section

### 3.1. General

The mass spectrometry of the compound was determined using Agilent Technologies 1260 Infinity LC/MS (Agilent Technologies, Santa Clara, CA, USA). The melting point of the target compound was determined using the SGW X-4 micro melting point analyzer (Shanghai Precision Instrument, Shanghai, China). 1H NMR was measured using a Varian Mercury Plus 400 MHz NMR spectrometer (Agilent Technologies, Santa Clara, CA, USA) and a Bruker 600 MHz NMR spectrometer (Bruker, Karlsruhe, Germany). 13C NMR was measured using a Bruker 150 MHz nuclear magnetic resonance spectrometer (Bruker, Karlsruhe, Germany). The HPLC detection was conducted by the LC-15C detector (Shimadzu Corporation, Kyoto, Japan), and the Phenomenex C18 chromatographic column (5 μm particle size silica, 250 mm × 4.6 mm, Phenomenex, Torrance, CA, USA). TLC monitoring reaction was performed using a silica gel HSGF/UV 254 UV detector (Merk, Darmstadt, Germany).

Intermediates **2**, **3**, **4**, **7**, **10**, **14**, and **15** were characterized via ^1^H NMR and MS, confirming that their structures matched the theoretical products before proceeding to subsequent reactions. All 24 target compounds were fully characterized via ^1^H NMR, ^13^C NMR, and HRMS, and their melting points were determined.

### 3.2. Synthetic Procedure and Spectral Data

**1-(2-hydroxy-4-((tetrahydro-2*H*-pyran-2-yl)oxy)phenyl)ethan-1-one (2)**

1-(2,4-Dihydroxyphenyl) ethan-1-one (**1**) (50 mmol) and PPTS (2 mmol, 0.04 eq.) were dissolved in DCM (70 mL), with the addition of DHP (11.6 mL, 2.5 eq.). The solution was stirred at room temperature for 12 h. The reaction mixture was washed with NaHCO_3_ (aq.) and brine, dried over anhydrous Na_2_SO_4_, and concentrated under reduced pressure. The concentrated solution was added with PE and ultrasonically shock to give a white solid, which was filtered and dried in an infrared oven to yield **2**.

Yield: 82%. White solid. **^1^H NMR** (400 MHz, CDCl_3_) δ 12.61 (s, 1H, -ArOH), 7.63 (d, *J* = 8.7 Hz, 1H, 6-ArH), 6.65–6.50 (m, 2H, 3,5-ArH), 5.48 (t, *J* = 3.0 Hz, 1H, -OCH-O-), 3.88–3.56 (d, 2H, -CH_2_CH_2_O-), 2.56 (s, 3H, -COCH_3_), 2.07–1.47 (m, 6H, -CH_2_-). **ESI**-**MS** *m*/*z* 237.4 [M + H]^+^.

**1-(2-methoxy-4-((tetrahydro-2*H*-pyran-2-yl)oxy)phenyl)ethan-1-one (3)**

Compound **2** (12 mmol) was dissolved in DMF (10 mL), with the addition of K_2_CO_3_ (60 mmol, 5 eq.). The solution was heated to 80 °C, with stirring for 0.5 h, and then CH_3_I solution (1.5 mL, 2 eq.) was added dropwise, and the reaction was kept at 80 °C for 1.5 h and monitored via TLC. The resulting mixture was filtered, extracted with EA, washed with brine, dried, and concentrated to obtain crude product **3** (2.61 g), which was directly used in the next step without further purification. Part of the crude product was purified via column chromatography to identify its structure.

**^1^H NMR** (400 MHz, CDCl_3_) δ 7.80 (d, *J* = 8.8 Hz, 1H, 6-ArH), 6.71–6.60 (m, 2H, 3,5-ArH), 5.50 (t, *J* = 3.1 Hz, 1H, -OCH-O-), 3.89 (s, 3H, -OCH_3_), 3.87–3.62 (d, 2H, -CH_2_CH_2_O-), 2.57 (s, 3H, -COCH_3_), 2.06–1.56 (m, 6H, -CH_2_-). **ESI**-**MS** *m*/*z* 251.1 [M + H]^+^.

#### 3.2.1. General Procedure for **4a**-**d**

The reaction mixture of **3** (5 mmol), NaOH (12 mmol, 2.4 eq.), and the corresponding substituted benzaldehyde (12 mmol, 2.4 eq.) in methanol (10 mL) was stirred at 40 °C for 1.5 days. Then, the pH of the solution was adjusted to below 3 using 4 N HCl. The mixture was stirred at room temperature for 12 h. After removing part of the methanol from the solution via vacuum distillation, the solution was extracted with EA, washed with brine, dried, and concentrated in a vacuum. The concentrated solution was added with a mixture of PE and EA and ultrasonically shocked to yield solid products, which were filtered and dried in an infrared oven to create **4a**-**d**.

**(*E*)-1-(4-hydroxy-2-methoxyphenyl)-3-phenylprop-2-en-1-one (4a)**

Yield: 87.9%. Light-yellow solid. **^1^H NMR** (400 MHz, DMSO-*d*_6_) δ 13.51 (s, 1H, -ArOH), 7.81–7.66 (m, 3H, 4′,6-ArH, ArCH=CH-), 7.60 (m, 2H, 2′,6′-ArH), 7.54 (d, *J* = 15.6 Hz, 1H, ArCH=CH-), 7.43–7.37 (m, 2H, 3′,5′-ArH), 6.56–6.45 (m, 2H, 3,5-ArH), 3.84 (s, 3H, -OCH_3_). **ESI**-**MS** *m*/*z* 255.2 [M + H]^+^.

**(*E*)-3-(4-fluorophenyl)-1-(4-hydroxy-2-methoxyphenyl)prop-2-en-1-one (4b)**

Yield: 78.9%. Yellow solid. **^1^H NMR** (400 MHz, CDCl_3_) δ 7.70–7.62 (m, 2H, 6-ArH, ArCH=CH-), 7.59–7.55 (m, 2H, 2′,6′-ArH), 7.43 (d, *J* = 15.8 Hz, 1H, ArCH=CH-), 7.07 (t, *J* = 8.6 Hz, 2H, 3′,5′-ArH), 6.53–6.44 (m, 2H, 3,5-ArH), 3.84 (s, 3H, -OCH_3_). **ESI**-**MS** *m*/*z* 272.8 [M + H]^+^.

**(*E*)-1-(4-hydroxy-2-methoxyphenyl)-3-(4-methoxyphenyl)prop-2-en-1-one (4c)**

Yield: 92.5%. White solid. **^1^H NMR** (400 MHz, CDCl_3_) δ 7.73–7.65 (m, 2H, 6-ArH, ArCH=CH-), 7.56 (d, *J* = 8.3 Hz, 2H, 2′,6′-ArH), 7.39 (d, *J* = 15.6 Hz, 1H, ArCH=CH-), 6.92 (d, *J* = 8.3 Hz, 2H, 3′,5′-ArH), 6.54–6.47 (m, 2H, 3,5-ArH), 3.88 (s, 3H, 2-OCH_3_), 3.85 (s, 3H, 4′-OCH_3_). **ESI**-**MS** *m*/*z* 285.3 [M + H]^+^.

**(*E*)-4-(3-(4-hydroxy-2-methoxyphenyl)-3-oxoprop-1-en-1-yl)benzonitrile (4d)**

Yield: 74.2%. Light-yellow solid. **^1^H NMR** (400 MHz, CDCl_3_) δ 8.06 (d, *J* = 8.3 Hz, 1H, 6-ArH), 7.72 (d, *J* = 15.8 Hz, 1H, ArCH=CH-), 7.69–7.63 (m, 5H, ArH, ArCH=CH-), 6.55–6.48 (m, 2H, 3,5-ArH), 3.89 (s, 3H, -OCH_3_). **ESI**-**MS** *m*/*z* 279.9 [M + H]^+^.

#### 3.2.2. General Procedure for **7a**-**c**

To a solution of **4a**-**c** (4 mmol) in methanol (10 mL), Pd/C (5%, 20wt%) and a catalytic amount of Ph_2_S (0.01 eq.) were added. After replacing the atmosphere with a H_2_ balloon, the mixture was continuously stirred and monitored by TLC. The reacted solution was filtered, concentrated in a vacuum, and then purified via column chromatography to produce **7a**-**c**.

**1-(4-hydroxy-2-methoxyphenyl)-3-phenylpropan-1-one (7a)**

Yield: 63%. White solid. **^1^H NMR** (400 MHz, CDCl_3_) δ 7.76 (d, *J* = 9.0 Hz, 1H, 6-ArH), 7.33–7.15 (m, 5H, 2′-6′-ArH), 6.49–6.42 (m, 2H, 3,5-ArH), 3.84 (s, 3H, -OCH_3_), 3.28 (t, *J* = 7.7 Hz, 2H, ArCH_2_CH_2_-), 3.01 (t, *J* = 7.7 Hz, 2H, ArCH_2_CH_2_-). **ESI**-**MS** *m*/*z* 257.3 [M + H]^+^.

**3-(4-fluorophenyl)-1-(4-hydroxy-2-methoxyphenyl)propan-1-one (7b)**

Yield: 78%. White solid. **^1^H NMR** (400 MHz, CDCl_3_) δ 7.73 (d, *J* = 9.2 Hz, 1H, 6-ArH), 7.17 (t, *J* = 8.7 Hz, 2H, 2′,6′-ArH), 6.96 (t, *J* = 8.7 Hz, 2H, 3′,5′-ArH), 6.49–6.42 (m, 2H, 3,5-ArH), 3.84 (s, 3H, -OCH_3_), 3.25 (t, *J* = 7.7 Hz, 2H, ArCH_2_CH_2_-), 2.98 (t, *J* = 7.8 Hz, 2H, ArCH_2_CH_2_-). **ESI**-**MS** *m*/*z* 275.2 [M + H]^+^.

**1-(4-hydroxy-2-methoxyphenyl)-3-(4-methoxyphenyl)propan-1-one (7c)**

Yield: 68%. White solid. **^1^H NMR** (400 MHz, CDCl_3_) δ 7.75 (d, *J* = 9.2 Hz, 1H, 6-ArH), 7.15 (d, *J* = 8.4 Hz, 2H, 2′,6′-ArH), 6.83 (d, *J* = 8.6 Hz, 2H, 3′,5′-ArH), 6.47–6.42 (m, 2H, 3,5-ArH), 3.83 (s, 3H, 2-OCH_3_), 3.78 (s, 3H, 4′-OCH_3_), 3.24 (t, *J* = 7.7 Hz, 2H, ArCH_2_CH_2_-), 2.94 (t, *J* = 7.8 Hz, 2H, ArCH_2_CH_2_-). **ESI**-**MS** *m*/*z* 287.3 [M + H]^+^.

**1-(4-hydroxy-2-methoxyphenyl)ethan-1-one (10)**

Compound **3** (5 mmol) was dissolved in methanol (10 mL), followed by the addition of 4 N HCl (1 mL). The reaction mixture was stirred at room temperature for 12 h. After completion, the mixture was extracted with EA and water. The organic layer was dried over anhydrous Na_2_SO_4_, concentrated under reduced pressure, and recrystallized through ethanol to produce **10**.

Yield: 97.2%. White solid. **^1^H NMR** (400 MHz, CDCl_3_) δ 7.86 (d, *J* = 8.6 Hz, 1H, 6-ArH), 6.55 (dd, *J*_1_ = 8.6 Hz, *J*_2_ = 2.3 Hz, 1H, 5-ArH), 6.52 (d, *J* = 2.2 Hz, 1H, 3-ArH), 3.89 (s, 3H, -OCH_3_), 2.60 (s, 3H, -COCH_3_). **ESI**-**MS** *m*/*z* 167.3 [M + H]^+^.

#### 3.2.3. General Procedure for the Preparation of **5a**-**f**, **8a**-**f** and **11a**-**b**

A solution of **4a**-**d**, **7a**-**c**, or **10** (1.5 mmol), corresponding halo alcohol (1.95 mmol, 1.3 eq.), K_2_CO_3_ (4.5 mmol, 3 eq.), and NaI (0.15 mmol, 0.1 eq.) in DMF (3 mL) was refluxed overnight. After the reaction, the reacted mixture was extracted with EA and washed with brine. Then, it was dried over anhydrous Na_2_SO_4_ and concentrated via vacuum distillation to obtain compounds **5a**-**f**, **8a**-**f**, and **11a**-**b**. Since the solvent was difficult to remove completely and did not affect subsequent reactions, the crude products were directly used as raw materials for the next step.

**2-hydroxy-4-(2-hydroxyethoxy)benzoic acid (14)**

Commercially available 2,4-dihydroxybenzoic acid (**13**, 3.08 g, 20 mmol) was dissolved in ethanol (20 mL) and water (10 mL) with the addition of NaOH (50 mmol, 2.5 eq.) and NaI (2 mmol, 0.1 eq.). The solution was stirred in a water bath, followed by the addition of bromoethanol (24 mmol, 1.2 eq.). Then, the solution was heated to 60 °C and stirred overnight. The reaction was terminated by adding 1 N HCl until the pH was below 3. After removing part of the solvent via vacuum distillation, the mixture was filtered, extracted, washed with brine, concentrated, and further purified via column chromatography (PE: EA: AcOH = 2:1:0.01) to give **14**.

Yield: 75.4%. White solid. **^1^H NMR** (400 MHz, DMSO-*d*_6_) δ 11.49 (bs, 1H, -COOH), 7.69 (d, *J* = 8.8 Hz, 1H, 6-ArH), 6.50 (dd, *J*_1_ = 8.7 Hz, *J*_2_ = 2.4 Hz, 1H, 5-ArH), 6.47 (d, *J* = 2.4 Hz, 1H, 3-ArH), 4.03 (t, *J* = 4.9 Hz, 2H, HOCH_2_CH_2_O-), 3.70 (t, *J* = 4.9 Hz, 2H, HOCH_2_CH_2_O-). **ESI**-**MS** *m*/*z* 199.4 [M + H]^+^.

#### 3.2.4. General Procedure for the Preparation of **15a**-**f**

Compound **14** (800 mg, 4 mmol), NHS (4 mmol, 1 eq.) and DCC (4 mmol, 1 eq.) in 1,4-dioxane (10 mL) were stirred under N_2_ atmosphere overnight at ambient temperature. The reaction was filtered and transferred to another flask, with the addition of corresponding benzylamine (4 mmol, 1 eq.), NaHCO_3_ (4 mmol, 1 eq.), and water (4 mL). The solution was heated to 60 ℃ and stirred for 5 h monitored via TLC. The solution was extracted with EA and water, washed with brine, dried over anhydrous Na_2_SO_4_, concentrated by vacuum distillation, and purified through column chromatography to get mediates **15a**-**f** with a yield of 41%~52%.

***N*-benzyl-2-hydroxy-4-(2-hydroxyethoxy)benzamide (15a)**

Yield: 46%. White solid. **^1^H NMR** (400 MHz, CDCl_3_) δ 12.66 (s, 1H), 7.41–7.29 (m, 5H), 7.26 (d, *J* = 8.6 Hz, 1H), 6.48 (d, *J* = 2.5 Hz, 1H), 6.41 (dd, *J*_1_ = 8.8 Hz, *J*_2_ = 2.6 Hz, 1H), 6.39 (br, 1H), 4.62 (d, *J* = 5.6 Hz, 2H), 4.10 (t, *J* = 4.6 Hz, 2H), 3.98 (t, *J* = 4.6 Hz, 2H). **ESI**-**MS** *m*/*z* 288.0 [M + H]^+^.

***N*-(4-fluorobenzyl)-2-hydroxy-4-(2-hydroxyethoxy)benzamide (15b)**

Yield: 45%. White solid. **^1^H NMR** (400 MHz, CDCl_3_) δ 7.83 (d, *J* = 8.6 Hz, 2H), 7.59 (d, *J* = 8.6 Hz, 2H), 7.09 (d, *J* = 8.6 Hz, 2H), 6.52–6.41 (m, 2H), 6.39 (br, 1H), 4.60 (d, *J* = 5.5 Hz, 2H), 4.56 (t, *J* = 4.0 Hz, 2H), 4.18 (t, *J* = 4.0 Hz, 2H). **ESI**-**MS** *m*/*z* 306.1 [M + H]^+^.

**tert-butyl(4-((2-hydroxy-4-(2-hydroxyethoxy)benzamido)methyl) phenyl) carbamate (15c)**

Yield: 48%. Yellow solid. **^1^H NMR** (400 MHz, CDCl_3_) δ 7.34 (d, *J* = 8.1 Hz, 2H), 7.23 (d, *J* = 8.3 Hz, 2H), 6.54–6.34 (m, 4H), 4.54 (d, *J* = 5.4 Hz, 2H), 4.07 (t, *J* = 4.7 Hz, 2H), 3.96 (t, *J* = 4.5 Hz, 2H), 1.51 (s, 9H). **ESI**-**MS** *m*/*z* 403.1 [M + H]^+^.

**2-hydroxy-4-(2-hydroxyethoxy)-N-(4-(trifluoromethyl)benzyl)benzamide (15d)**

Yield: 41%. White solid. **^1^H NMR** (400 MHz, CDCl_3_) δ 8.21 (d, *J* = 8.8 Hz, 1H), 7.59 (d, *J* = 8.3 Hz, 2H), 7.46 (d, *J* = 8.2 Hz, 2H), 6.62 (dd, *J*_1_ = 8.8 Hz, *J*_2_ = 2.3 Hz, 1H), 6.54 (d, *J* = 2.3 Hz, 1H), 4.72 (d, *J* = 5.9 Hz, 2H), 4.14 (t, *J* = 4.3 Hz, 1H), 3.99 (t, *J* = 4.3 Hz, 1H), 3.92 (s, 3H). **ESI**-**MS** *m*/*z* 356.1 [M + H]^+^.

***N*-(4-cyanobenzyl)-2-hydroxy-4-(2-hydroxyethoxy)benzamide (15e)**

Yield: 52%. White solid. **^1^H NMR** (400 MHz, CDCl_3_) δ 8.26 (d, *J* = 8.5 Hz, 1H), 7.62 (d, *J* = 8.3 Hz, 2H), 7.48 (d, *J* = 8.2 Hz, 2H), 6.62 (dd, *J*_1_ = 8.7 Hz, *J*_2_ = 2.3 Hz, 1H), 6.54 (d, *J* = 2.3 Hz, 1H), 4.72 (d, *J* = 5.9 Hz, 2H), 4.14 (t, *J* = 4.1 Hz, 1H), 3.99 (t, *J* = 4.0 Hz, 1H), 3.92 (s, 3H). **ESI**-**MS** *m*/*z* 311.0 [M − H]^−^.

#### 3.2.5. General Procedure for the Preparation of **17a**-**b** and **17d**-**e**

To a solution of **15a**-**b** and **15d**-**e** (2 mmol) in DMF (10 mL), K_2_CO_3_ (10 mmol, 5 eq.) was added. Then CH_3_I solution (0.3 mL, 2 eq.) was added dropwise, and the reaction mixture was kept stirring at room temperature for 1.5 h, monitored by TLC. After cooling to room temperature, the mixture was filtered to remove solids, extracted with EA, dried over anhydrous Na_2_SO_4_, and concentrated by vacuum distillation to obtain crude products **17a**-**b** and **17d**-**e**. Since the solvent was difficult to remove completely and did not affect subsequent reactions, the crude products were used directly in the next step.

#### 3.2.6. General Procedure for the Preparation of **6a**-**f**, **9a**-**f**, **12a**-**b**, **16a**-**f**, **18a**-**b** and **18d**-**e**

A mixture of **5a**-**f**, **8a**-**f**, **11a**-**b**, **15a**-**f**, **17a**-**b,** and **17d**-**e** (1.05 mmol, 1 eq.) and benzosulfonyl furazan nitrogen oxide (1.35 mmol, 1.3 eq.) was sealed in a 50 mL flask in an ice water bath. Anhydrous DCM (10 mL) was added, and subsequently, DBU (2.1 mmol, 2 eq.) was added dropwise. After another 15 min, the solution was cooled to room temperature, stirred for 16–20 h and monitored via TLC. The solution was extracted with DCM, washed with brine, dried with Na_2_SO_4_, concentrated in a vacuum, and purified through column chromatography to obtain the target products **6a**-**f**, **9a**-**f**, **12a**-**b**, **16a**-**b**, **16d**-**f**, **18a**-**b**, and **18d**-**e**. **16c’** was subsequently deprotected in TFA for 2 h to yield target compound **16c**.

**4-(3-(4-cinnamoyl-3-methoxyphenoxy)propoxy)-3-(phenylsulfonyl)-1,2,5-oxadiazole 2-oxide (6a)**

Yield: 25%. White solid. mp 146−148 °C. **^1^H NMR** (400 MHz, CDCl_3_) δ 8.02–7.95 (m, 2H, 2″,6″-ArH), 7.78 (d, *J* = 8.6 Hz, 1H, 6-ArH), 7.75–7.70 (m, 1H, 4″-ArH), 7.69 (d, *J* = 15.8 Hz, 1H, ArCH=CH-), 7.62–7.58 (m, 2H, 3″,5″-ArH), 7.57–7.52 (m, 2H, 2′,6′-ArH), 7.53 (d, *J* = 16.0 Hz, 1H, ArCH=CH-), 7.45–7.36 (m, 3H, 3′,4′,5′-ArH), 6.60 (dd, *J*_1_ = 8.6 Hz, *J*_2_ = 2.2 Hz, 1H, 5-ArH), 6.55 (d, *J* = 2.2 Hz, 1H, 3-ArH), 4.66 (t, *J* = 6.0 Hz, 2H, -OCH_2_CH_2_CH_2_O-), 4.27 (t, *J* = 5.8 Hz, 2H, -OCH_2_CH_2_CH_2_O-), 3.91 (s, 3H, -OCH_3_), 2.40 (p, *J* = 5.9 Hz, 2H, -OCH_2_CH_2_CH_2_O-). **^13^C NMR** (151 MHz, CDCl_3_) δ 189.51, 162.09, 159.43, 157.86, 141.21, 136.88, 134.68, 134.40, 131.92, 129.01, 128.66, 127.83, 127.46, 127.31, 126.07, 121.55, 109.46, 104.58, 98.13, 66.86, 62.72, 54.82, 27.35. **HRMS(ESI)** *m*/*z*: calcd for C_27_H_24_N_2_O_8_S^+^ [M + H]^+^ 537.1326, found 537.1325.

**(*E*)-4-(3-(4-(3-(4-fluorophenyl)acryloyl)-3-methoxyphenoxy)propoxy)-3-(phenylsulfonyl)-1,2,5-oxadiazole 2-oxide (6b)**

Yield: 17%. White solid. mp 144−146 °C. **^1^H NMR** (400 MHz, CDCl_3_) δ 8.03–7.96 (m, 2H, 2″,6″-ArH), 7.79 (d, *J* = 8.5 Hz, 1H, 6-ArH), 7.73 (t, *J* = 7.0 Hz, 1H, 4″-ArH), 7.66 (d, *J* = 15.8 Hz, 1H, ArCH=CH-), 7.62–7.51 (m, 4H, 2′,6′,3″,5″ -ArH), 7.46 (d, *J* = 15.8 Hz, 1H, ArCH=CH-), 7.09 (t, *J* = 8.7 Hz, 2H, 3’,5’-ArH), 6.61 (dd, *J*_1_ = 8.6 Hz, *J*_2_ = 2.2 Hz, 1H, 5-ArH), 6.55 (d, *J* = 2.2 Hz, 1H, 3-ArH), 4.67 (t, *J* = 6.0 Hz, 2H, -OCH_2_CH_2_CH_2_O-), 4.27 (t, *J* = 5.8 Hz, 2H, -OCH_2_CH_2_CH_2_O-), 3.92 (s, 3H, -OCH_3_), 2.41 (p, *J* = 5.9 Hz, 2H, -OCH_2_CH_2_CH_2_O-). **^13^C NMR** (151 MHz, CDCl_3_) δ 189.22, 162.14, 161.90, 159.42, 157.85, 139.84, 136.86, 134.67, 131.94, 130.64, 130.61, 129.13, 129.08, 128.64, 127.45, 125.79, 125.77, 121.43, 115.02, 114.87, 109.45, 104.62, 98.11, 66.85, 62.74, 54.82, 27.33. **HRMS(ESI)** *m*/*z*: calcd for C_27_H_23_FN_2_O_8_S^+^ [M + H]^+^ 555.1232, found 555.1227.

**(*E*)-4-(2-(3-methoxy-4-(3-(4-methoxyphenyl)acryloyl)phenoxy)ethoxy)-3-(phenylsulfonyl)-1,2,5-oxadiazole 2-oxide (6c)**

Yield: 37%. White solid. mp 153−155 °C. **^1^H NMR** (400 MHz, CDCl_3_) δ 8.01 (d, *J* = 8.6 Hz, 2H, 2″,6″-ArH), 7.76 (d, *J* = 8.5 Hz, 1H, 6-ArH), 7.74–7.69 (m, 1H, 4″-ArH), 7.66 (d, *J* = 15.7 Hz, 1H, ArCH=CH-), 7.59–7.50 (m, 4H, 2′,6′,3″,5″ -ArH), 7.37 (d, *J* = 15.8 Hz, 1H, ArCH=CH-), 6.92 (d, *J* = 8.9 Hz, 2H, 3′,5′-ArH), 6.58 (dd, *J*_1_ = 8.5 Hz, *J*_2_ = 2.2 Hz, 1H, 5-ArH), 6.56 (d, *J* = 2.0 Hz, 1H, 3-ArH), 4.80 (t, *J* = 4.3 Hz, 2H, -OCH_2_CH_2_O-), 4.46 (t, *J* = 4.5 Hz, 2H, -OCH_2_CH_2_O-), 3.92 (s, 3H, 2-OCH_3_), 3.85 (s, 3H, 4′-OCH_3_). **^13^C NMR** (151 MHz, CDCl_3_) δ 190.74, 162.35, 161.35, 160.22, 158.77, 142.52, 137.97, 135.67, 132.75, 130.07, 129.67, 128.57, 128.03, 124.83, 123.30, 114.33, 110.42, 105.68, 99.34, 69.32, 65.46, 55.88, 55.40. **HRMS(ESI)** *m*/*z*: calcd for C_27_H_24_N_2_O_9_S^+^ [M + H]^+^ 553.1275, found 553.1270.

**(*E*)-4-(3-(3-methoxy-4-(3-(4-methoxyphenyl)acryloyl)phenoxy)propoxy)-3-(phenylsulfonyl)-1,2,5-oxadiazole 2-oxide (6d)**

Yield: 33%. White solid. mp 125−127 °C. **^1^H NMR** (400 MHz, CDCl_3_) δ 7.98 (d, *J* = 8.6 Hz, 2H, 2″,6″-ArH), 7.75 (d, *J* = 8.6 Hz, 1H, 6-ArH), 7.74–7.69 (m, 1H, 4″-ArH), 7.66 (d, *J* = 15.7 Hz, 1H, ArCH=CH-), 7.59–7.49 (m, 4H, 2′,6′,3″,5″ -ArH), 7.39 (d, *J* = 15.8 Hz, 1H, ArCH=CH-), 6.92 (d, *J* = 8.6 Hz, 2H, 3′,5′-ArH), 6.59 (dd, *J*_1_ = 8.6 Hz, *J*_2_ = 2.2 Hz, 1H, 5-ArH), 6.54 (d, *J* = 2.1 Hz, 1H, 3-ArH), 4.66 (t, *J* = 6.0 Hz, 2H, -OCH_2_CH_2_CH_2_O-), 4.26 (t, *J* = 5.8 Hz, 2H, -OCH_2_CH_2_CH_2_O-), 3.90 (s, 3H, 2-OCH_3_), 3.85 (s, 3H, 4’-OCH_3_), 2.40 (p, *J* = 5.9 Hz, 2H, -OCH_2_CH_2_CH_2_O-). **^13^C NMR** (151 MHz, CDCl_3_) δ 190.70, 162.87, 161.30, 160.27, 158.89, 142.31, 137.90, 135.71, 132.77, 130.04, 129.68, 128.47, 128.09, 124.91, 122.83, 114.31, 110.48, 105.50, 99.17, 67.89, 63.71, 55.83, 55.40, 28.37. **HRMS(ESI)** *m*/*z*: calcd for C_28_H_26_N_2_O_9_S^+^ [M + H]^+^ 567.1432, found 567.1431.

**(*E*)-4-(2-(4-(3-(4-cyanophenyl)acryloyl)-3-methoxyphenoxy)ethoxy)-3-(phenylsulfonyl)-1,2,5-oxadiazole 2-oxide (6e)**

Yield: 33%. Light yellow solid. mp 97−99 °C. **^1^H NMR** (400 MHz, CDCl_3_) δ 7.98 (d, *J* = 8.6 Hz, 2H, 2’’,6’’-ArH), 7.75 (d, *J* = 8.6 Hz, 1H, 6-ArH), 7.74–7.69 (m, 1H, 4″-ArH), 7.66 (d, *J* = 15.7 Hz, 1H, ArCH=CH-), 7.59–7.49 (m, 4H, 2′,6′,3″,5″ -ArH), 7.39 (d, *J* = 15.8 Hz, 1H, ArCH=CH-), 6.92 (d, *J* = 8.6 Hz, 2H, 3′,5′-ArH), 6.59 (dd, *J*_1_ = 8.6 Hz, *J*_2_ = 2.2 Hz, 1H, 5-ArH), 6.54 (d, *J* = 2.1 Hz, 1H, 3-ArH), 4.66 (t, *J* = 6.0 Hz, 2H, -OCH_2_CH_2_CH_2_O-), 4.26 (t, *J* = 5.8 Hz, 2H, -OCH_2_CH_2_CH_2_O-), 3.90 (s, 3H, 2-OCH_3_), 3.85 (s, 3H, 4′-OCH_3_), 2.40 (p, *J* = 5.9 Hz, 2H, -OCH_2_CH_2_CH_2_O-). **^13^C NMR** (151 MHz, CDCl_3_) δ 190.70, 162.87, 161.30, 160.27, 158.89, 142.31, 137.90, 135.71, 132.77, 130.04, 129.68, 128.47, 128.09, 124.91, 122.83, 114.31, 110.48, 105.50, 99.17, 67.89, 63.71, 55.83, 55.40, 28.37. **HRMS(ESI)** *m*/*z*: calcd for C_27_H_21_N_3_O_8_S^+^ [M + H]^+^ 570.0942, found 570.0941.

**(*E*)-4-(3-(4-(3-(4-cyanophenyl)acryloyl)-3-methoxyphenoxy)propoxy)-3-(phenylsulfonyl)-1,2,5-oxadiazole 2-oxide (6f)**

Yield: 28%. Light yellow solid. mp 92−94 °C. **^1^H NMR** (600 MHz, CDCl_3_) δ 8.02–7.97 (m, 2H, 2″,6″-ArH), 7.81 (t, *J* = 8.6 Hz, 1H, 6-ArH), 7.73 (t, *J* = 7.5 Hz, 1H, 4″-ArH), 7.67 (m, 4H, 2′,6′,3″,5’’-ArH), 7.65 (d, *J* = 15.8 Hz, 1H, ArCH=CH-), 7.61 (d, *J* = 15.8 Hz, 1H, ArCH=CH-), 7.55 (t, *J* = 8.1 Hz, 2H, 3’,5’-ArH), 6.61 (dd, *J*_1_ = 8.7 Hz, *J*_2_ = 2.2 Hz, 1H, 5-ArH), 6.55 (d, *J* = 2.2 Hz, 1H, 3-ArH), 4.66 (t, *J* = 6.0 Hz, 2H, -OCH_2_CH_2_CH_2_O-), 4.28 (t, *J* = 5.8 Hz, 2H, -OCH_2_CH_2_CH_2_O-), 3.93 (s, 3H, -OCH_3_), 2.40 (p, *J* = 5.9 Hz, 2H, -OCH_2_CH_2_CH_2_O-). **^13^C NMR** (151 MHz, CDCl_3_) δ 188.41, 162.65, 159.72, 157.86, 138.95, 138.09, 136.90, 134.67, 132.25, 131.58, 129.19, 128.65, 127.53, 127.48, 120.94, 117.55, 111.88, 109.47, 104.95, 98.11, 66.87, 62.88, 54.88, 27.36. **HRMS(ESI)** *m*/*z*: calcd for C_28_H_23_N_3_O_8_S^+^ [M + H]^+^ 562.1279, found 562.1283.

**4-(2-(3-methoxy-4-(3-phenylpropanoyl)phenoxy)ethoxy)-3-(phenylsulfonyl)-1,2,5-oxadiazole 2-oxide (9a)**

Yield: 26%. White solid. mp 120−122 °C. **^1^H NMR** (400 MHz, CDCl_3_) δ 8.01 (d, *J* = 8.6 Hz, 2H, 2″,6″-ArH), 7.85 (d, *J* = 8.7 Hz, 1H, 6-ArH), 7.71 (t, *J* = 7.5 Hz, 1H, 4″-ArH), 7.53 (t, *J* = 7.6 Hz, 2H, 3″,5″-ArH), 7.34–7.16 (m, 5H, -ArH), 6.56 (dd, *J*_1_ = 8.7 Hz, *J*_2_ = 2.3 Hz, 1H, 5-ArH), 6.52 (d, *J* = 2.4 Hz, 1H, 3-ArH), 4.79 (t, *J* = 4.5 Hz, 2H, -OCH_2_CH_2_O-), 4.44 (t, *J* = 4.4 Hz, 2H, -OCH_2_CH_2_O-), 3.89 (s, 3H, -OCH_3_), 3.30 (t, *J* = 7.6 Hz, 2H, ArCH_2_CH_2_-), 3.02 (t, *J* = 8.0 Hz, 2H, ArCH_2_CH_2_-). **^13^C NMR** (151 MHz, CDCl_3_) δ 199.46, 162.81, 160.78, 158.75, 141.90, 137.95, 135.66, 132.87, 129.65, 128.57, 128.48, 128.40, 125.88, 121.84, 110.42, 105.68, 99.02, 69.26, 65.42, 55.61, 45.39, 30.60. **HRMS(ESI)** *m*/*z*: calcd for C_26_H_24_N_2_O_8_S^+^ [M + H]^+^ 525.1326, found 525.1327.

**4-(3-(3-methoxy-4-(3-phenylpropanoyl)phenoxy)propoxy)-3-(phenylsulfonyl)-1,2,5-oxadiazole 2-oxide (9b)**

Yield: 25%. White solid. mp 110−112 °C. **^1^H NMR** (400 MHz, DMSO-*d*_6_) δ 8.01 (d, *J* = 7.8 Hz, 2H), 7.83 (d, *J* = 8.7 Hz, 1H), 7.71 (t, *J* = 7.6 Hz, 1H), 7.54 (t, *J* = 7.9 Hz, 2H), 7.32–7.22 (m, 4H), 7.18 (t, *J* = 7.0 Hz, 1H), 6.68 (d, *J* = 2.2 Hz, 1H, 3-ArH), 6.64 (dd, *J*_1_ = 8.7 Hz, *J*_2_ = 2.3 Hz, 1H, 5-ArH), 4.59 (t, *J* = 6.0 Hz, 2H), 4.21 (t, *J* = 6.1 Hz, 2H), 3.89 (s, 3H), 3.21 (t, *J* = 7.6 Hz, 2H), 2.90 (t, *J* = 7.6 Hz, 2H), 2.28 (p, *J* = 6.1 Hz, 2H). **^13^C NMR** (151 MHz, DMSO-*d*_6_) δ 198.78, 163.71, 161.03, 159.35, 142.11, 137.66, 136.64, 132.45, 130.49, 128.85, 128.83, 128.79, 126.27, 120.92, 111.05, 106.65, 99.58, 68.77, 64.69, 56.39, 45.22, 30.55, 28.29. **HRMS(ESI)** *m*/*z*: calcd for C_27_H_26_N_2_O_8_S^+^ [M + H]^+^ 539.1483, found 539.1483.

**4-(2-(4-(3-(4-fluorophenyl)propanoyl)-3-methoxyphenoxy)ethoxy)-3-(phenylsulfonyl)-1,2,5-oxadiazole 2-oxide (9c)**

Yield: 16%. White solid. mp 125−127 °C. **^1^H NMR** (400 MHz, CDCl_3_) δ 8.00 (d, *J* = 7.8 Hz, 2H, 2″,6″-ArH), 7.83 (d, *J* = 8.7 Hz, 1H, 6-ArH), 7.71 (t, *J* = 7.6 Hz, 1H, 4″-ArH), 7.54 (t, *J* = 7.9 Hz, 2H, 3″,5″-ArH), 7.19 (dd, *J*_1_ = 8.5 Hz, *J*_2_ = 5.5 Hz, 2H, 2’,6’-ArH), 6.96 (t, *J* = 8.7 Hz, 2H, 3’,5’-ArH), 6.56 (dd, *J*_1_ = 8.7 Hz, *J*_2_ = 2.3 Hz, 1H, 5-ArH), 6.51 (d, *J* = 2.3 Hz, 1H, 3-ArH), 4.79 (t, *J* = 4.3 Hz, 2H, -OCH_2_CH_2_O-), 4.44 (t, *J* = 4.7 Hz, 2H, -OCH_2_CH_2_O-), 3.90 (s, 3H, -OCH_3_), 3.26 (t, *J* = 7.6 Hz, 2H, ArCH_2_CH_2_-), 2.98 (t, *J* = 7.6 Hz, 2H, ArCH_2_CH_2_-). **^13^C NMR** (151 MHz, CDCl_3_) δ 199.19, 162.88, 160.79, 158.76, 137.95, 137.49, 137.47, 135.66, 132.86, 129.85, 129.80, 129.65, 128.58, 121.76, 115.16, 115.02, 110.43, 105.73, 99.04, 69.25, 65.44, 55.62, 45.42, 29.72. **HRMS(ESI)** *m*/*z*: calcd for C_26_H_23_FN_2_O_8_S^+^ [M + H]^+^ 543.1232, found 543.1230.

**4-(3-(4-(3-(4-fluorophenyl)propanoyl)-3-methoxyphenoxy)propoxy)-3-(phenylsulfonyl)-1,2,5-oxadiazole 2-oxide (9d)**

Yield: 23%. White solid. mp 103−105 °C. **^1^H NMR** (400 MHz, CDCl_3_) δ 8.01 (d, *J* = 7.2 Hz, 2H, 2″,6″-ArH), 7.84 (d, *J* = 8.7 Hz, 1H, 6-ArH), 7.72 (t, *J* = 7.6 Hz, 1H, 4″-ArH), 7.54 (t, *J* = 7.9 Hz, 2H, 3″,5″-ArH), 7.19 (dd, *J*_1_ = 8.5 Hz, *J*_2_ = 5.5 Hz, 2H, 2′,6′-ArH), 6.97 (t, *J* = 8.7 Hz, 2H, 3′,5′-ArH), 6.57 (dd, *J*_1_ = 8.7 Hz, *J*_2_ = 2.3 Hz, 1H, 5-ArH), 6.52 (d, *J* = 2.3 Hz, 1H, 3-ArH), 4.80 (t, *J* = 4.4 Hz, 2H, -OCH_2_CH_2_CH_2_O-), 4.45 (t, *J* = 4.9, 4.4 Hz, 2H), 3.90 (s, 3H, -OCH_3_), 3.27 (t, *J* = 7.6 Hz, 2H, ArCH_2_CH_2_-), 2.99 (t, *J* = 7.6 Hz, 2H, ArCH_2_CH_2_-). **^13^C NMR** (151 MHz, CDCl_3_) δ 198.14, 162.35, 159.79, 157.84, 136.87, 134.66, 131.80, 128.83, 128.77, 128.64, 127.46, 120.25, 114.12, 113.98, 109.45, 104.53, 97.84, 66.86, 62.74, 54.54, 44.37, 28.72, 27.33. **HRMS(ESI)** *m*/*z*: calcd for C_27_H_25_FN_2_O_8_S^+^ [M + H]^+^ 557.1388, found 557.1389.

**4-(2-(3-methoxy-4-(3-(4-methoxyphenyl)propanoyl)phenoxy)ethoxy)-3-(phenylsulfonyl)-1,2,5-oxadiazole 2-oxide (9e)**

Yield: 32%. White solid. mp 91−93 °C. **^1^H NMR** (400 MHz, CDCl_3_) δ 8.01 (d, *J* = 7.6 Hz, 2H, 2″,6″-ArH), 7.83 (d, *J* = 8.6 Hz, 1H, 6-ArH), 7.71 (t, *J* = 7.5 Hz, 1H, 4″-ArH), 7.53 (t, *J* = 7.5 Hz, 2H, 3″,5″-ArH), 7.16 (d, *J* = 8.5 Hz, 2H, 2’,6’-ArH), 6.83 (d, *J* = 8.6 Hz, 2H, 3’,5’-ArH), 6.56 (dd, *J*_1_ = 8.7 Hz, *J*_2_ = 2.2 Hz, 1H, 5-ArH), 6.51 (d, *J* = 2.3 Hz, 1H, 3-ArH), 4.79 (t, *J* = 4.6 Hz, 2H, -OCH_2_CH_2_O-), 4.44 (t, *J* = 4.5 Hz, 2H, -OCH_2_CH_2_O-), 3.89 (s, 3H, 2-OCH_3_), 3.79 (s, 3H, 4’-OCH_3_), 3.25 (t, *J* = 7.8 Hz, 2H, ArCH_2_CH_2_-), 2.95 (t, *J* = 7.8 Hz, 2H, ArCH_2_CH_2_-). **^13^C NMR** (151 MHz, CDCl_3_) δ 199.61, 162.78, 160.75, 158.75, 157.80, 137.95, 135.65, 133.93, 132.84, 129.65, 129.37, 128.57, 121.90, 113.80, 110.42, 105.67, 99.01, 69.26, 65.42, 55.61, 55.27, 45.67, 29.71. **HRMS(ESI)** *m*/*z*: calcd for C_27_H_26_N_2_O_9_S^+^ [M + H]^+^ 555.1432, found 555.1426.

**4-(3-(3-methoxy-4-(3-(4-methoxyphenyl)propanoyl)phenoxy)propoxy)-3-(phenylsulfonyl)-1,2,5-oxadiazole 2-oxide (9f)**

Yield: 18%. White solid. mp 100−102 °C. **^1^H NMR** (600 MHz, CDCl_3_) δ 7.98 (d, *J* = 8.4 Hz, 2H, 2″,6″-ArH), 7.82 (d, *J* = 8.7 Hz, 1H, 6-ArH), 7.71 (t, *J* = 7.5 Hz, 1H, 4″-ArH), 7.53 (t, *J* = 7.7 Hz, 2H, 3″,5″-ArH), 7.15 (d, *J* = 8.5 Hz, 2H, 2′,6′-ArH), 6.83 (d, *J* = 8.6 Hz, 2H, 3′,5′-ArH), 6.55 (dd, *J*_1_ = 8.7 Hz, *J*_2_ = 2.2 Hz, 1H, 5-ArH), 6.49 (d, *J* = 2.2 Hz, 1H, 3-ArH), 4.64 (t, *J* = 6.0 Hz, 2H, -OCH_2_CH_2_CH_2_O-), 4.24 (t, *J* = 5.8 Hz, 2H, -OCH_2_CH_2_CH_2_O-), 3.87 (s, 3H, 2-OCH_3_), 3.78 (s, 3H, 4′-OCH_3_), 3.24 (t, *J* = 8.0 Hz, 2H, ArCH_2_CH_2_-), 2.95 (t, *J* = 7.8 Hz, 2H, ArCH_2_CH_2_-), 2.38 (p, *J* = 5.9 Hz, 2H, -OCH_2_CH_2_CH_2_O-). **^13^C NMR** (151 MHz, CDCl_3_) δ 198.58, 162.26, 159.76, 157.85, 156.78, 136.92, 134.65, 132.98, 131.79, 128.64, 128.34, 127.45, 120.46, 112.78, 109.45, 104.50, 97.86, 66.87, 62.73, 54.54, 54.24, 44.62, 28.73, 27.35. **HRMS(ESI)** *m*/*z*: calcd for C_28_H_28_N_2_O_9_S^+^ [M + H]^+^ 569.1588, found 569.1585.

**4-(2-(4-acetyl-3-methoxyphenoxy)ethoxy)-3-(phenylsulfonyl)-1,2,5-oxadiazole 2-oxide (12a)**

Yield: 38%. White solid. mp 114−116 °C. **^1^H NMR** (400 MHz, CDCl_3_) δ 8.01 (d, *J* = 7.3 Hz, 2H, 2″,6″-ArH), 7.86 (d, *J* = 8.6 Hz, 1H, 6-ArH), 7.71 (t, *J* = 7.5 Hz, 1H, 4″-ArH), 7.54 (t, *J* = 8.1 Hz, 2H, 3″,5″-ArH), 6.55 (dd, *J*_1_ = 8.6 Hz, *J*_2_ = 2.3 Hz, 1H, 5-ArH), 6.52 (d, *J* = 2.2 Hz, 1H, 3-ArH), 4.79 (t, *J* = 4.5 Hz, 2H, -OCH_2_CH_2_O-), 4.44 (t, *J* = 4.5 Hz, 2H, -OCH_2_CH_2_O-), 3.92 (s, 3H, -OCH_3_), 2.59 (s, 3H, -COCH_3_). **^13^C NMR** (151 MHz, CDCl_3_) δ 196.75, 161.94, 160.09, 157.74, 136.97, 134.63, 131.77, 128.63, 127.56, 120.98, 109.41, 104.61, 98.02, 68.25, 64.43, 54.58, 54.52, 30.86. **HRMS(ESI)** *m*/*z*: calcd for C_19_H_18_N_2_O_8_S^+^ [M + H]^+^ 457.0676, found 457.0678.

**4-(3-(4-acetyl-3-methoxyphenoxy)propoxy)-3-(phenylsulfonyl)-1,2,5-oxadiazole 2-oxide (12b)**

Yield: 44%. White solid. mp 112−114 °C. **^1^H NMR** (400 MHz, CDCl_3_) δ 7.98 (d, *J* = 7.9 Hz, 2H, 2″,6″-ArH), 7.85 (d, *J* = 8.7 Hz, 1H, 6-ArH), 7.72 (t, *J* = 7.5 Hz, 1H, 4″-ArH), 7.53 (t, *J* = 8.0 Hz, 2H, 3″,5″-ArH), 6.55 (dd, *J*_1_ = 8.7 Hz, *J*_2_ = 2.3 Hz, 1H, 5-ArH), 6.50 (d, *J* = 2.2 Hz, 1H, 3-ArH), 4.64 (t, *J* = 6.0 Hz, 2H, -OCH_2_CH_2_CH_2_O-), 4.24 (t, *J* = 5.8 Hz, 2H, -OCH_2_CH_2_CH_2_O-), 3.90 (s, 3H, -OCH_3_), 2.58 (s, 3H, -COCH_3_), 2.38 (p, *J* = 5.9 Hz, 2H, -OCH_2_CH_2_CH_2_O-). **^13^C NMR** (151 MHz, CDCl_3_) δ 197.77, 163.47, 161.13, 158.88, 137.94, 135.68, 132.75, 129.66, 128.48, 121.54, 110.48, 105.46, 98.87, 67.90, 63.76, 55.55, 31.87, 28.37. **HRMS(ESI)** *m*/*z*: calcd for C_20_H_20_N_2_O_8_S^+^ [M + H]^+^ 471.0833, found 471.0837.

**4-(2-(4-(benzylcarbamoyl)-3-hydroxyphenoxy)ethoxy)-3-(phenylsulfonyl)-1,2,5-oxadiazole 2-oxide (16a)**

Yield: 24%. White solid. mp 119−121 °C. **^1^H NMR** (400 MHz, CDCl_3_) δ 8.01 (d, *J* = 7.7 Hz, 2H, 2″,6″-ArH), 7.71 (t, *J* = 7.4 Hz, 1H, 4″-ArH), 7.54 (t, *J* = 7.4 Hz, 2H, 3″,5″-ArH), 7.41–7.29 (m, 6H, ArH), 6.49 (d, *J* = 2.5 Hz, 1H, 3-ArH), 6.44–6.39 (m, 2H, 5-ArH, -CONH-), 4.76 (t, *J* = 4.4 Hz, 2H, -OCH_2_CH_2_O-), 4.64 (d, *J* = 5.5 Hz, 2H, ArCH_2_NH-), 4.38 (t, *J* = 4.4 Hz, 2H, -OCH_2_CH_2_O-). **^13^C NMR** (151 MHz, CDCl_3_) δ 168.53, 162.88, 161.83, 157.69, 137.03, 136.51, 134.63, 128.65, 127.92, 127.54, 126.94, 126.89, 125.87, 109.34, 106.92, 105.88, 101.63, 68.16, 64.26, 42.65. **HRMS(ESI)** *m*/*z*: calcd for C_24_H_21_N_3_O_8_S^+^ [M − H]^+^ 510.0977, found 510.0978.

**4-(2-(4-((4-fluorobenzyl)carbamoyl)-3-hydroxyphenoxy)ethoxy)-3-(phenylsulfonyl)-1,2,5-oxadiazole 2-oxide (16b)**

Yield: 22%. White solid. mp 119−121 °C. **^1^H NMR** (400 MHz, CDCl_3_) δ 8.01 (d, *J* = 7.8 Hz, 2H, 2″,6″-ArH), 7.73 (t, *J* = 7.6 Hz, 1H, 4″-ArH), 7.53 (t, *J* = 7.4 Hz, 2H, 3″,5″-ArH), 7.37–7.29 (m, 3H, 6,3’,5’-ArH), 7.05 (t, *J* = 8.6 Hz, 2H, 2’,6’-ArH), 6.49 (d, *J* = 1.9 Hz, 1H, 3-ArH), 6.47–6.41 (m, 2H, 5-ArH, -CONH-), 4.76 (t, *J* = 3.6 Hz, 2H, -OCH_2_CH_2_O-), 4.60 (d, *J* = 5.5 Hz, 2H, ArCH_2_NH-), 4.38 (d, *J* = 3.8 Hz, 2H, -OCH_2_CH_2_O-). **^13^C NMR** (151 MHz, CDCl_3_) δ 168.55, 162.87, 162.18, 161.89, 160.55, 157.68, 137.00, 134.64, 132.39, 132.37, 128.66, 128.61, 127.54, 125.89, 114.83, 114.69, 109.35, 106.83, 105.96, 101.65, 68.16, 64.28, 41.89. **HRMS(ESI)** *m*/*z*: calcd for C_24_H_20_FN_3_O_8_S^+^ [M + H]^+^ 530.1028, found 530.1029.

**4-(2-(4-((4-aminobenzyl)carbamoyl)-3-hydroxyphenoxy)ethoxy)-3-(phenylsulfonyl)-1,2,5-oxadiazole 2-oxide (16c)**

Yield: 29%. White solid. mp 150−152 °C. **^1^H NMR** (400 MHz, DMSO-*d*_6_) δ 13.18 (s, 1H, -ArOH), 9.06 (t, *J* = 5.8 Hz, 1H, -CONH-), 7.97 (d, *J* = 7.8 Hz, 2H, 2″,6″-ArH), 7.90–7.80 (m, 2H, 6,4″-ArH), 7.66 (t, *J* = 7.7 Hz, 2H, 3″,5″-ArH), 6.99 (d, *J* = 8.5 Hz, 2H, 2′,6′-ArH), 6.55–6.46 (m, 4H, 3,5,3′,5′-ArH), 4.99 (s, 2H, -NH_2_), 4.72 (t, *J* = 4.5 Hz, 2H, -OCH_2_CH_2_O-), 4.40 (d, *J* = 4.2 Hz, 2H, -OCH_2_CH_2_O-), 4.30 (d, *J* = 5.6 Hz, 2H, ArCH_2_NH-). **^13^C NMR** (151 MHz, DMSO-*d*_6_) δ 169.33, 163.12, 162.71, 159.25, 148.14, 137.67, 136.56, 130.40, 129.42, 128.89, 128.74, 126.32, 114.19, 111.01, 108.74, 106.74, 102.44, 70.23, 66.00, 42.45. **HRMS(ESI)** *m*/*z*: calcd for C_24_H_22_N_4_O_8_S^+^ [M + H]^+^ 527.1231, found 527.1232.

**4-(2-(3-hydroxy-4-((4-(trifluoromethyl)benzyl)carbamoyl)phenoxy)ethoxy)-3-(phenylsulfonyl)-1,2,5-oxadiazole 2-oxide (16d)**

Yield: 29%. White solid. mp 96−98 °C. **^1^H NMR** (400 MHz, DMSO-*d*_6_) δ 12.87 (s, 1H, -ArOH), 9.32 (t, *J* = 6.0 Hz, 1H, -CONH-), 7.98 (d, *J* = 7.8 Hz, 2H, 2″,6″-ArH), 7.92–7.80 (m, 2H, 6,4″-ArH), 7.74–7.62 (m, 4H, 3′,5′,3″,5″-ArH), 7.54 (d, *J* = 8.0 Hz, 2H, 2′,6′-ArH), 6.58 (dd, *J*_1_ = 8.7 Hz, *J*_2_ = 2.2 Hz, 1H, 5-ArH), 6.52 (d, *J* = 2.4 Hz, 1H, 3-ArH), 4.73 (t, *J* = 3.4 Hz, 2H, -OCH_2_CH_2_O-), 4.58 (d, *J* = 5.8 Hz, 2H, ArCH_2_NH-), 4.41 (t, *J* = 3.6 Hz, 2H, -OCH_2_CH_2_O-). **^13^C NMR** (151 MHz, DMSO-*d*_6_) δ 168.64, 161.89, 161.86, 158.20, 143.45, 136.61, 135.50, 129.34, 128.56, 127.69, 127.33, 124.71, 124.69, 124.66, 124.64, 109.95, 107.56, 105.91, 101.44, 69.15, 65.00, 41.36. **HRMS(ESI)** *m*/*z*: calcd for C_25_H_20_F_3_N_3_O_8_S^+^ [M − H]^+^ 578.0850, found 578.0865.

**4-(2-(4-((4-cyanobenzyl)carbamoyl)-3-hydroxyphenoxy)ethoxy)-3-(phenylsulfonyl)-1,2,5-oxadiazole 2-oxide (16e)**

Yield: 28%. Light yellow solid. mp 132−134 °C. **^1^H NMR** (600 MHz, DMSO-*d*_6_) δ 12.79 (s, 1H, -ArOH), 9.29 (t, *J* = 5.9 Hz, 1H, -CONH-), 7.97 (d, *J* = 8.0 Hz, 2H, 2″,6″-ArH), 7.87 (d, *J* = 8.9 Hz, 1H, 6-ArH), 7.84 (t, *J* = 7.4 Hz, 1H, 4″-ArH), 7.81 (d, *J* = 8.3 Hz, 2H, 3′,5′-ArH), 7.66 (t, *J* = 8.0 Hz, 2H, 3″,5″-ArH), 7.51 (d, *J* = 8.3 Hz, 2H, 2′,6′-ArH), 6.57 (dd, *J*_1_ = 8.9 Hz, *J*_2_ = 2.6 Hz, 1H, 5-ArH), 6.50 (d, *J* = 2.6 Hz, 1H, 3-ArH), 4.73 (t, *J* = 4.0 Hz, 2H, -OCH_2_CH_2_O-), 4.57 (d, *J* = 5.8 Hz, 2H, ArCH_2_NH-), 4.40 (t, *J* = 4.0 Hz, 2H, -OCH_2_CH_2_O-). **^13^C NMR** (151 MHz, DMSO-*d*_6_) δ 168.61, 161.85, 158.17, 144.45, 136.58, 135.49, 131.75, 129.33, 128.59, 127.67, 127.45, 118.25, 109.93, 109.05, 107.55, 105.92, 101.42, 69.13, 64.98, 41.47. **ESI**-**MS** *m*/*z* 537.3 [M + H]^+^.

**4-(2-(3-hydroxy-4-((4-hydroxybenzyl)carbamoyl)phenoxy)ethoxy)-3-(phenylsulfonyl)-1,2,5-oxadiazole 2-oxide (16f)**

Yield: 16%. White solid. mp 115−117 °C. **^1^H NMR** (400 MHz, DMSO-*d*_6_) δ 12.95 (s, 1H, 2-ArOH), 9.29 (s, 1H, 4′-ArOH), 8.03 (d, *J* = 7.8 Hz, 2H), 7.91–7.80 (m, 3H), 7.65 (t, *J* = 8.0 Hz, 2H), 7.43 (d, *J* = 7.9 Hz, 2H), 7.39 (d, *J* = 8.1 Hz, 2H), 6.58 (dd, *J* = 8.8, 2.5 Hz, 1H, 5-ArH), 6.51 (d, *J* = 2.5 Hz, 1H, 3-ArH), 4.72 (t, *J* = 4.5 Hz, 2H, -OCH_2_CH_2_O-), 4.52 (d, *J* = 5.1 Hz, 2H, ArCH_2_NH-), 4.40 (t, *J* = 4.5 Hz, 2H, -OCH_2_CH_2_O-). **^13^C NMR** (151 MHz, DMSO-*d*_6_) δ 164.05, 161.04, 158.19, 144.41, 136.59, 135.52, 131.90, 129.36, 126.69, 127.17, 123.55, 124.83, 124.51, 114.64, 109.96, 105.67, 98.44, 69.29, 65.11, 41.74. **HRMS(ESI)** *m*/*z*: calcd for C_24_H_21_N_3_O_9_S^+^ [M − H]^+^ 526.0926, found 526.0928.

**4-(2-(4-(benzylcarbamoyl)-3-methoxyphenoxy)ethoxy)-3-(phenylsulfonyl)-1,2,5-oxadiazole 2-oxide (18a)**

Yield: 17%. White solid. mp 134−136 °C. **^1^H NMR** (400 MHz, CDCl_3_) δ 8.27 (d, *J* = 8.6 Hz, 1H, 6-ArH), 8.11 (t, *J* = 5.0 Hz, 1H, -CONH-), 8.00 (d, *J* = 7.5 Hz, 2H, 2″,6″-ArH), 7.72 (t, *J* = 7.4 Hz, 1H, 4″-ArH), 7.52 (t, *J* = 7.6 Hz, 2H, 3″,5″-ArH), 7.38–7.27 (m, 5H, -ArH), 6.64 (dd, *J*_1_ = 8.8 Hz, *J*_2_ = 2.3 Hz, 1H, 5-ArH), 6.55 (d, *J* = 2.2 Hz, 1H, 3-ArH), 4.79 (d, *J* = 3.6 Hz, 2H, -OCH_2_CH_2_O-), 4.69 (d, *J* = 5.3 Hz, 2H, ArCH_2_NH-), 4.44 (t, *J* = 3.4 Hz, 2H, -OCH_2_CH_2_O-), 3.92 (s, 3H, -OCH_3_). **^13^C NMR** (151 MHz, CDCl_3_) δ 163.91, 160.88, 157.94, 157.74, 137.91, 136.96, 134.62, 133.24, 128.63, 127.63, 127.55, 126.50, 126.21, 114.28, 109.40, 105.02, 98.25, 68.29, 64.48, 55.07, 42.69. **HRMS(ESI)** *m*/*z*: calcd for C_25_H_23_N_3_O_8_S^+^ [M + H]^+^ 526.1279, found 526.1283.

**4-(2-(4-((4-fluorobenzyl)carbamoyl)-3-methoxyphenoxy)ethoxy)-3-(phenylsulfonyl)-1,2,5-oxadiazole 2-oxide (18b)**

Yield: 23%. White solid. mp 135−137 °C. **^1^H NMR** (400 MHz, CDCl_3_) δ 8.26 (d, *J* = 8.8 Hz, 1H, 6-ArH), 8.09 (t, *J* = 6.5 Hz, 1H, -CONH-), 8.00 (d, *J* = 7.8 Hz, 2H, 2″,6″-ArH), 7.70 (t, *J* = 7.5 Hz, 1H, 4″-ArH), 7.53 (t, *J* = 7.5 Hz, 2H, 3″,5″-ArH), 7.33 (t, *J* = 6.8 Hz, 2H, 3′,5′-ArH), 7.02 (t, *J* = 8.8 Hz, 2H, 2′,6′-ArH), 6.64 (dd, *J*_1_ = 8.9 Hz, *J*_2_ = 2.2 Hz, 1H, 5-ArH), 6.56 (d, *J* = 2.4 Hz, 1H, 3-ArH), 4.79 (t, *J* = 4.5 Hz, 2H, -OCH_2_CH_2_O-), 4.64 (d, *J* = 5.7 Hz, 2H, ArCH_2_NH-), 4.43 (t, *J* = 4.5 Hz, 2H, -OCH_2_CH_2_O-), 3.93 (s, 3H, -OCH_3_). **^13^C NMR** (151 MHz, CDCl_3_) δ 163.93, 161.85, 160.96, 160.22, 157.94, 157.75, 136.95, 134.62, 133.75, 133.73, 133.24, 128.63, 128.19, 128.14, 127.56, 114.49, 114.35, 114.16, 109.41, 105.07, 98.27, 68.29, 64.49, 55.08, 41.97. **HRMS(ESI)** *m*/*z*: calcd for C_25_H_22_FN_3_O_8_S^+^ [M + H]^+^ 544.1184, found 544.1189.

**4-(2-(3-methoxy-4-((4-(trifluoromethyl)benzyl)carbamoyl)phenoxy)ethoxy)-3-(phenylsulfonyl)-1,2,5-oxadiazole 2-oxide (18d)**

Yield: 25%. White solid. mp 148−150 °C. **^1^H NMR** (400 MHz, DMSO-*d*_6_) δ 8.72 (t, *J* = 6.1 Hz, 1H, -CONH-), 7.98 (d, *J* = 7.9 Hz, 2H, 2″,6″-ArH), 7.88–7.80 (m, 2H, 6,4″-ArH), 7.72–7.62 (m, 4H, 3′,5′,3″,5″-ArH), 7.53 (d, *J* = 7.9 Hz, 2H, 2′,6′-ArH), 6.72 (d, *J* = 2.3 Hz, 1H, 3-ArH), 6.70 (dd, *J*_1_ = 8.5 Hz, *J*_2_ = 2.3 Hz, 1H, 5-ArH), 4.76 (t, *J* = 4.3 Hz, 2H, -OCH_2_CH_2_O-), 4.58 (d, *J* = 6.1 Hz, 2H, ArCH_2_NH-), 4.46 (t, *J* = 4.1 Hz, 1H, -OCH_2_CH_2_O-), 3.94 (s, 3H, -OCH_3_). **^13^C NMR** (151 MHz, DMSO-*d*_6_) δ 164.05, 161.04, 158.21, 158.19, 144.41, 136.57, 135.52, 131.90, 129.36, 127.69, 127.10, 124.55, 124.53, 124.51, 114.64, 109.96, 105.67, 98.44, 69.29, 65.11, 55.53, 41.74. **HRMS(ESI)** *m*/*z*: calcd for C_26_H_22_F_3_N_3_O_8_S^+^ [M + H]^+^ 594.1152, found 594.1158.

**4-(2-(4-((4-cyanobenzyl)carbamoyl)-3-methoxyphenoxy)ethoxy)-3-(phenylsulfonyl)-1,2,5-oxadiazole 2-oxide (18e)**

Yield 15%. Light yellow solid. mp 116−118 °C. **^1^H NMR** (400 MHz, DMSO-*d*_6_) δ 8.72 (t, *J* = 6.1 Hz, 1H, -CONH-), 7.98 (d, *J* = 7.9 Hz, 2H, 2″,6″-ArH), 7.88–7.74 (m, 4H, 6,3′,5′,4″-ArH), 7.67 (t, *J* = 7.9 Hz, 2H, 3″,5″-ArH), 7.50 (d, *J* = 8.0 Hz, 2H, 2′,6′-ArH), 6.72 (d, *J* = 2.3 Hz, 1H, 3-ArH), 6.70 (dd, *J*_1_ = 8.6 Hz, *J*_2_ = 2.3 Hz, 1H, 5-ArH), 4.76 (t, *J* = 4.1 Hz, 2H, -OCH_2_CH_2_O-), 4.57 (d, *J* = 6.1 Hz, 2H, ArCH_2_NH-), 4.46 (t, *J* = 4.4 Hz, 2H, -OCH_2_CH_2_O-), 3.94 (s, 3H, -OCH_3_). **^13^C NMR** (151 MHz, DMSO-*d*_6_) δ 164.09, 161.07, 158.21, 145.47, 136.57, 135.52, 131.91, 131.62, 129.36, 127.69, 127.28, 118.37, 114.57, 109.96, 108.71, 105.68, 98.44, 69.28, 65.11, 55.53, 41.90. **HRMS(ESI)** *m*/*z*: calcd for C_26_H_22_N_4_O_8_S^+^ [M + H]^+^ 551.1231, found 551.1235.

### 3.3. Solubility Experiment

Wavelength: 254 nm; flow rate: 1.0 mL/min; column temperature: 25 °C. Mobile phase: Phase A: water, Phase B: acetonitrile (Table 5).

Compounds were dissolved in chromatographic-grade acetonitrile and diluted to concentrations of 200, 100, 50, 25, 12.5, and 6.25 µg/mL. A standard curve was plotted using the peak area under the curve (AUC) versus concentration. Then, 1 mg of each test compound was weighed and dissolved in 1 mL of a 1:1 (*v*/*v*) water–acetonitrile mixture, followed by ultrasonic shocking for 10 min. The supernatant was filtered through an ultrafiltration membrane and then subjected to HPLC analysis. The solubility of the target compound was determined by substituting the AUC of the test compound into the standard curve. Each measurement was performed in triplicate.

### 3.4. Biology

#### 3.4.1. Cell Lines and Culture

The MDA-MB-468 cell line was procured from the Cell Bank of the Chinese Academy of Sciences. The MCF-7, MCF-7/ADR, MCF-10A, and MDA-MB-231 cell lines were kindly provided by Professor Xianyi Sha (Fudan University, Shanghai, China). The HUVEC cells were kindly provided by Associate Professor Hulie Zeng (Fudan University, Shanghai, China). The MDA-MB-468, MDA-MB-231, MCF-7, MCF-10A, and HUVEC cells were cultivated in DMEM (Service Biotechnology, China) supplemented with 10% FBS (Capricorn Scientific, Germany) and 1% penicillin-streptomycin (Meilunbio, Dalian, China). For the MCF-7/ADR cells, they were cultured in RPMI-1640 (Service Biotechnology, Wuhan, China), containing 10% FBS and 1% penicillin-streptomycin. Additionally, 500 ng/mL of doxorubicin (Sigma-Aldrich, Saint Louis, MO, USA) was incorporated into the RPMI-1640 culture medium to keep drug resistance. All cell lines were cultured in a humidified incubator at 37 °C with a 5% CO_2_ atmosphere.

#### 3.4.2. In Vitro Antiproliferative Assay

Following harvest during logarithmic growth, cells were plated in 96-well culture plates at an initial density of 5000 cells per well. Incubate the plate overnight at 37 °C to allow the cells to adhere. Following the overnight incubation, introduce the compound at the predetermined concentration into each well, and then return the plate to the incubator for an additional 48 h culture period. After the 48 h treatment, carefully aspirate the drug-containing medium from each well. Subsequently, add 150 μL of 0.5 mg/mL MTT (Beyotime, China) solution to each well. Protect the plate from light and incubate it at 37 °C for 4 h to allow the formation of formazan crystals. Finally, add 150 μL of DMSO (Sinopharm, China) to each well and gently mix to ensure complete dissolution of the formazan crystals. The OD value at 570nm was measured using a microplate reader (Thermo Fisher, Waltham, MA, USA). Cell proliferation inhibition was determined using the following formula:

Inhibition (%) = [(OD_DMSO_ − OD_compd._)/(OD_DMSO_ − OD_blank_)] × 100

#### 3.4.3. IC_50_ Values Calculation

GraphPad Prism software 9 was used to calculate the IC_50_ values. The specific calculation process is as follows: Firstly, we input the experimental data into Prism. The data include the concentrations of the experimental compounds and the corresponding biological response values. For instance, in the cell experiment, we recorded the cell viability values at different drug concentrations. Subsequently, we select the option of “Dose–Response–Nonlinear Regression” in the “Analyze” menu for curve fitting. Generally speaking, we use the model of “[Inhibitor] vs. normalized response—Variable slope”. The Prism software fits the data through the nonlinear least squares method and finds the parameter values that can minimize the difference between the model’s predicted values and the actual data. After completing the curve fitting, the software will directly provide the estimated values and confidence intervals of the IC_50_ value and other relevant parameters. Meanwhile, it will also offer goodness-of-fit indices, such as the correlation coefficient, to evaluate the quality of the fitting. Through the above method, we can accurately calculate the IC_50_ value, thereby assessing the intensity of the inhibitory or activating effect of the compound on the biological system.

#### 3.4.4. Measurement of Intracellular NO

Harvest the MCF-7/ADR cells and seed them into 6 cm dishes (3 × 10^6^ cells/dish). After overnight incubation, indicated concentrations of **9e** were added to the cells for 24 h. The release of NO was measured using a total NO detection kit (Beyotime, Shanghai, China).

#### 3.4.5. Rh123 Accumulation

Harvest the MCF-7/ADR cells and seed them into a 96-well plate (2 × 10^5^ cells/well). Following overnight culture, cells were treated with **9e** (10 and 20 nM) or DMSO (vehicle control) for 60 min. After aspiration of the medium, cells were washed thrice with PBS and subsequently incubated with Rh123 solution (1 μg/mL; Sigma-Aldrich) for 60 min. Subsequently, wash three times with PBS, then digest and centrifuge them (1000× *g*, 5 min). Resuspend the cells with 1 mL of PBS and centrifuge again (1000× *g*, 5 min). Finally, after resuspending the cells with 200 μL of PBS, the fluorescence of Rh123 was analyzed by flow cytometry (Beckman, Brea, CA, USA).

#### 3.4.6. Measurement of Lysosomal and Mitochondrial NO Levels in Subcellular Organelles

Lysosomal NO probes (Lyso-NO) and mitochondrial NO probes (Mito-NO), which are specifically used for detecting the nitric oxide levels in lysosomes and mitochondria, respectively, were provided by Professor Weili Zhao (Fudan University, China). Cells (MCF-7 or MCF-7/ADR) were plated in 6-well plates (2 × 10^5^ cells/well). After overnight incubation, indicated concentrations of **9e** were added to the cells for 4 h. Cells were first treated with 10 μM Lyso-NO or Mito-NO (15 min, 37 °C), then counterstained with Hoechst 33,342 nuclear dye (Beyotime, China; 10 min, 37 °C). Finally, the fluorescence intensity was measured under a fluorescence microscope (Olympus, Tokyo, Japan). 

#### 3.4.7. Cell Apoptosis Analysis

MCF-7/ADR (2 × 10^5^ cells/well) were plated in 6-well plates and cultured overnight. Following 24 h treatment with specified concentrations of **9e**, cells were harvested and analyzed by Annexin V-FITC apoptosis detection kit (Beyotime, Shanghai, China). Cell populations were subsequently analyzed by flow cytometry (Beckman, Brea, CA, USA).

#### 3.4.8. Treatment of Cells with Inhibitors

Carboxy-PTIO (NO scavenger), NAC (*N*-acetylcysteine), tariquidar (P-glycoprotein inhibitor), and Fer-1 (ferrostatin-1, ferroptosis inhibitor) were purchased from Beyotime. MCF-7/ADR (5000 cells/well) were plated in 96-well plates and cultured overnight. Then, the cells were pre-treated with specified concentrations of inhibitors for 1 h and incubated with **9e** (10 or 20 nM) or DMSO (24 h, 37 °C). Experimental procedures were the same as the method for the in vitro antiproliferative assay.

#### 3.4.9. Western Blot

MCF-7/ADR (2 × 10^5^ cells/well) were plated in 6-well plates. After 24 h treatment with **9e** (20 nM), cell lysates were prepared using RIPA buffer (Beyotime, Shanghai, China; 30 min, ice), then centrifuged (12,000 rpm, 10 min, 4 °C) to harvest protein supernatants. Protein concentrations were measured by BCA assay (Beyotime, Shanghai, China). The supernatants were then loaded and transferred onto PVDF membranes (Millipore, Billerica, MA, USA) for western blot. The blots were developed with ECL chemiluminescent solution (Meilunbio, Dalian, China). Commercially purchased antibodies include P-gp (Abcam, Cambridge, UK; ab170904), Caspase 3 (Proteintech, Wuhan, China; 19677-1-AP), Bcl-2 (Servicebio, Wuhan, China; GB113375-100), LC3 (Abcam, Abcam, Cambridge, UK; ab192890), P62 (Cell Signaling Technology, Danvers, MA, USA; 5114 T), Beclin (ABclonal, Wuhan, China; A7353), GPX4 (ABclonal, Wuhan, China; A11243), LAMP1 (Cell Signaling Technology, Danvers, MA, USA;15,665 T), GAPDH (Servicebio, Wuhan, China; GB11002-100), and β-Actin (Servicebio, Wuhan, China;GB11001-100). The secondary antibodies conjugated with horseradish peroxidase (HRP) were from Abmart.

#### 3.4.10. Immunofluorescence

MCF-7/ADR (1 × 10^5^ cells/dish) were seeded in confocal dishes and cultured overnight. After being treated with **9e** (20 nM) for 24 h, cells were fixed with 4% paraformaldehyde (Servicebio, Wuhan, China) for 15 min at room temperature. Then, they were washed three times with PBS. Next, cells were blocked with a solution containing 3% BSA (Biofroxx, Einhausen, Germany) and 0.3% Triton X-100 (Beyotime, Shanghai, China) for 1 h at room temperature. The cells were incubated with the primary antibody, which was diluted in 3% BSA and 0.3% Triton X-100, overnight at 4 °C. After three washes with PBS, the cells were incubated with the fluorescent secondary antibody (Alexa Fluor 488 and Alexa Fluor 594, Bioss, Beijing, China) for 1 h at room temperature. Following another three washes with PBS, the cellular DNA was stained with Hoechst 33,342 dye (10 min, 37 °C).

#### 3.4.11. Inhibition on hERG Potassium Channels

CHO-hERG cells (2 × 10^6^ cells/dish) were seeded in confocal dishes. The compound stock solution was diluted with DMSO to six concentrations: 40.00, 13.33, 4.44, 1.48, 0.49, and 0.16 μM, which was prepared by using the Bravo platform. Both single-cell high-resistance sealing and whole-cell mode formation were automatically performed by the QPatch system (Shanghai Institute of Materia Medica, Shanghai, China). Each concentration was tested on at least two cells (n ≥ 2).

#### 3.4.12. Statistical Analysis

All the experiments in vitro were conducted in triplicate, and the data were presented as the mean ± SD. For comparing the results between two groups, unpaired two-tailed Student’s *t* tests were employed. One-way ANOVA tests were used to test the statistical significance among multiple comparisons. Significance was established at *p* < 0.05 (* *p* < 0.05, ** *p* < 0.01, *** *p* < 0.001, ns *p* > 0.05). All statistical tests were performed with GraphPad Prism 9.

## 4. Conclusions

In summary, by opening the lactone ring of the coumarin skeleton and introducing different side chains, twenty-four seco-coumarin/furoxan hybrids (**6a**-**f**, **9a**-**f**, **12a**-**b**, **16a**-**f**, **18a**-**b**, and **18d**-**e**) were designed and synthesized. All compounds showed remarkable cytotoxicity against drug-resistant MCF-7/ADR cell lines, with an IC_50_ of less than 100 nM. Most of them retained collateral sensitivity, among which four compounds (**9a**-**b** and **9d**-**e**) reached over 1000-fold selectivity between MCF-7/ADR and MCF-7. The SAR indicated that the coumarin core was not the key fragment responsible for maintaining their antiproliferative activity. Seco-coumarin/furoxan derivatives with *α*, *β*-unsaturated ketone or *α*, *β*-saturated ketone side chains showed better activity compared to those with an amide linker, among compounds with *α*, *β*-saturated side chains that showed stronger collateral sensitivity. In particular, **9e** containing 4-methoxyphenyl on its *α*, *β*-saturated side chain exhibited excellent proliferation inhibitory activity (IC_50(MCF-7/ADR)_ = 3.02 nM) and collateral sensitivity (IC_50(MCF-7)/_IC_50(MCF-7/ADR)_ = 1402), as well as high safety against normal cell lines such as MCF-10A.

Furthermore, the preliminary study of pharmacologic mechanism revealed that most of the synthesized compounds were capable of producing an effective NO concentration, and the antiproliferative activity of the compound **9e** was clearly decreased after being treated with the NO scavenger. The accumulation Rh123 assay suggested that **9e** might be a potential substrate for P-gp, exploiting the transporters’ overexpression in MCF-7/ADR cells to accumulate in lysosomes, where subsequent NO release triggered ROS generation and apoptotic cell death. Meanwhile, significant changes in NO concentration were rarely observed in the mitochondria of MCF-7/ADR and lysosomes or the mitochondria of MCF-7. The evidence indicated that the high collateral sensitivity of compound **9e** against MCF-7/ADR likely results from its lysosomal P-gp hijacking mechanism. However, the fact that the P-gp inhibitor tariquidar did not cause **9e** to totally lose its cytotoxicity indicated the existence of other possible pharmacological mechanisms. The experimental results of autophagy flow and the expression-downregulation of GPX4 suggested that the intense antiproliferative action of compound **9e** might also be related to autophagy and ferroptosis pathways. In conclusion, compound **9e** showed higher collateral sensitivity and improved water solubility than the lead compound **4A93**, indecating it as a good candidate for further research to overcome drug resistance in the context of cancer, possibly through multiple pathways.

## Figures and Tables

**Table 1:** The antiproliferative effects of synthesized compounds against four breast cancer cell lines.

Compounds	IC_50_ (nM) ^a^				Selectivity Ratio
	MCF-7	MCF-7/ADR	MDA-MB-231	MDA-MB-468	IC_50(MCF-7)_/ IC_50(MCF-7/ADR)_
**6a**	472.8 ± 5.4%	1.1 ± 10.8%	2823 ± 5.1%	299.9 ± 9.4%	432.6
**6b**	771 ± 8.1%	2.7 ± 2.7%	1686 ± 11.4%	82.2 ± 4.9%	286.6
**6c**	549.1 ± 8.6%	12.4 ± 6.0%	2504 ± 3.8%	118.3 ± 8.2%	44.2
**6d**	531 ± 8.9%	5.8 ± 3.7%	2676 ± 11.5%	128.2 ± 5.9%	91.1
**6e**	3077 ± 3.8%	9.4 ± 2.4%	4004 ± 9.1%	133.9 ± 6.3%	328.3
**6f**	1671 ± 7.1%	7.9 ± 10.8%	4108 ± 4.6%	83.0 ± 7.0%	212.9
**9a**	4457 ± 3.2%	2.6 ± 5.4%	3084 ± 8.7%	258.8 ± 11.2%	1716.9
**9b**	2984 ± 2.9%	2.6 ± 6.8%	1905 ± 9.5%	311.4 ± 4.1%	1152.6
**9c**	1332 ± 10.3%	9.7 ± 7.6%	2781 ± 3.9%	267.9 ± 5.0%	137.1
**9d**	2200 ± 8.4%	1.6 ± 2.1%	1736 ± 6.5%	333.3 ± 9.8%	1394.2
**9e**	4227 ± 4.3%	3.0 ± 7.9%	2133 ± 2.3%	53.0 ± 3.8%	1402.0
**9f**	2226 ± 5.1%	2.4 ± 6.7%	4415 ± 8.4%	62.2 ± 9.9%	943.2
**12a**	4825 ± 11.2%	25.5 ± 4.5%	3649 ± 7.0%	509.8 ± 10.6%	189.3
**12b**	3442 ± 2.9%	52.5 ± 5.4%	3233 ± 7.8%	769.0 ± 9.3%	65.6
**16a**	642.9 ± 3.2%	89.2 ± 6.1%	1744 ± 8.5%	56.8 ± 10.2%	7.2
**16b**	2534 ± 2.7%	91.1 ± 4.9%	2597 ± 6.3%	384.3 ± 9.1%	27.8
**16c**	6597 ± 3.6%	69.1 ± 5.8%	3122 ± 7.4%	165.1 ± 10.9%	95.4
**16d**	2715 ± 4.2%	10.5 ± 7.8%	1895 ± 11.2%	43.5 ± 3.6%	257.8
**16e**	3014 ± 9.5%	5.4 ± 2.7%	6192 ± 6.1%	88.4 ± 4.3%	561.9
**16f**	2055 ± 10.4%	31.9 ± 5.9%	2910 ± 8.2%	106.4 ± 3.1%	64.4
**18a**	1704 ± 7.4%	35.7 ± 4.8%	1102 ± 6.7%	68.0 ± 2.3%	47.8
**18b**	3827 ± 9.1%	37.7 ± 5.2%	1017 ± 8.6%	230.3 ± 3.9%	101.7
**18d**	1064 ± 7.0%	6.5 ± 4.5%	2547 ± 6.4%	44.8 ± 2.9%	163.0
**18e**	4669 ± 8.9%	19.9 ± 5.6%	3064 ± 9.8%	88.0 ± 5.4%	235.0
doxorubicin	1024 ± 3.6%	131,619 ± 5.4%	/	/	0.008
**4A93**	2495 ± 6.4%	5.0 ± 6.3%	670.2 ± 8.5%	8.9 ± 4.5%	499.0

^a^ The data are the mean of three independent determinations.

**Table 2:** The antiproliferative effects of selected compounds against a normal mammary epithelial cell lines.

**Compd.**	**6a**	**6c**	**6d**	**6e**	**9a**	**9e**
**IC_50_ (μM) ^a^**	3.0 ± 5.3%	8.4 ± 3.8%	13.4 ± 9.2%	1.8 ± 6.9%	3.0 ± 2.4%	13.2 ± 7.6%
**Compd.**	**12a**	**16d**	**16e**	**18d**	**18e**	**4A93**
**IC_50_ (μM) ^a^**	5.9 ± 10%	3.8 ± 4.7%	3.2 ± 8.3%	2.2 ± 11.7%	5.2 ± 3.5%	23.2 ± 7.2%

^a^ The data are the mean of three independent determinations.

**Table 3:** The solubility of target compounds (in water/acetonitrile = 2:1).

Compounds	Solubility (μg/mL)	Solubility_Target Compounds_/ Solubility _4A93_	Compounds	Solubility (μg/mL)	Solubility_Target Compounds_/ Solubility _4A93_
**4A93**	0.8 ± 9.1%	1	**12a**	67.9 ± 7.8%	85
**6a**	60.8 ± 7.2%	76	**12b**	384 ± 9.3%	480
**6b**	45.8 ± 3.4%	57	**16a**	125 ± 7.1%	156
**6c**	37.7 ± 5.2%	47	**16b**	31.6 ± 6.8%	40
**6d**	24.3 ± 2.5%	30	**16c**	327 ± 11.6%	409
**6e**	21.2 ± 3.3%	27	**16d**	61.1 ± 2.6%	76
**6f**	67.2 ± 6.1%	84	**16e**	33.8 ± 5.3%	42
**9a**	23.5 ± 4.1%	29	**16f**	81.0 ± 7.9%	101
**9b**	19.4 ± 3.1%	24	**18a**	70.3 ± 6.1%	88
**9c**	40.8 ± 8.2%	51	**18b**	60.9 ± 9.8%	76
**9d**	117 ± 10.8%	146	**18d**	26.2 ± 7.2%	33
**9e**	56.4 ± 5.5%	71	**18e**	44.7 ± 10.2%	56
**9f**	125 ± 9.2%	156			

The data are the mean of three independent determinations.

**Table 4:** Inhibition of hERG potassium channels.

Compd.	Structure	IC_50_ (μM)
**9e**	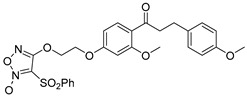	>40
**Cisapride**	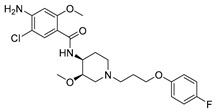	0.047

**Table 5:** Gradient elution mode for HPLC.

Time (min)	Phase A	Phase B
0	70	30
18	2	98
25	2	98

## Data Availability

Data are contained within the article and Supplementary Materials.

## Supplementary Materials

The following supporting information can be downloaded at: https://www.mdpi.com/article/10.3390/molecules30112341/s1. Figure S1: Antiproliferative activity of selected compounds in HUVEC cell lines; Figure S2: Antiproliferative activity of compound 9e(CY-21S-2A80) against four breast cancer cell lines; Figure S3: Inhibitory effects of compound 9e(CY-21S-2A80) on hERG potassium currents. ^1^H NMR, ^13^C NMR and HRMS Spectra of compound **6a**-**f**, **9a**-**f**, **12a**-**b**, **16a**-**f**, **18a**-**b**, and **18d**-**e**.

## Abbreviations

ABC	ATP binding cassette transporter
ABCC1	MDR-associated protein 1
ABCG2/BCRP	Breast cancer resistance protein
ADC	Antibody–drug conjugate
BC	Breast cancer
CDK4/6	Cyclin-dependent kinase 4/6
DBU	1,8-diazabicyclo [5.4.0]-7-undecene
DCC	*N*, *N*-dicyclohexylcarbodiimide
DHP	3,4-dihydro-2*H*-pyran
DMF	*N*, *N*-dimethylformamide
DOX	Doxorubicin
ET	Endocrine treatment
hERG	Human ether-à-go-go related gene
HER2	Human epidermal growth factor receptor 2
LC3	Microtubule-associated protein light chain 3
MDR	Multidrug resistance
mTOR	Mechanistic target of rapamycin
MTT	Thiazolyl blue tetrazolium bromide
NAC	*N*-acetyl-*L*-cysteine
NHS	*N*-hydroxysuccinimide
NO	Nitric oxide
NOS	Nitric oxide synthase
OD	Optical density
PARP	Poly ADP-ribose polymerase
PE	Petroleum ether
P-gp	P-glycoprotein
PI3K	Phosphatidylinositol 3-kinase
PPTS	Pyridinium toluene-4-Sulphonate
ROS	Reactive oxygen species
