# Supplementary material for: Synthesis and Biological Evaluation of Seco-Coumarin/Furoxan Hybrids as Potent Anti-Tumor Agents to Overcome Multidrug Resistance via Multiple Mechanisms
Source: Molecules. 2025 May 27;30(11):2341. doi: 10.3390/molecules30112341 (PMC12156367; doi:10.3390/molecules30112341)
Supplement: Supplementary file 1 [file molecules-30-02341-s001.zip › molecules-3633210-supplementary.pdf]

Synthesis and Biological Evaluation of Seco-Coumarin/Furoxan Hybrids as Potent Antitumor Agent to Overcoming Multidrug Resistance via Multiple Mechanisms

Feng Qu [a,1], Xiufan Wu [a,1], Jiachen Weng [b,1], Shuquan Zhang [a], La Li [a], Xuqin Guo [a], Hongrui Liu [b,*], Ying Chen [a,*]

[a] *Department of Medicinal Chemistry, School of Pharmacy, Fudan University, Shanghai 201203, China*

[b] *Department of Pharmacology, School of Pharmacy, Fudan University, Shanghai 201203, China*

** Corresponding authors.*

E-mail addresses: liuhr@fudan.edu.cn (H. R. Liu), yingchen71@fudan.edu.cn (Y. Chen).

[1] *Feng Qu, Xiufan Wu and Jiachen Weng are co-first authors and contribute equally to this work.*

Table of Contents

1) Antiproliferative activity of compounds in HUVEC cell lines
2) Antiproliferative activity of compound 9e against four breast cancer cell lines.
3) Inhibitory effects of compounds on hERG potassium currents
4) ^{1}H NMR, ^{13}C NMR and HRMS Spectra

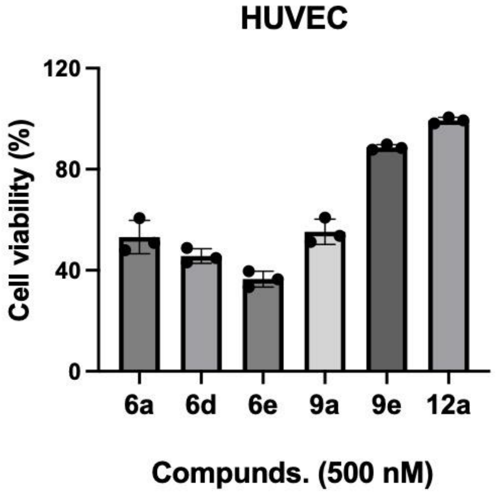

Figure S1. Antiproliferative activity of selected compounds in HUVEC cell lines. The data are the mean of triplicate determinations.

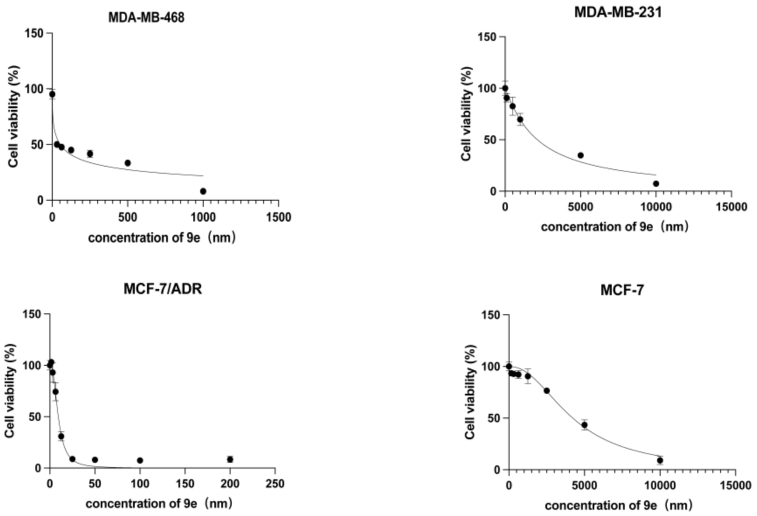

Figure S2. Antiproliferative activity of compound **9e**(**CY-21S-2A80**) against four breast cancer cell lines. The data are the mean of triplicate determinations.

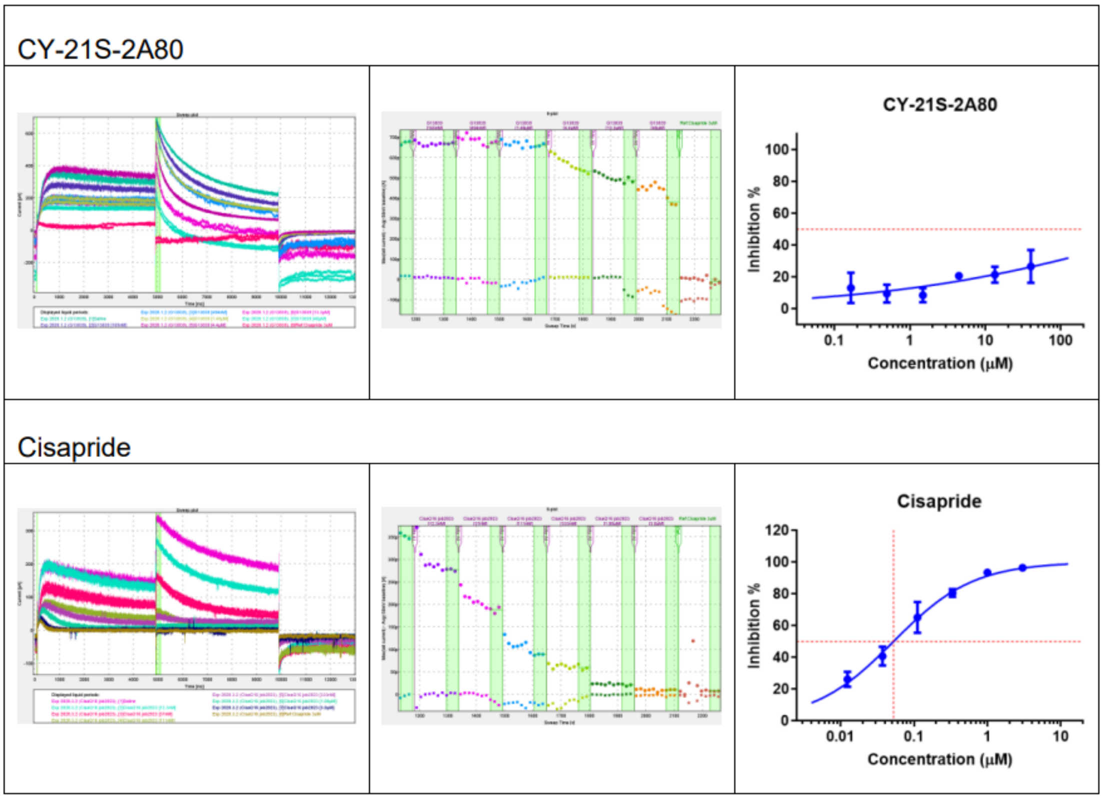

Figure S3. Inhibitory effects of compound **9e**(**CY-21S-2A80**) on hERG potassium currents.(left) Current traces showing the effect of the compound on hERG channels; (Center) Time-course plot; (Right) Dose-response curve.

^{1}H NMR, ^{13}C NMR and HRMS Spectra of Compound 6a

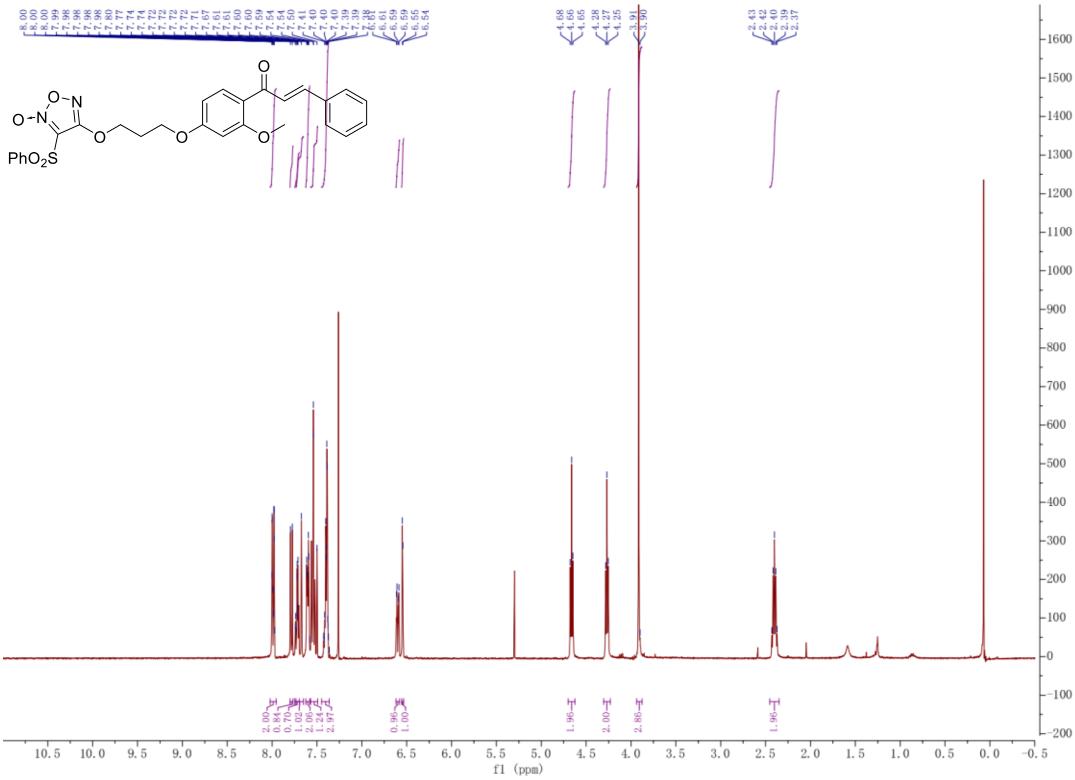

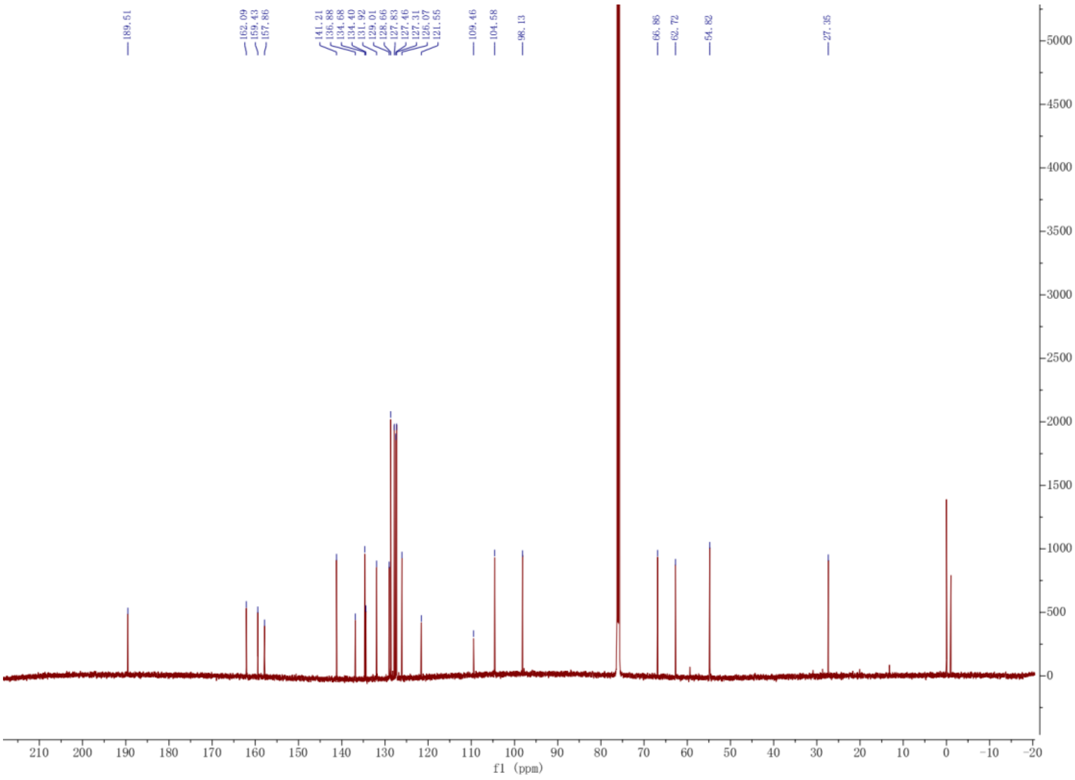

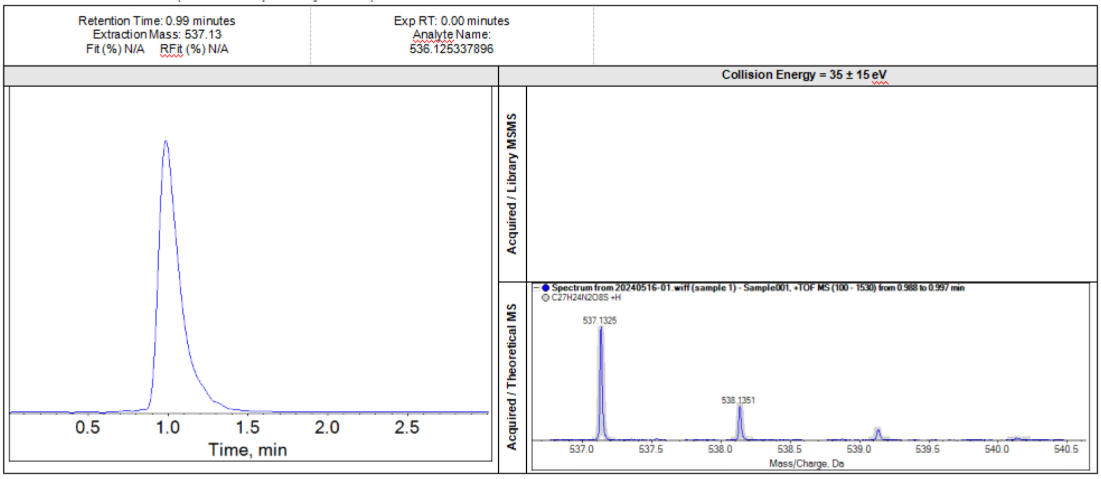

	Compound Name (Library Hit)	Score	Formula	Intensity	Threshold	Expected m/z	Found at m/z	Error (ppm)	Expected RT (min)	Found RT (min)	RT Delta (min)	Isotope Diff (%)	Library Score (%)
	536.125337896 (No Acquired MSMS)	98%	C27H24N2O8S	161462	5	537.1326	537.1325	-0.3	0.00	0.99	0.99	2.3%	N/A

^{1}H NMR, ^{13}C NMR and HRMS Spectra of Compound 6b

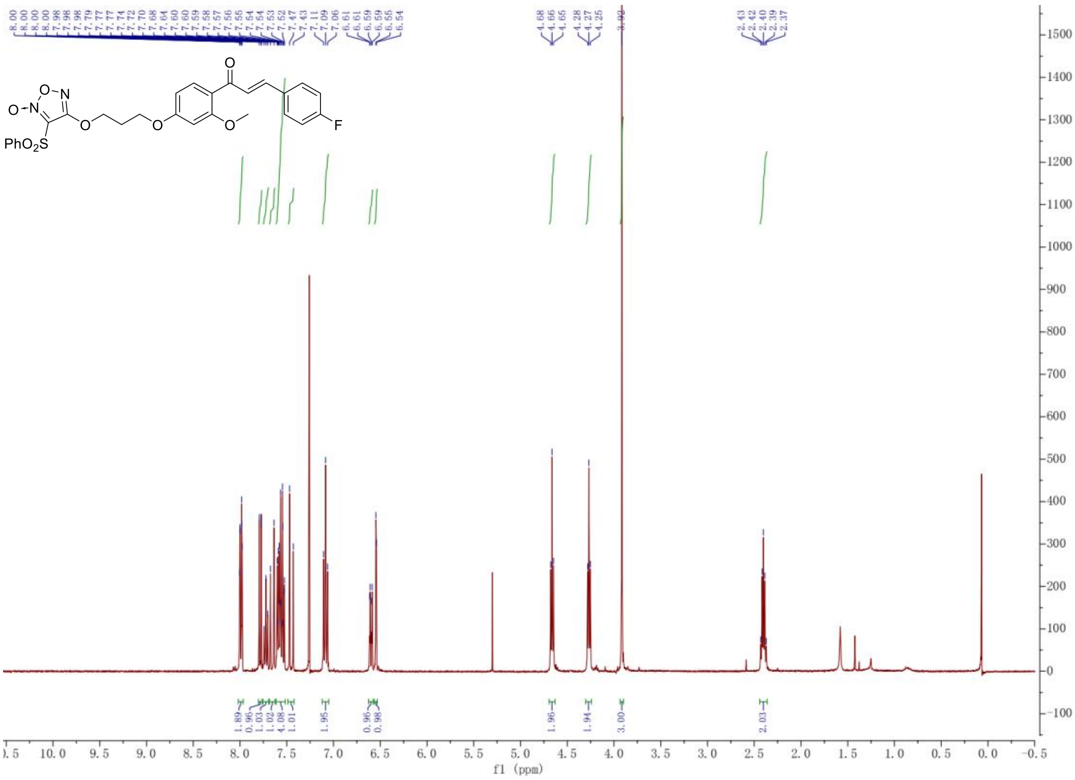

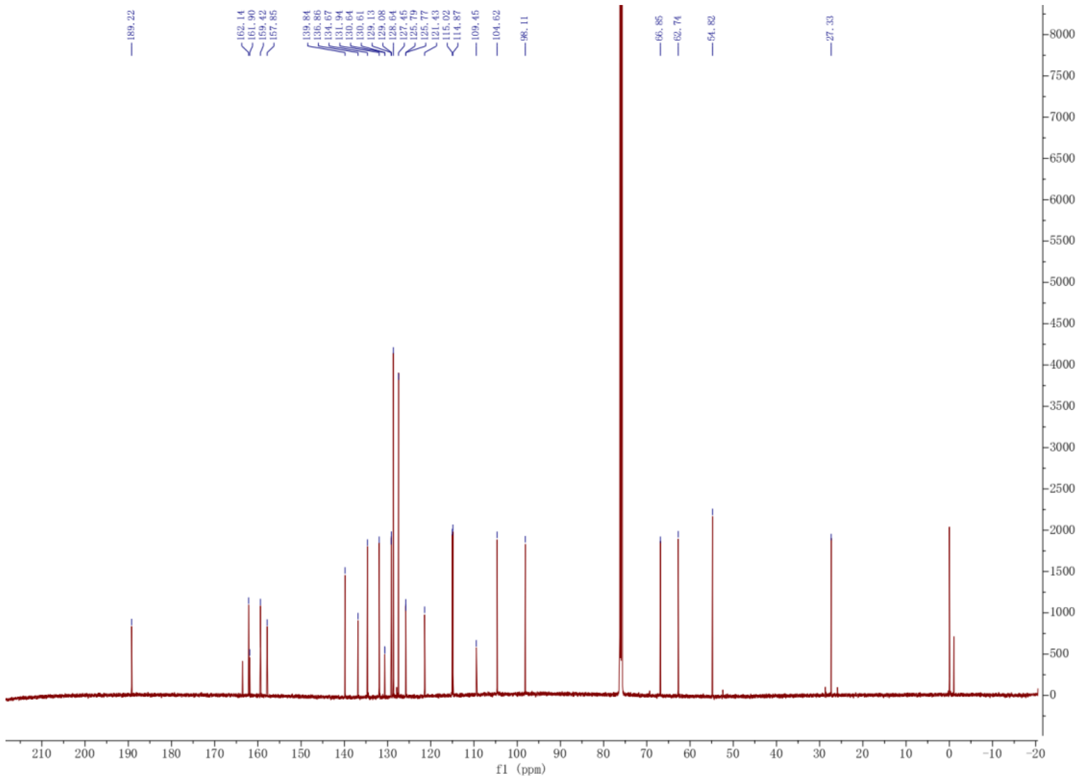

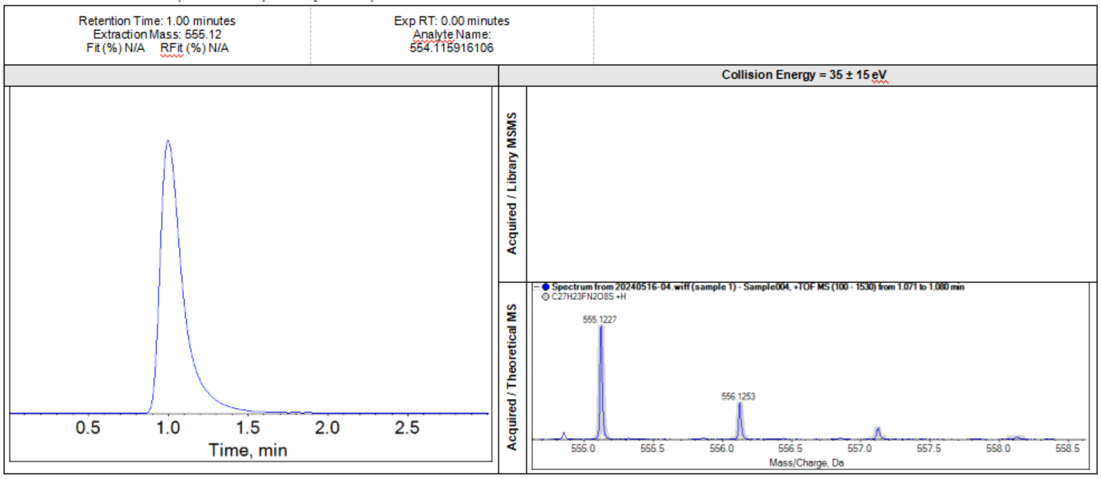

	Compound Name (Library Hit)	Score	Formula	Intensity	Threshold	Expected m/z	Found at m/z	Error (ppm)	Expected RT (min)	Found RT (min)	RT Delta (min)	Isotope Diff (%)	Library Score (%)
	554.115916106 (No Acquired MSMS)	97%	C27H23FN2O8S	274872	5	555.1232	555.1227	-0.9	0.00	1.00	1.00	1.2%	N/A

^{1}H NMR, ^{13}C NMR and HRMS Spectra of Compound 6c

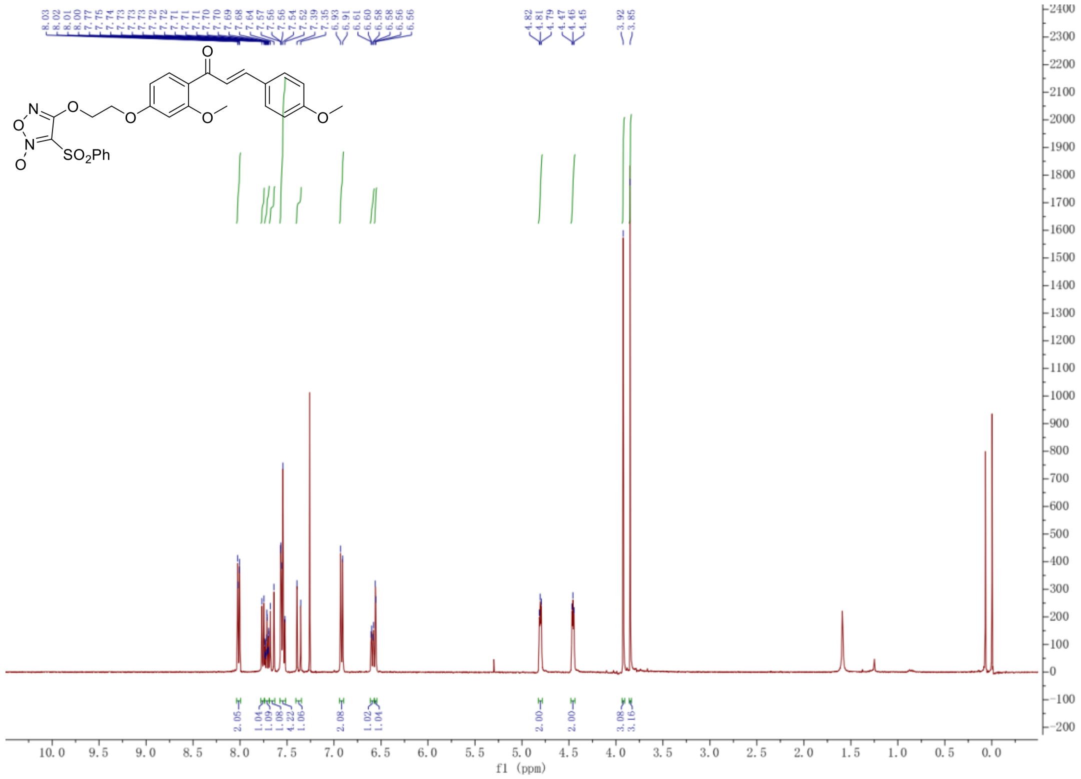

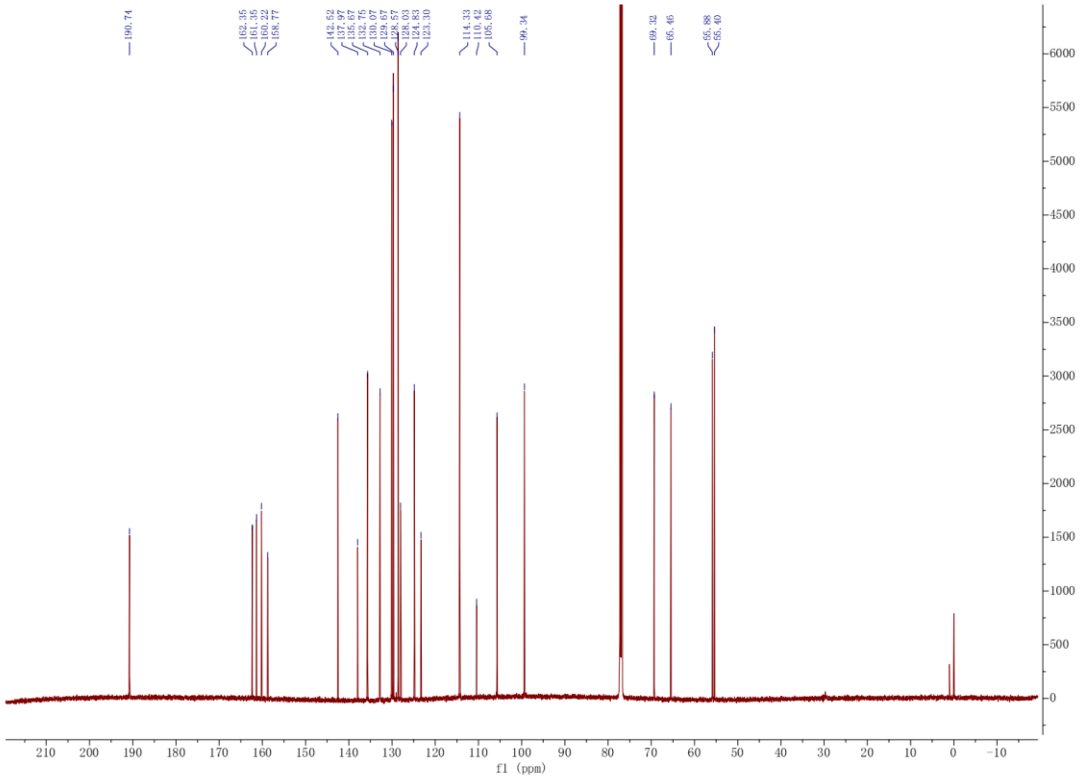

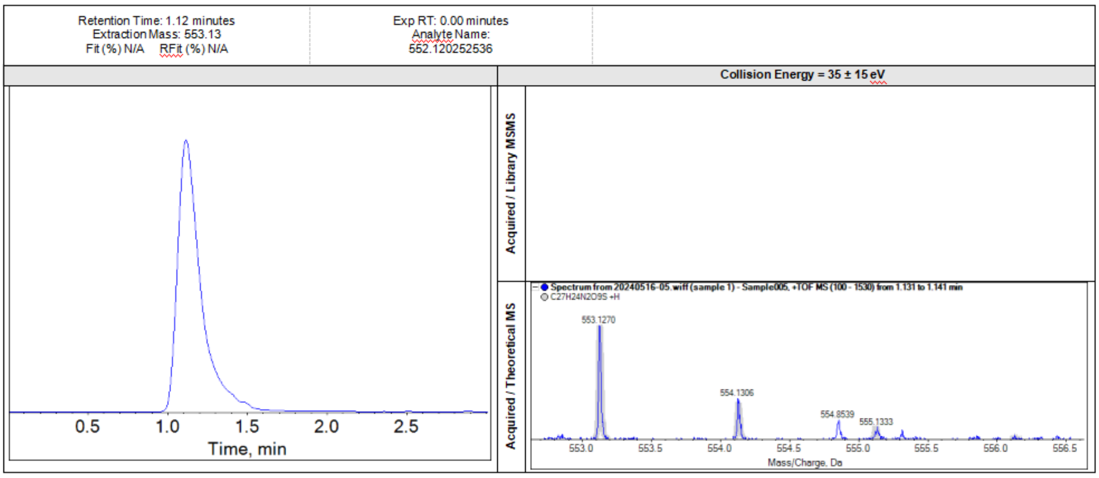

	Compound Name (Library Hit)	Score	Formula	Intensity	Threshold	Expected m/z	Found at m/z	Error (ppm)	Expected RT (min)	Found RT (min)	RT Delta (min)	Isotope Diff (%)	Library Score (%)
✓●✓●●	552.120252536 (No Acquired MSMS)	96%	C27H24N2O9S	54188	5	553.1275	553.1270	-0.9	0.00	1.12	1.12	3.7%	N/A

^{1}H NMR, ^{13}C NMR and HRMS Spectra of Compound 6d

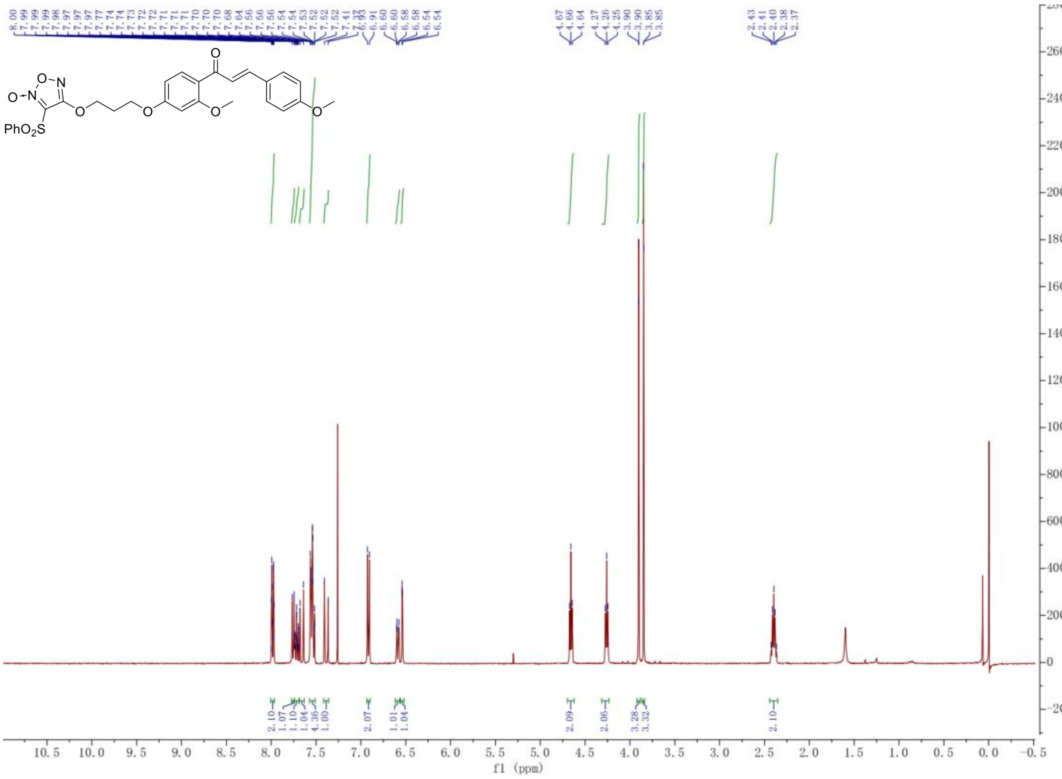

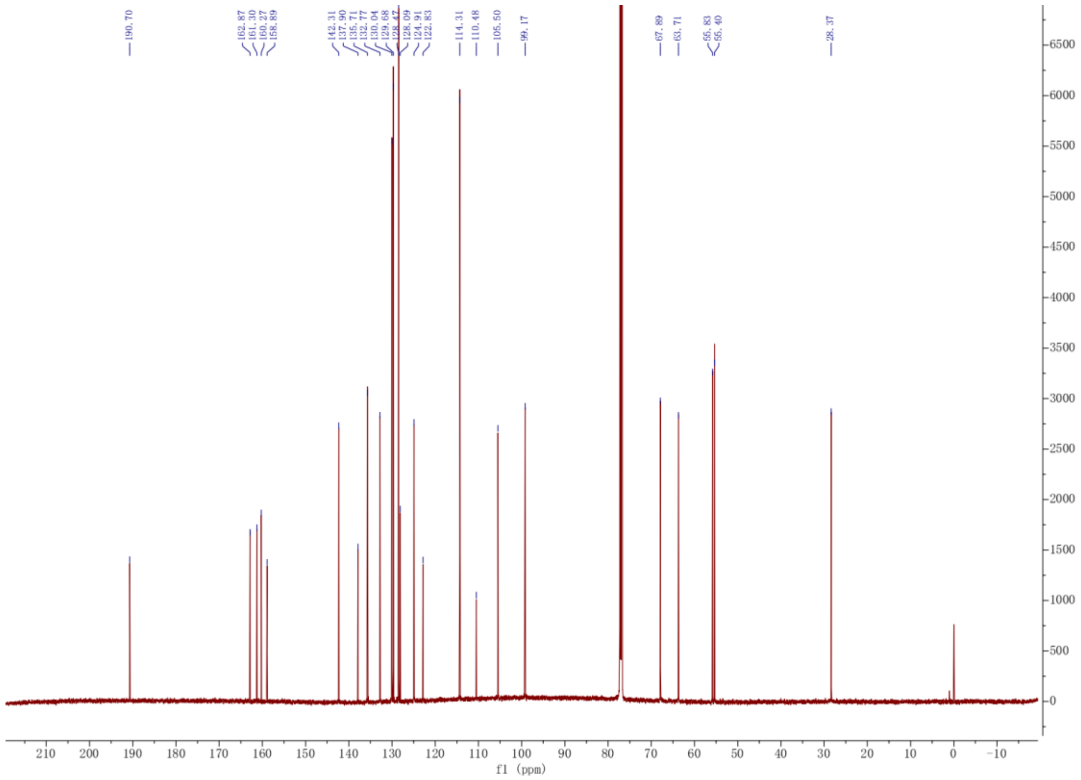

Retention Time: 1.12 minutes<br>Extraction Mass: 567.14<br>Fit (%) N/A RFit (%) N/A	Exp RT: 0.00 minutes<br>Analyte Name:<br>566.135902616	

Collision Energy = 35 ± 15 eV

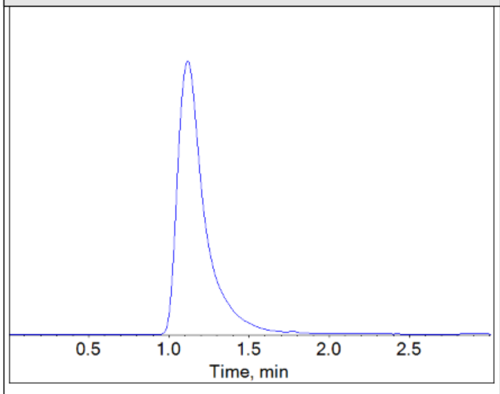

Acquired / Library MSMS

Acquired / Theoretical MS

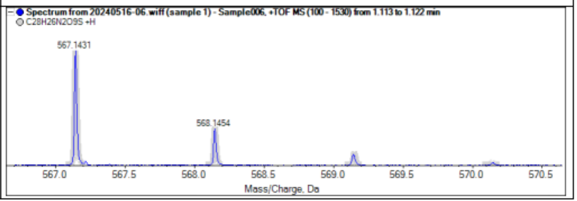

	Compound Name (Library Hit)	Score	Formula	Intensity	Threshold	Expected m/z	Found at m/z	Error (ppm)	Expected RT (min)	Found RT (min)	RT Delta (min)	Isotope Diff (%)	Library Score (%)
✓●✓●●	566.135902616 (No Acquired MSMS)	99%	C28H26N2O9S	1319389	5	567.1432	567.1431	-0.2	0.00	1.12	1.12	2.1%	N/A

^{1}H NMR, ^{13}C NMR and HRMS Spectra of Compound 6e

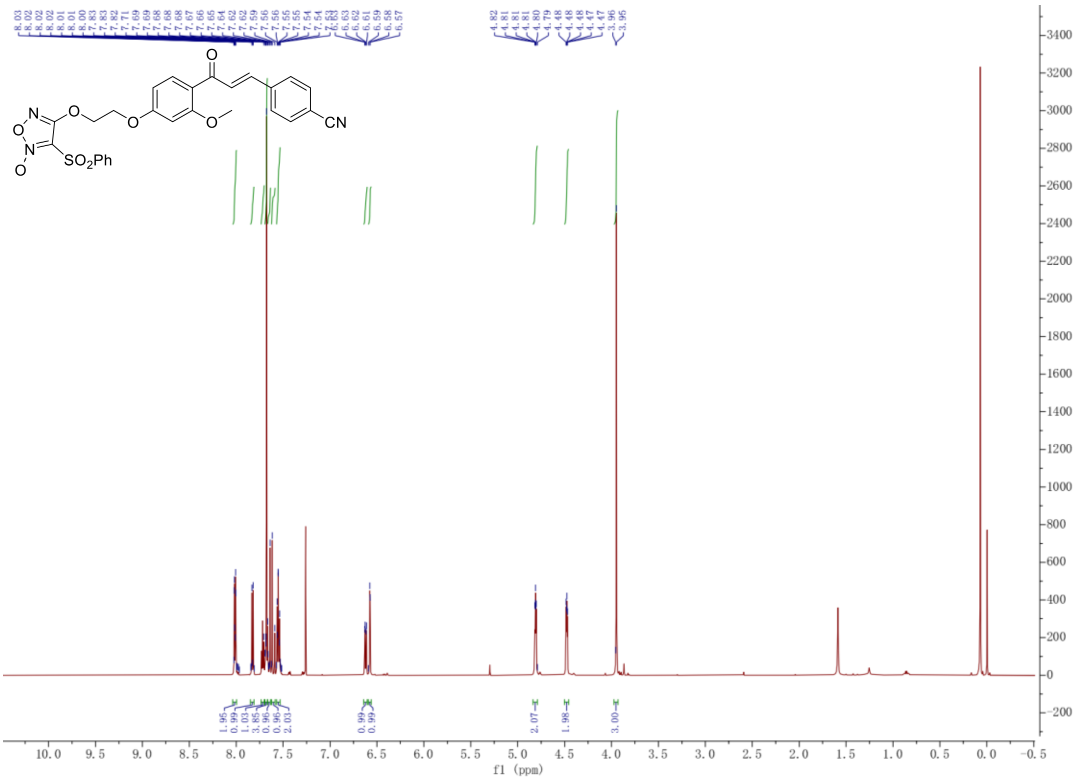

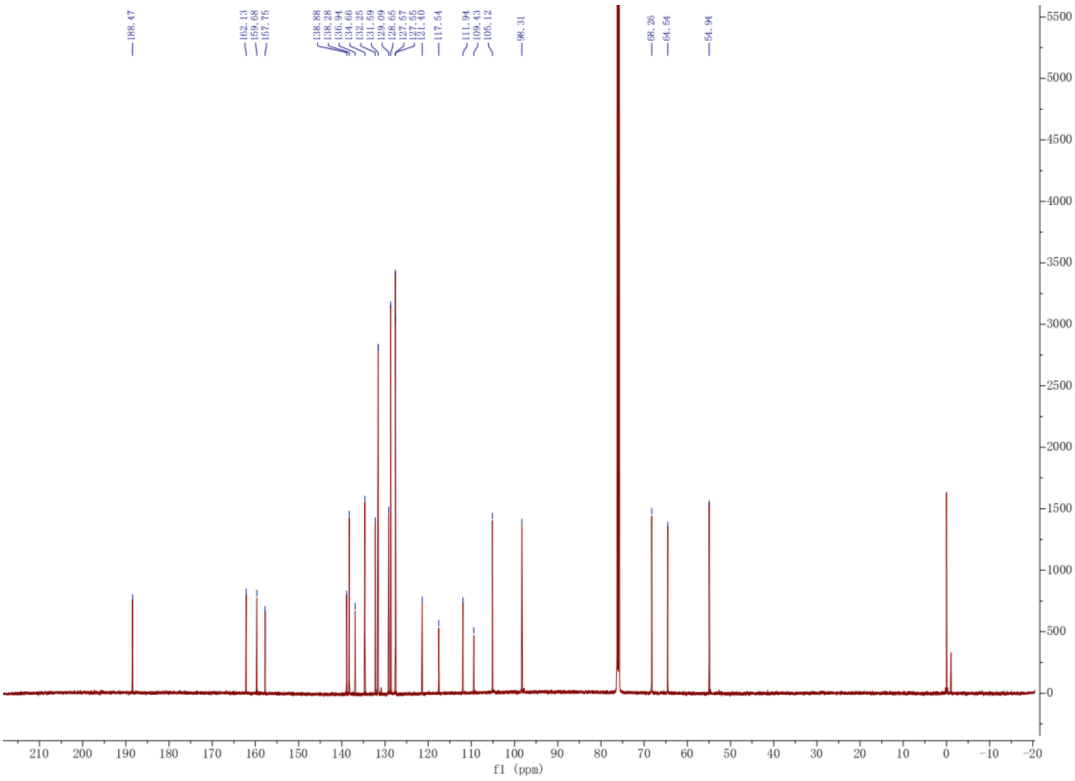

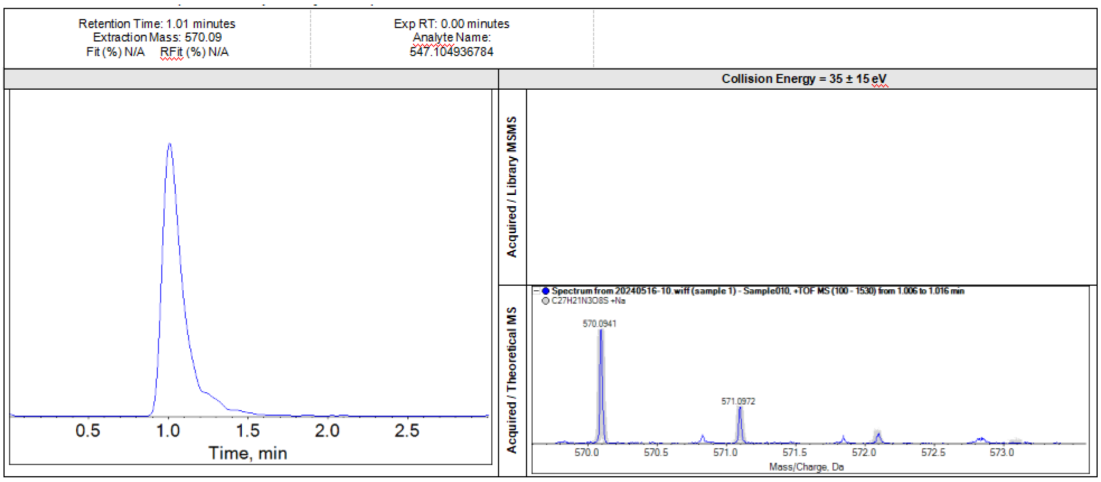

	Compound Name (Library Hit)	Score	Formula	Intensity	Threshold	Expected m/z	Found at m/z	Error (ppm)	Expected RT (min)	Found RT (min)	RT Delta (min)	Isotope Diff (%)	Library Score (%)
	547.104936784 (No Acquired MSMS)	99%	C27H21N3O8S	26843	5	570.0942	570.0941	-0.1	0.00	1.01	1.01	2.4%	N/A

^{1}H NMR, ^{13}C NMR and HRMS Spectra of Compound 6f

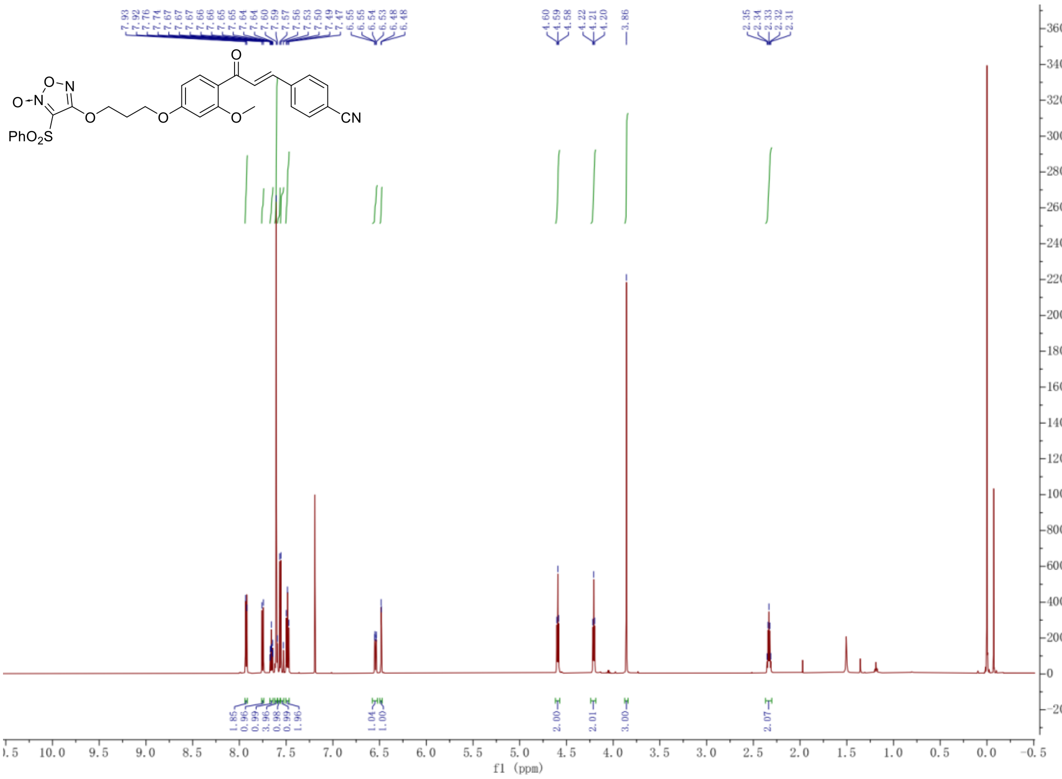

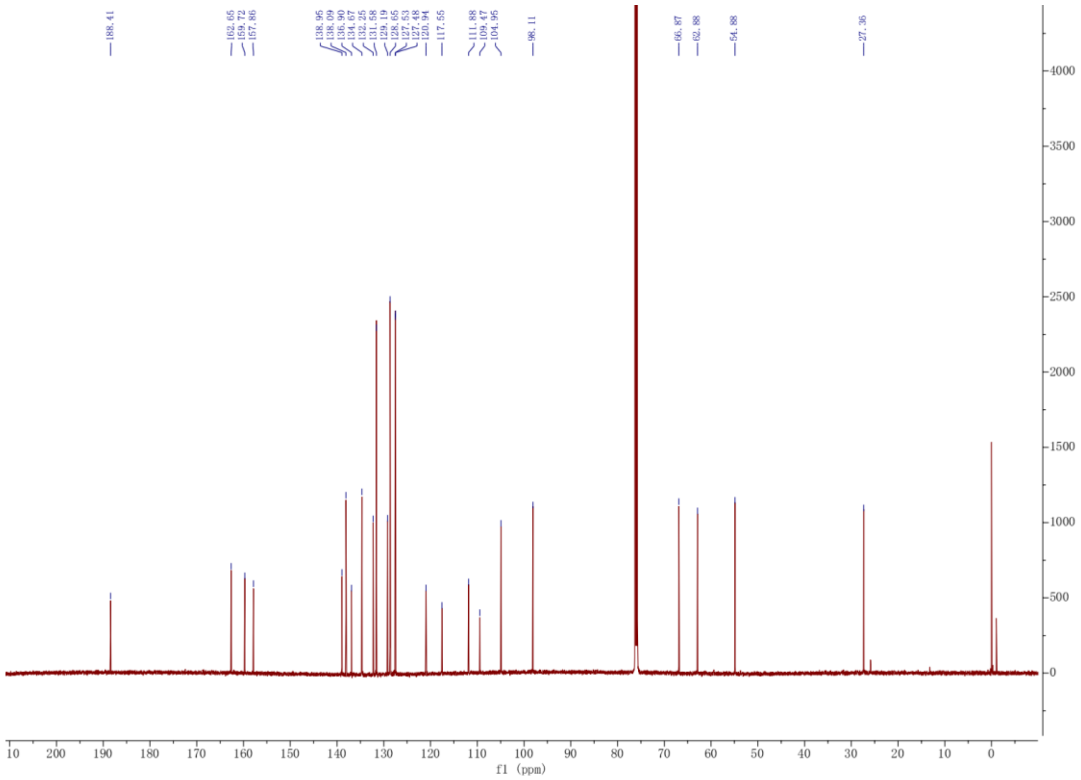

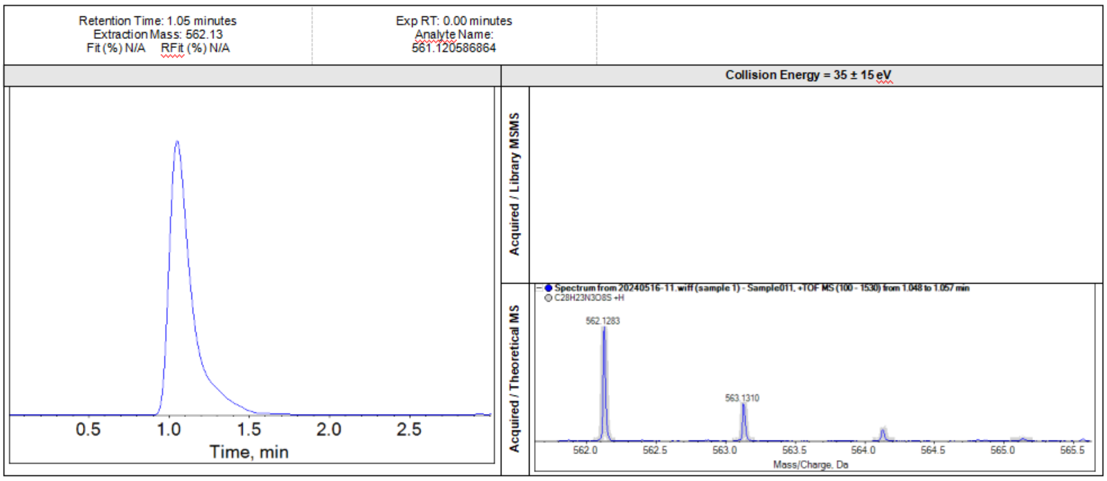

	Compound Name (Library Hit)	Score	Formula	Intensity	Threshold	Expected m/z	Found at m/z	Error (ppm)	Expected RT (min)	Found RT (min)	RT Delta (min)	Isotope Diff (%)	Library Score (%)
✓ ● ✓ ● ●	561.120586864 (No Acquired MSMS)	97%	C28H23N3O8S	104111	5	562.1279	562.1283	0.8	0.00	1.05	1.05	2.0%	N/A

^{1}H NMR, ^{13}C NMR and HRMS Spectra of Compound 9a

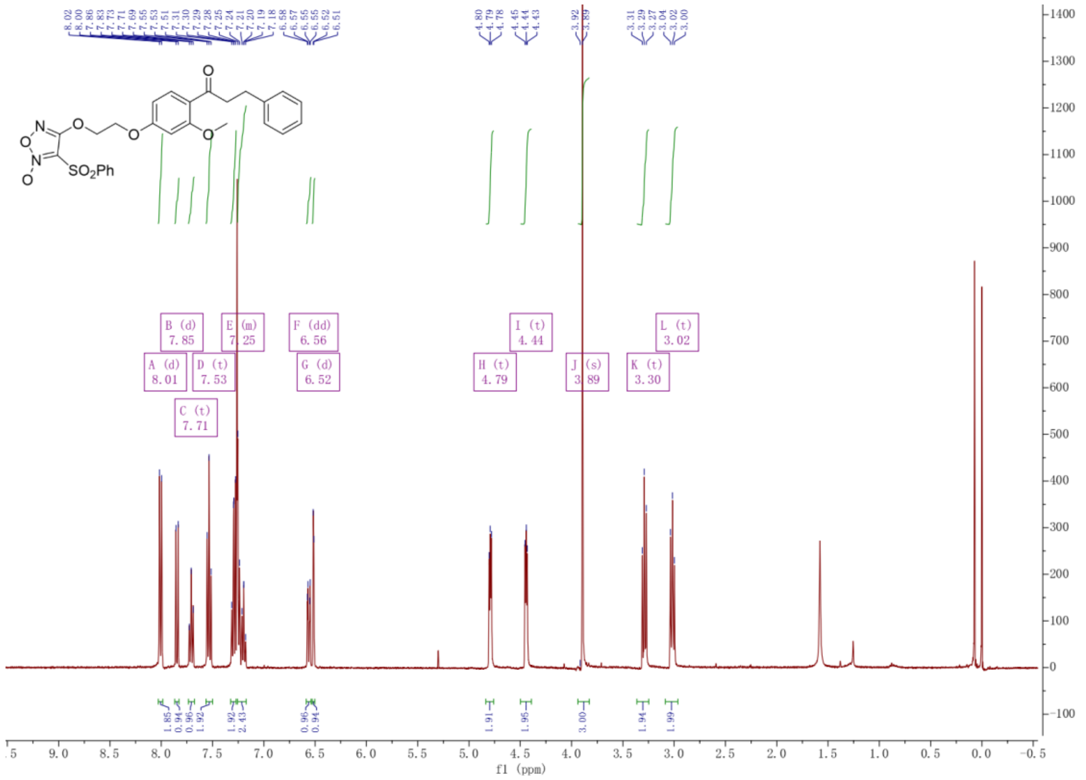

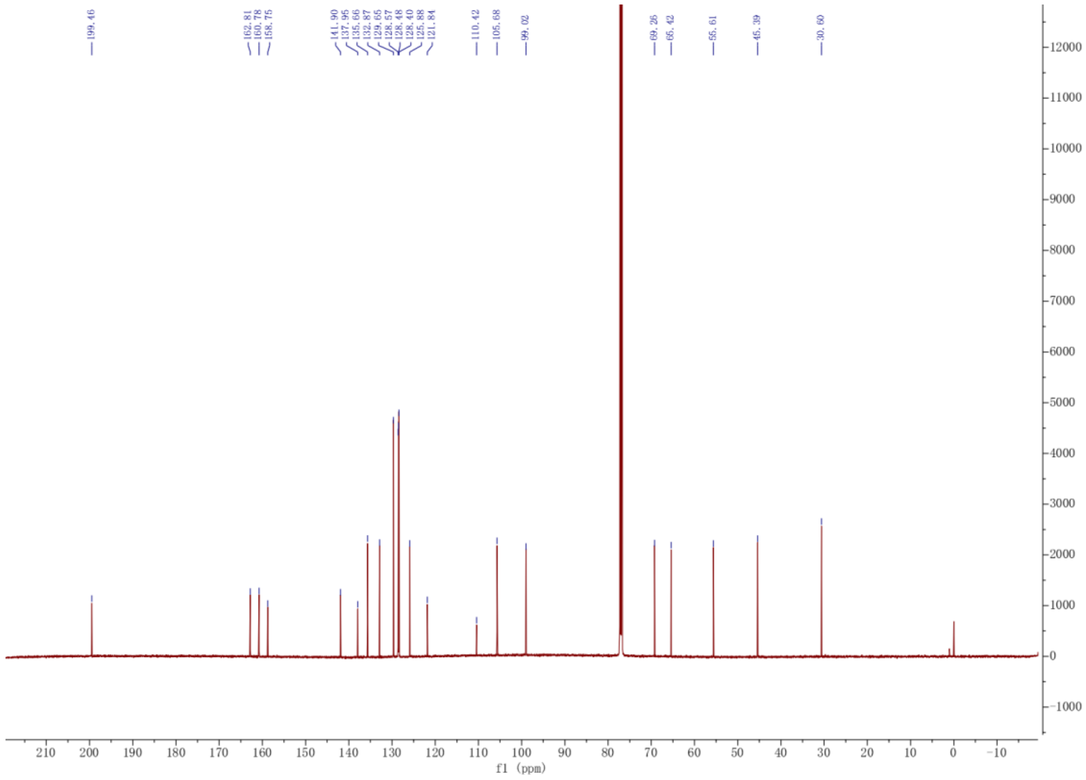

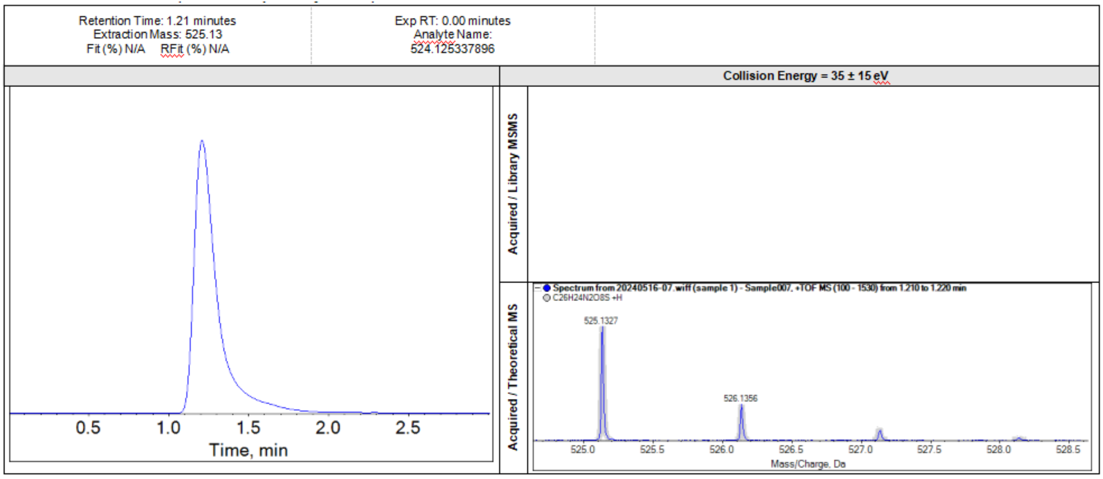

	Compound Name (Library Hit)	Score	Formula	Intensity	Threshold	Expected m/z	Found at m/z	Error (ppm)	Expected RT (min)	Found RT (min)	RT Delta (min)	Isotope Diff (%)	Library Score (%)
	524.125337896 (No Acquired MSMS)	99%	C26H24N2O8S	652398	5	525.1326	525.1327	0.1	0.00	1.21	1.21	2.2%	N/A

^{1}H NMR, ^{13}C NMR and HRMS Spectra of Compound 9b

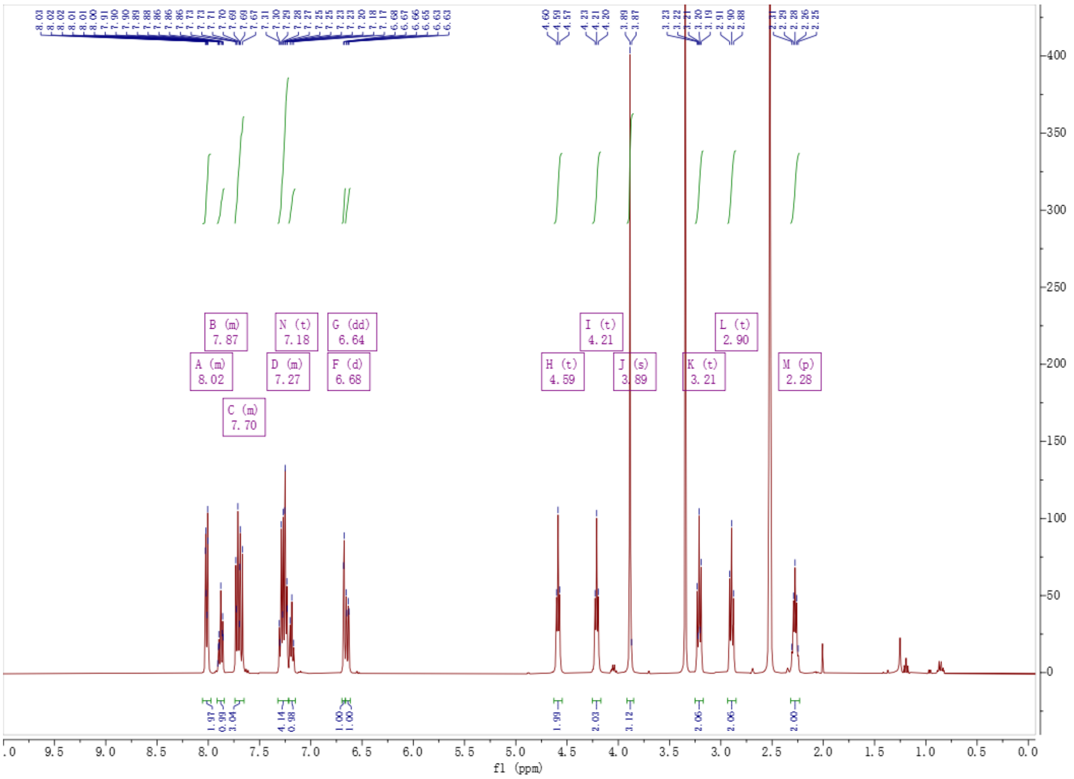

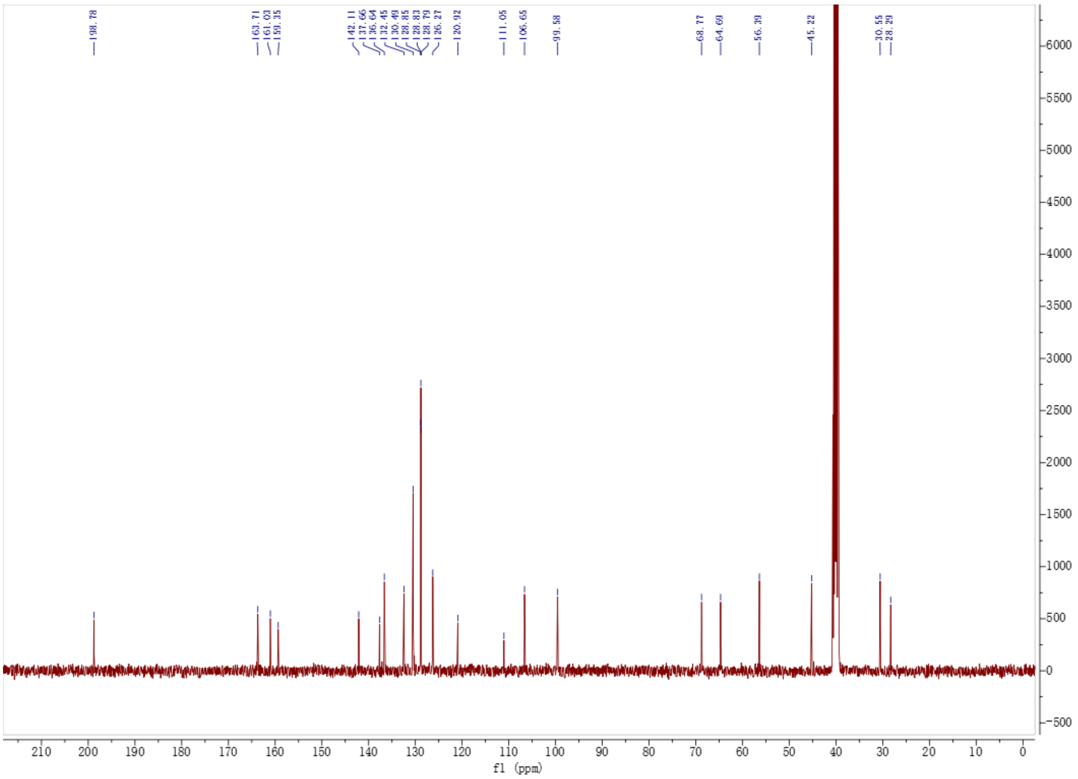

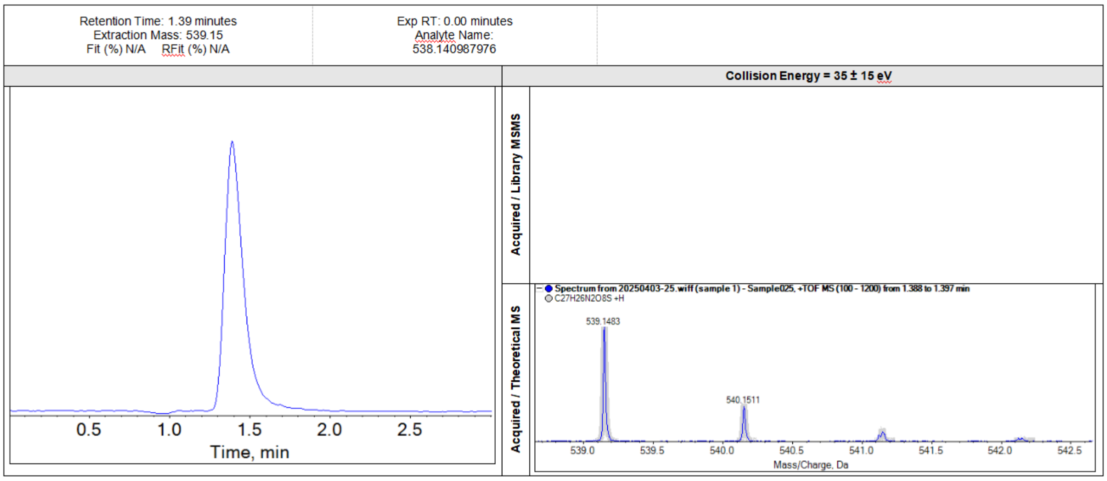

	Compound Name (Library Hit)	Score	Formula	Intensity	Threshold	Expected m/z	Found at m/z	Error (ppm)	Expected RT (min)	Found RT (min)	RT Delta (min)	Isotope Diff (%)	Library Score (%)
	538.140987976 (No data for Library Hit Name xcm)	99%	C27H26N2O8S	347220	5	539.1483	539.1483	0.0	0.00	1.39	1.39	3.1%	N/A

^{1}H NMR, ^{13}C NMR and HRMS Spectra of Compound 9c

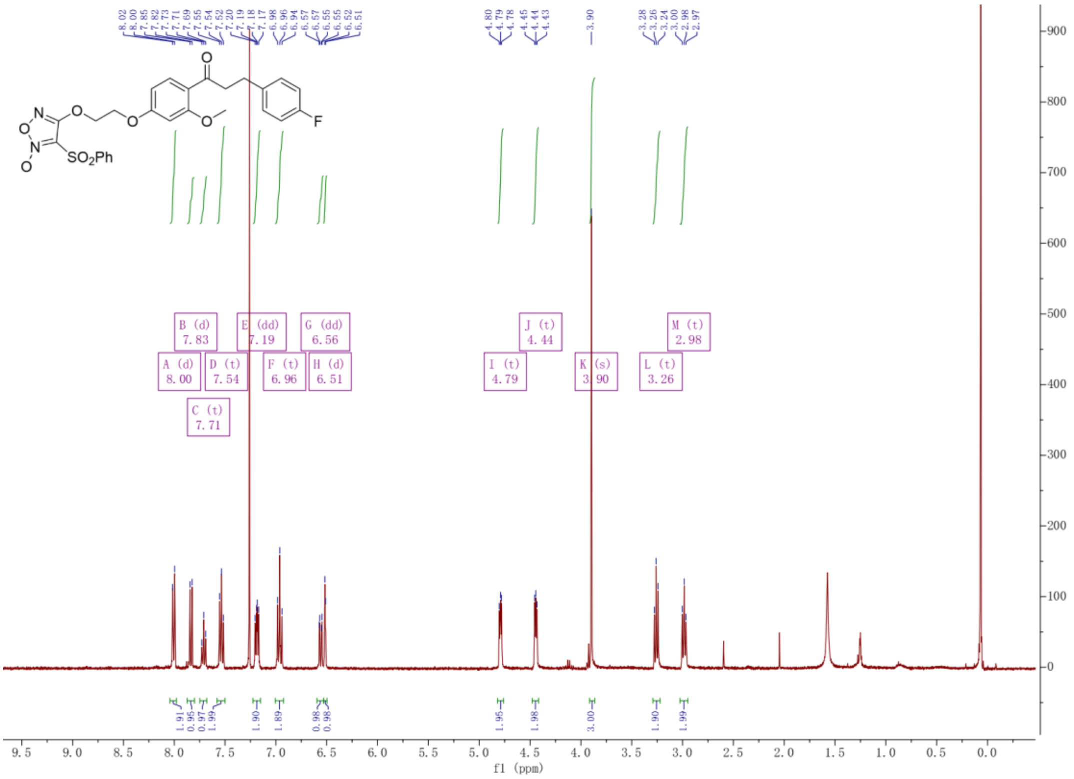

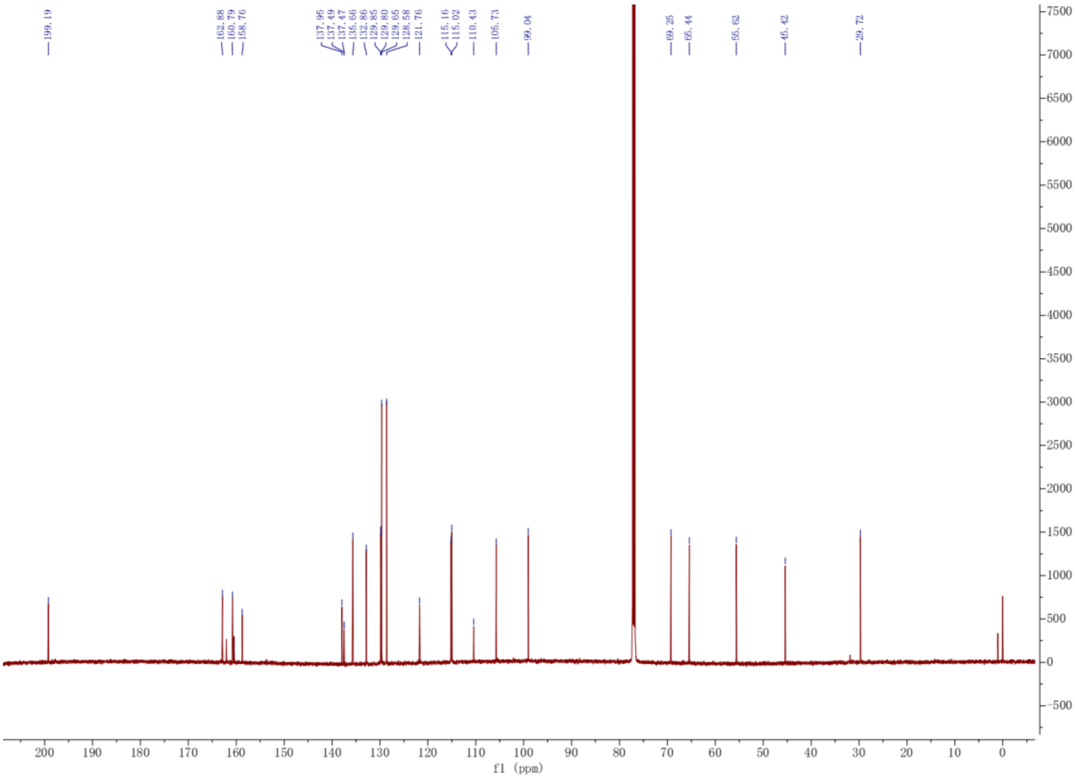

Retention Time: 0.98 minutes
Extraction Mass: 543.12
Fit (%) N/A   RFit (%) N/A

Exp RT: 0.00 minutes
Analyte Name:
542.115916106

Collision Energy = 35 ± 15 eV

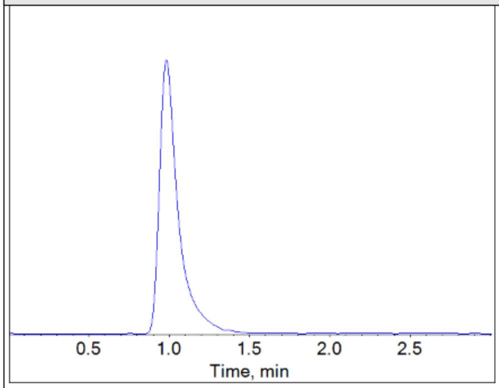

Acquired / Library MSMS

Acquired / Theoretical MS

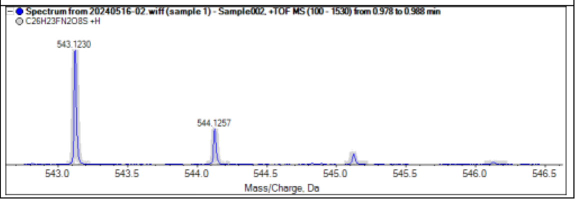

	Compound Name (Library Hit)	Score	Formula	Intensity	Threshold	Expected m/z	Found at m/z	Error (ppm)	Expected RT (min)	Found RT (min)	RT Delta (min)	Isotope Diff (%)	Library Score (%)
✓ ● ✓ ● ●	542.115916106 (No Acquired MSMS)	98%	$C_{26}H_{23}FN_2O_8S$	200138	5	543.1232	543.1230	-0.4	0.00	0.98	0.98	2.0%	N/A

^{1}H NMR, ^{13}C NMR and HRMS Spectra of Compound 9d

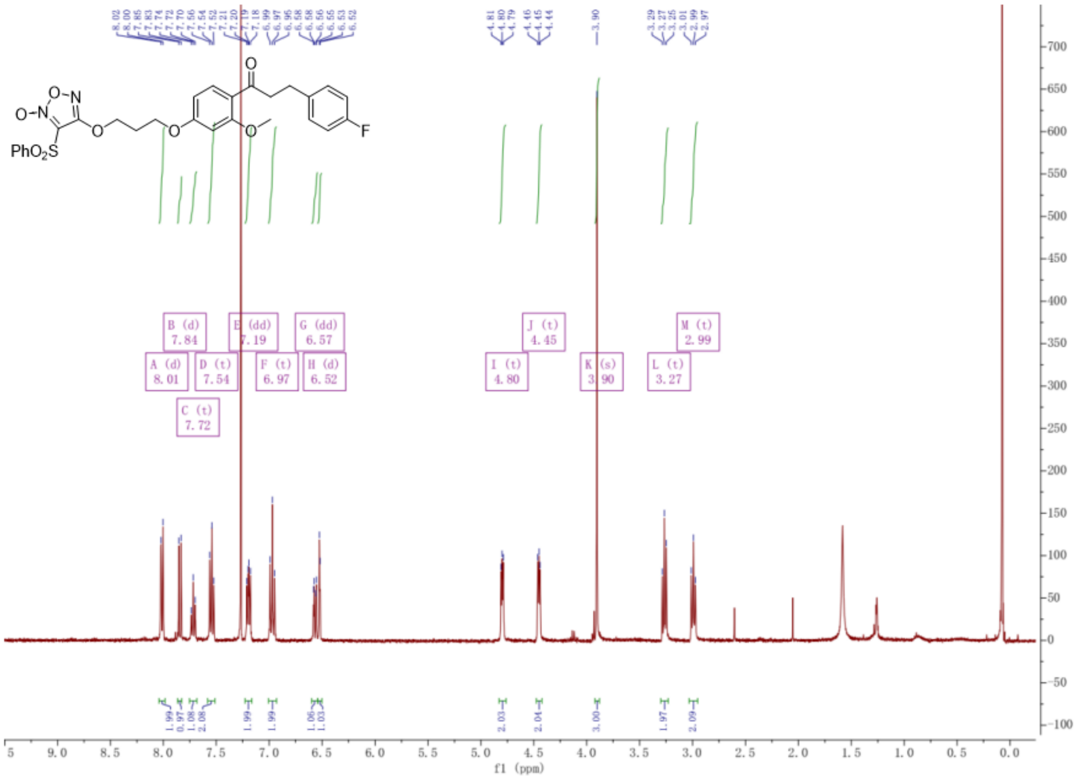

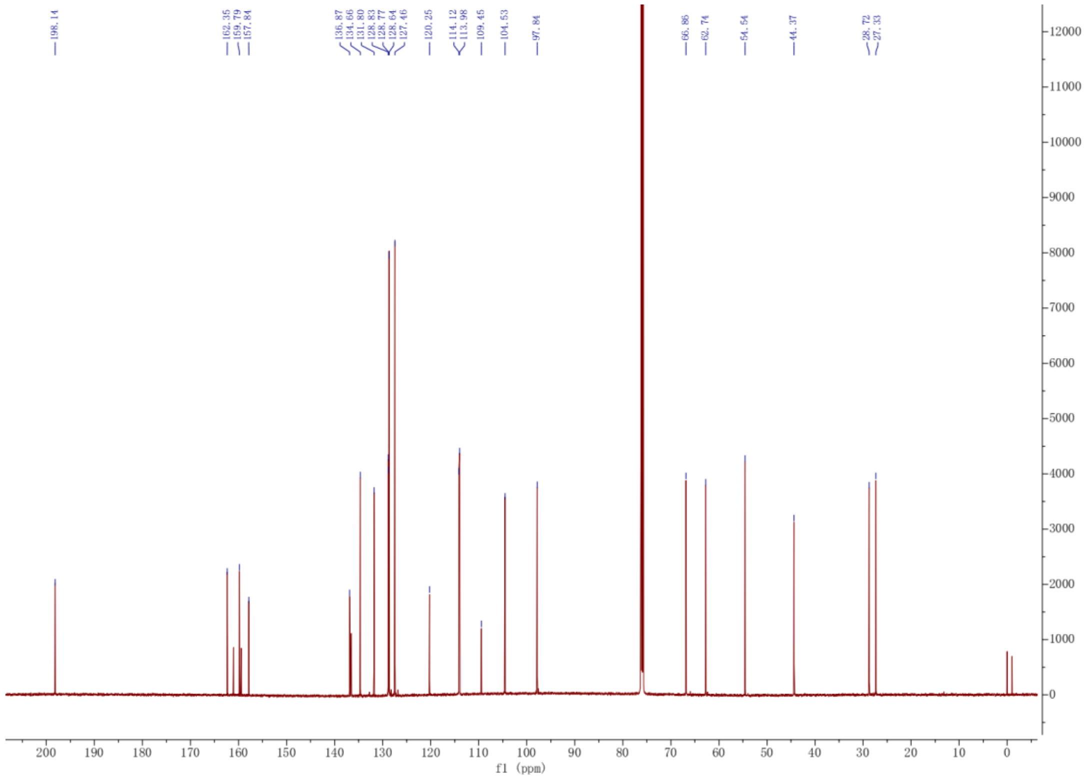

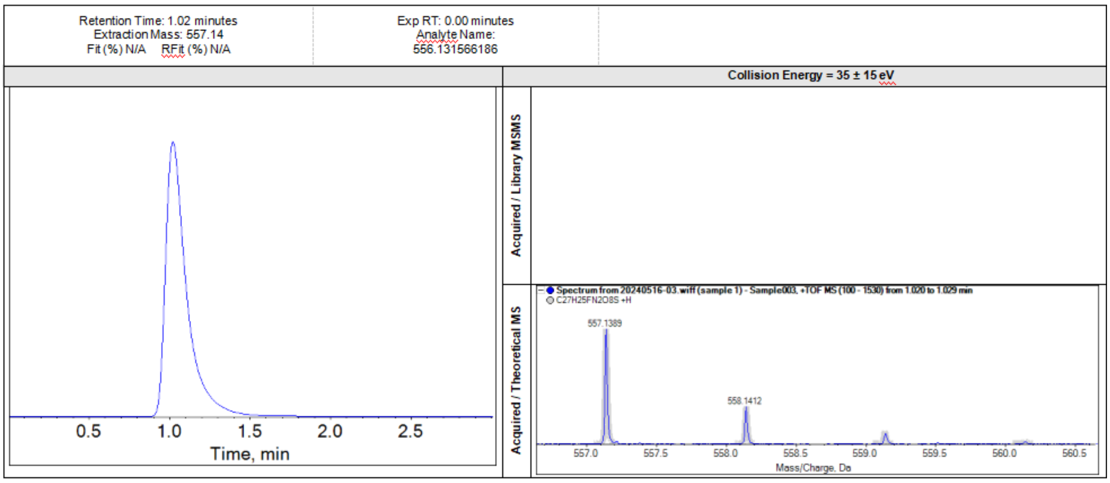

	Compound Name (Library Hit)	Score	Formula	Intensity	Threshold	Expected m/z	Found at m/z	Error (ppm)	Expected RT (min)	Found RT (min)	RT Delta (min)	Isotope Diff (%)	Library Score (%)
	556.131566186 (No Acquired MSMS)	99%	$C_{27}H_{25}FN_2O_8S$	927113	5	557.1388	557.1389	0.1	0.00	1.02	1.02	2.1%	N/A

^{1}H NMR, ^{13}C NMR and HRMS Spectra of Compound 9e

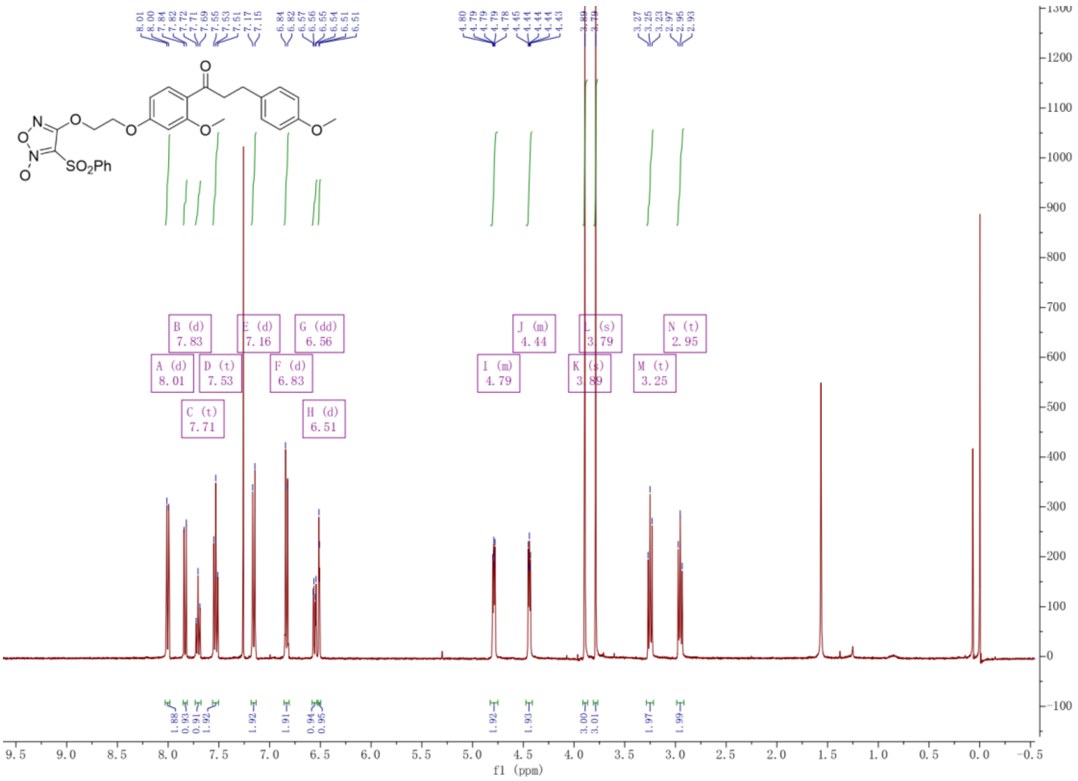

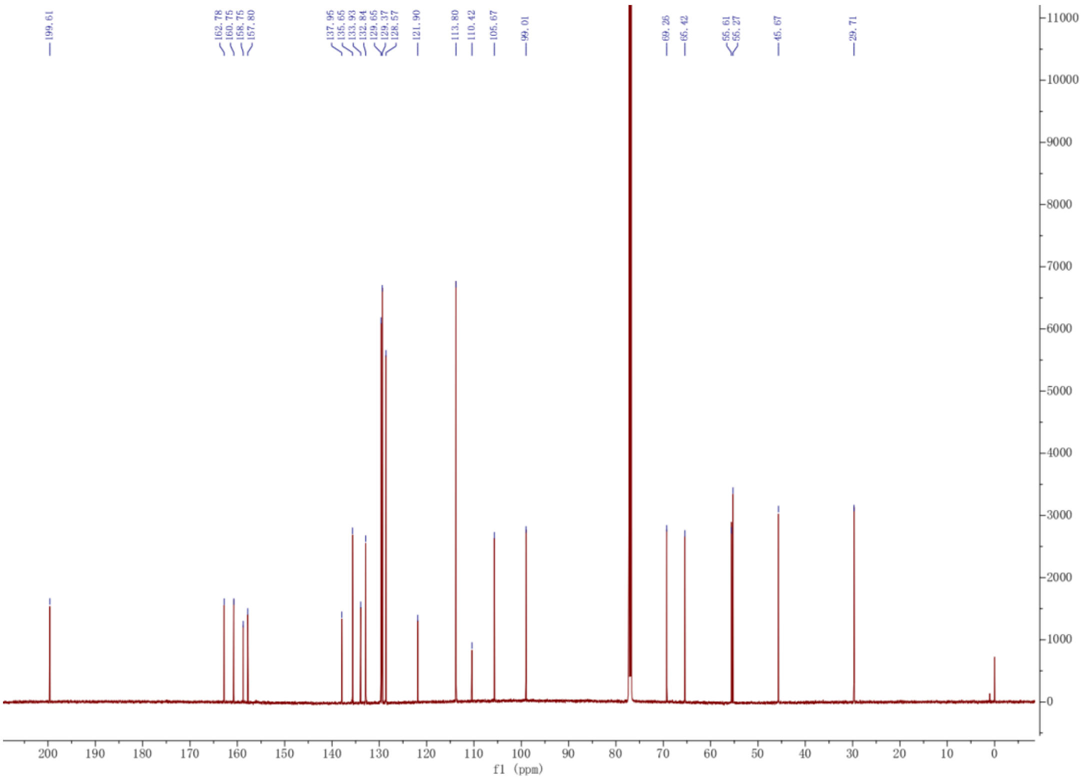

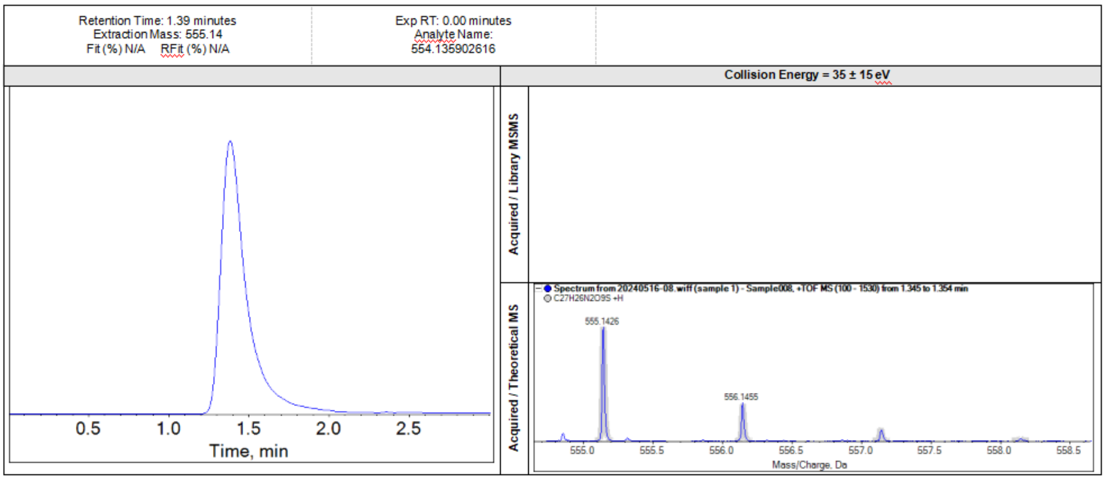

	Compound Name (Library Hit)	Score	Formula	Intensity	Threshold	Expected m/z	Found at m/z	Error (ppm)	Expected RT (min)	Found RT (min)	RT Delta (min)	Isotope Diff (%)	Library Score (%)
✓●✓●●	554.135902616 (No Acquired MSMS)	97%	C27H26N2O9S	110912	5	555.1432	555.1426	-1.0	0.00	1.39	1.39	1.6%	N/A

^{1}H NMR, ^{13}C NMR and HRMS Spectra of Compound 9f

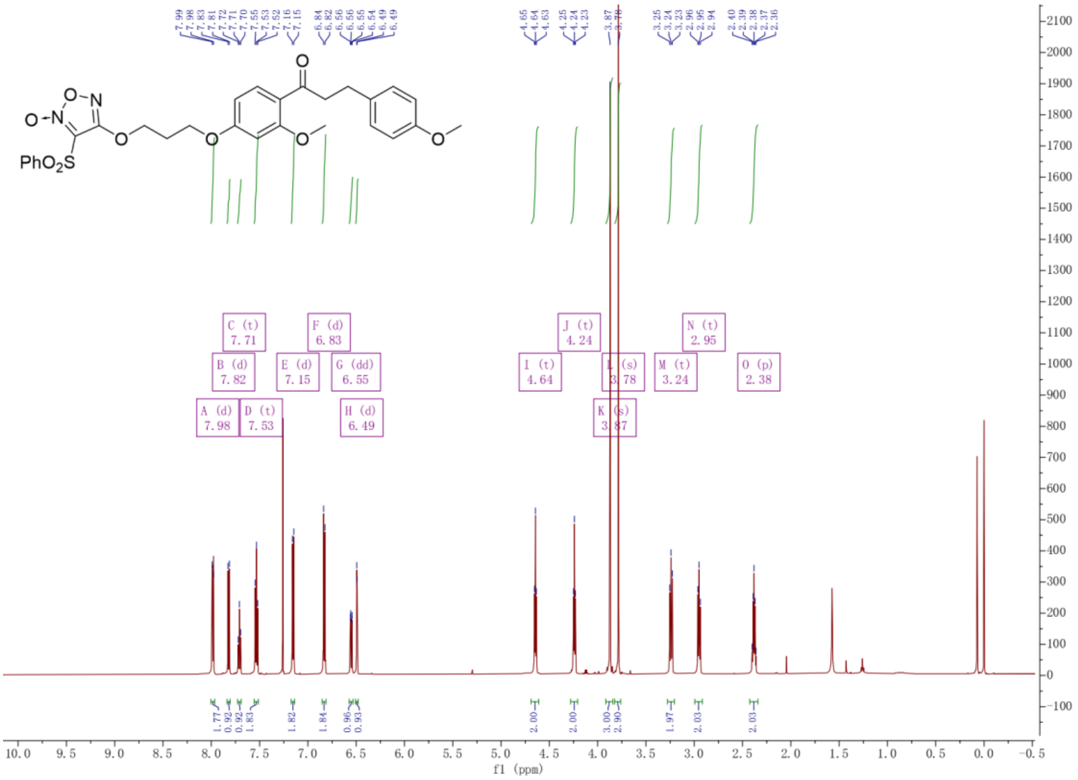

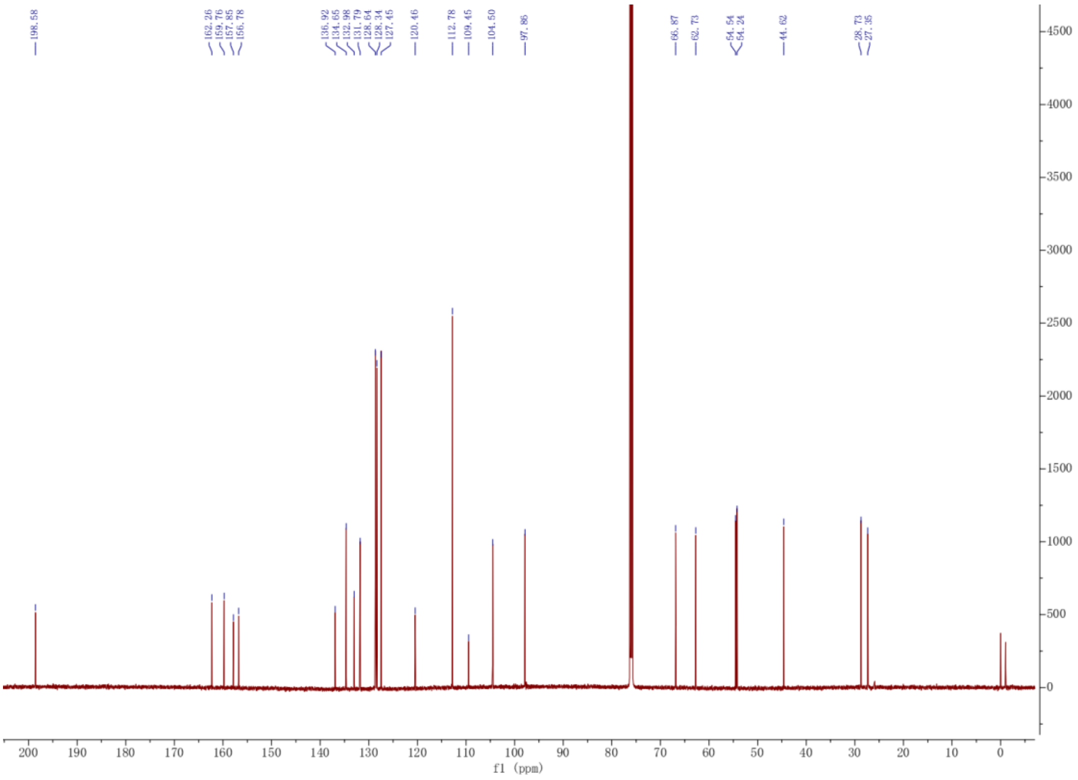

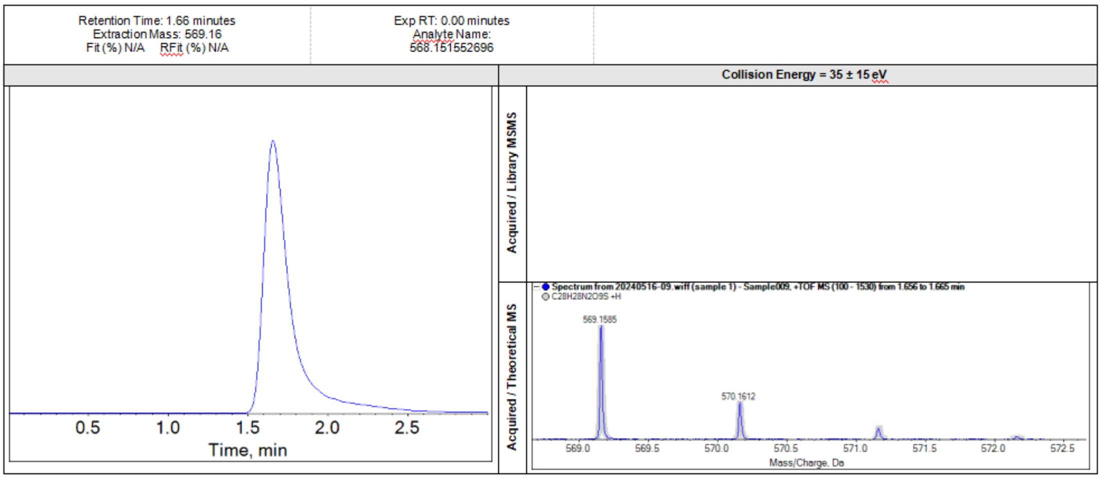

	Compound Name (Library Hit)	Score	Formula	Intensity	Threshold	Expected m/z	Found at m/z	Error (ppm)	Expected RT (min)	Found RT (min)	RT Delta (min)	Isotope Diff (%)	Library Score (%)
	568.151552696 (No Acquired MSMS)	98%	C28H28N2O9S	407056	5	569.1588	569.1585	-0.5	0.00	1.66	1.66	2.4%	N/A

^{1}H NMR, ^{13}C NMR and HRMS Spectra of Compound 12a

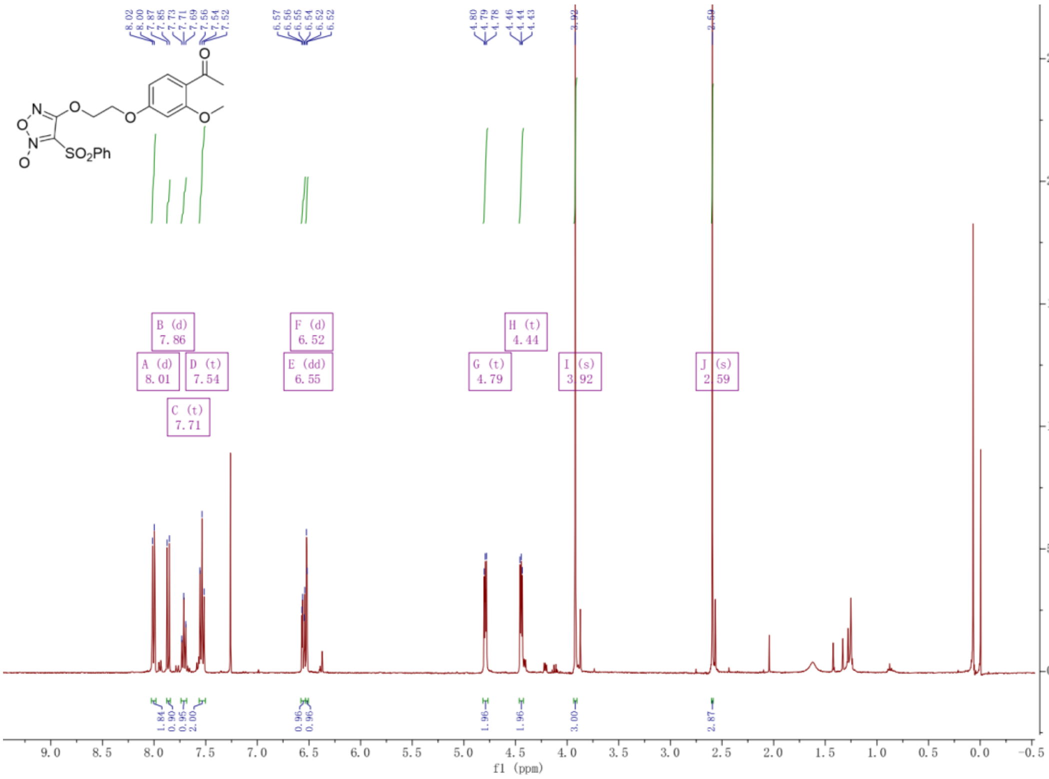

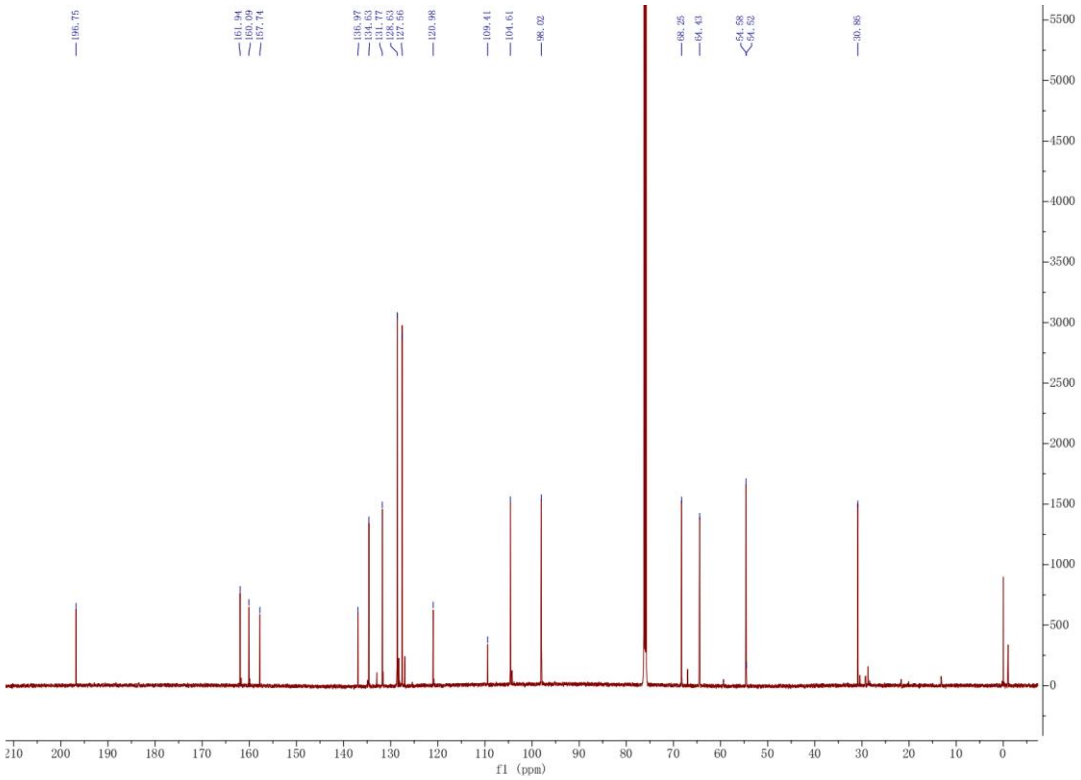

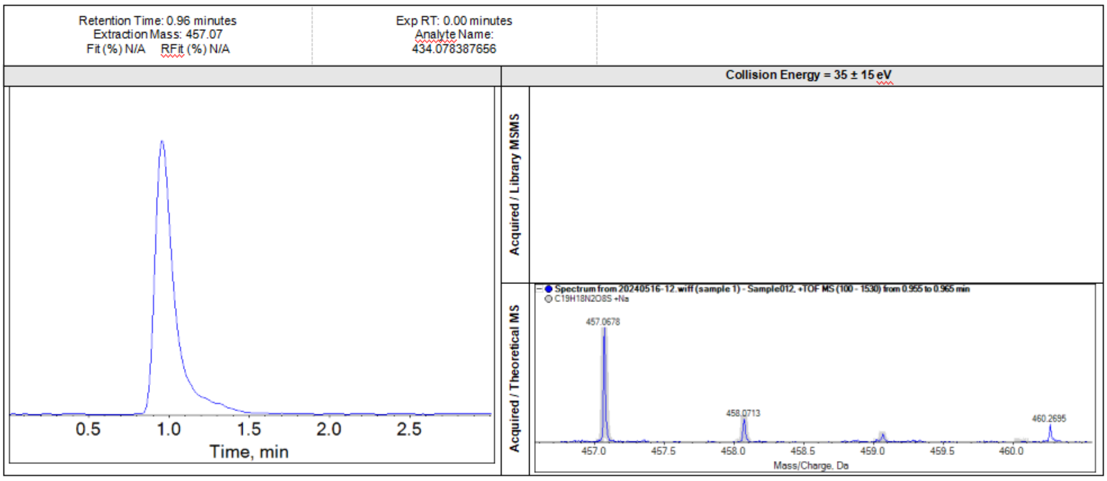

	Compound Name (Library Hit)	Score	Formula	Intensity	Threshold	Expected m/z	Found at m/z	Error (ppm)	Expected RT (min)	Found RT (min)	RT Delta (min)	Isotope Diff (%)	Library Score (%)
✓ ● ✓ ● ●	434.078387656 (No Acquired MSMS)	97%	C19H18N2O8S	55565	5	457.0676	457.0678	0.4	0.00	0.96	0.96	3.5%	N/A

^{1}H NMR, ^{13}C NMR and HRMS Spectra of Compound 12b

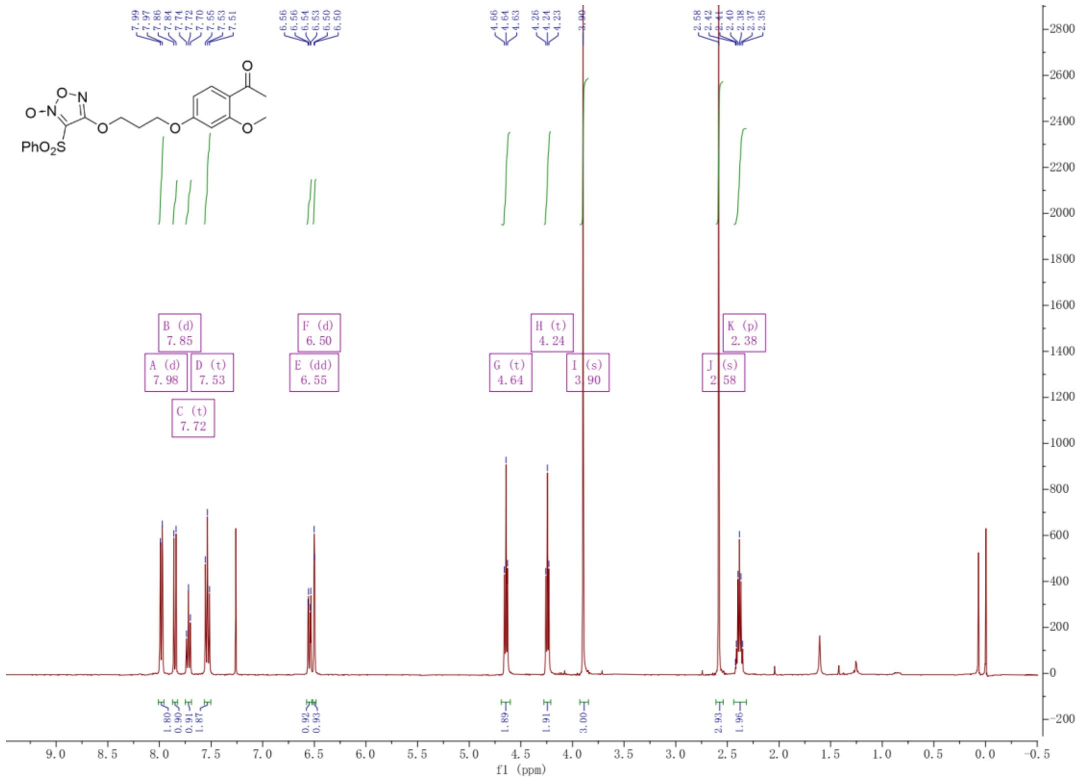

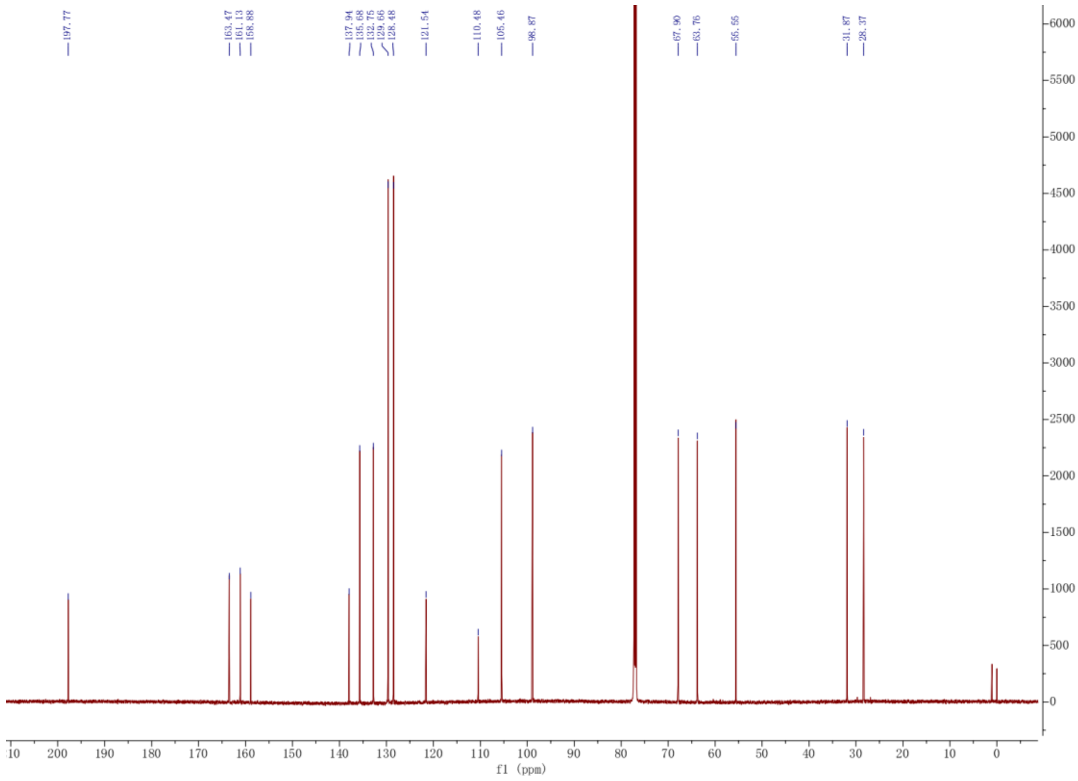

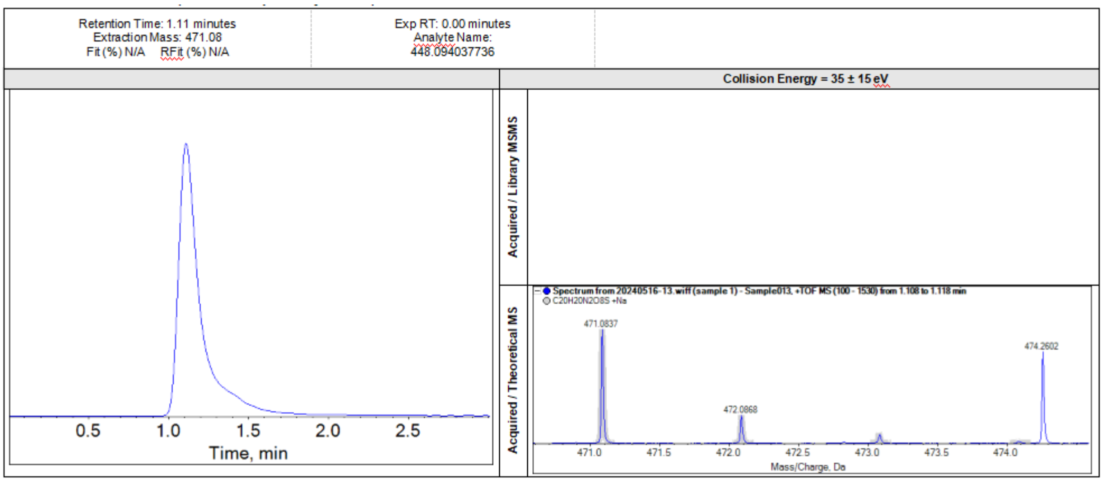

	Compound Name (Library Hit)	Score	Formula	Intensity	Threshold	Expected m/z	Found at m/z	Error (ppm)	Expected RT (min)	Found RT (min)	RT Delta (min)	Isotope Diff (%)	Library Score (%)
	448.094037736 (No Acquired MSMS)	97%	C20H20N2O8S	300326	5	471.0833	471.0837	0.9	0.00	1.11	1.11	1.4%	N/A

^{1}H NMR, ^{13}C NMR and HRMS Spectra of Compound 16a

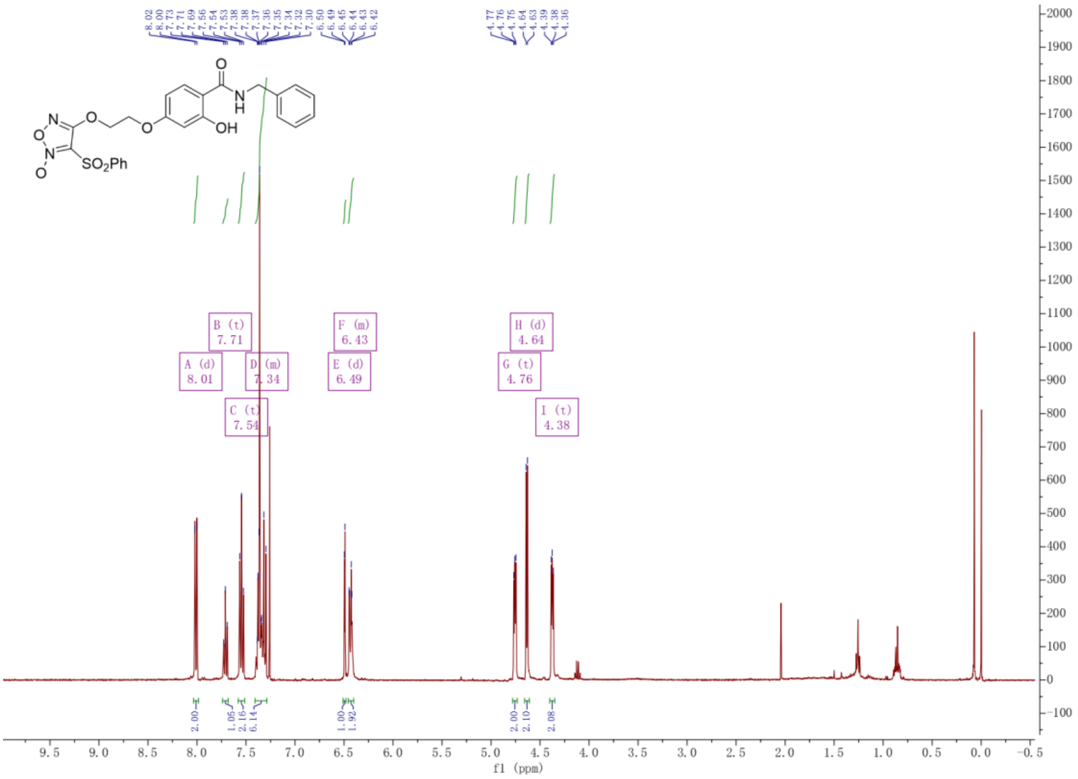

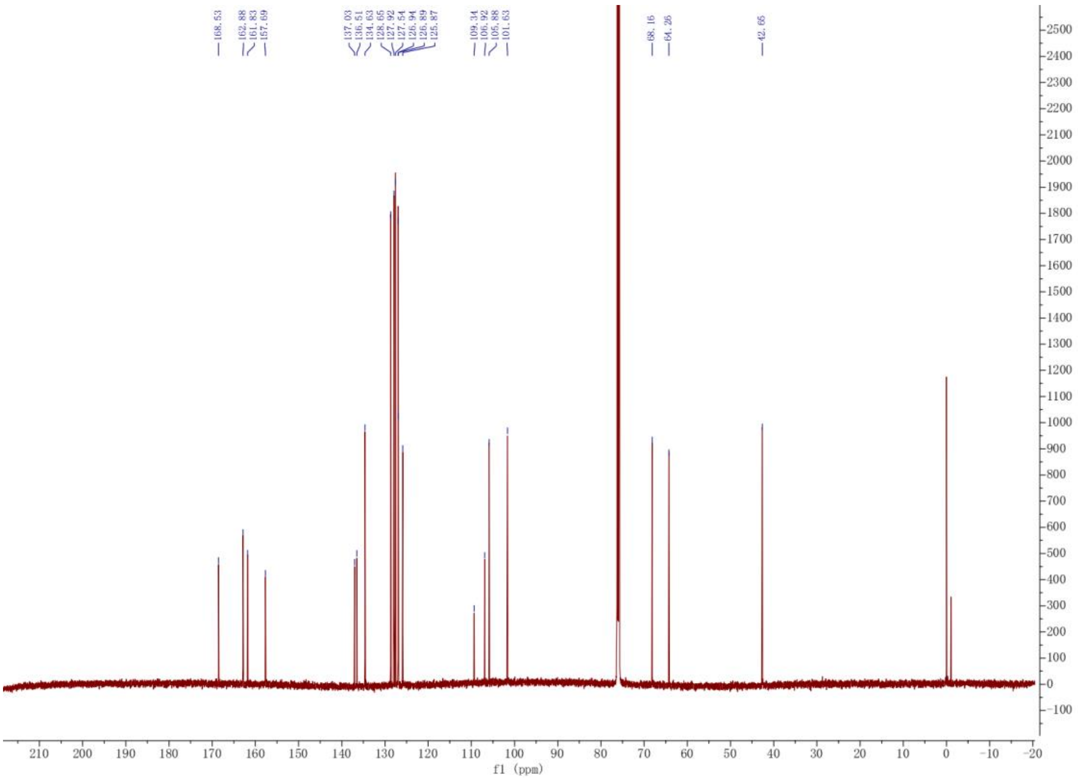

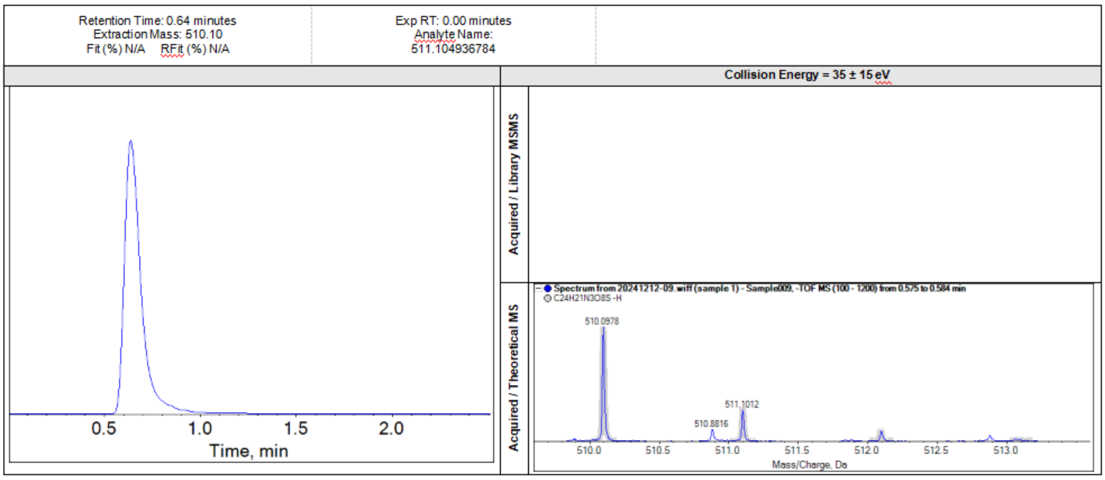

	Compound Name (Library Hit)	Score	Formula	Intensity	Threshold	Expected m/z	Found at m/z	Error (ppm)	Expected RT (min)	Found RT (min)	RT Delta (min)	Isotope Diff (%)	Library Score (%)
✓●✓●●	511.104936784 (No data for Library Hit Name xcm)	97%	C24H21N3O8S	127831	5	510.0977	510.0978	0.4	0.00	0.64	0.64	2.1%	N/A

^{1}H NMR, ^{13}C NMR and HRMS Spectra of Compound 16b

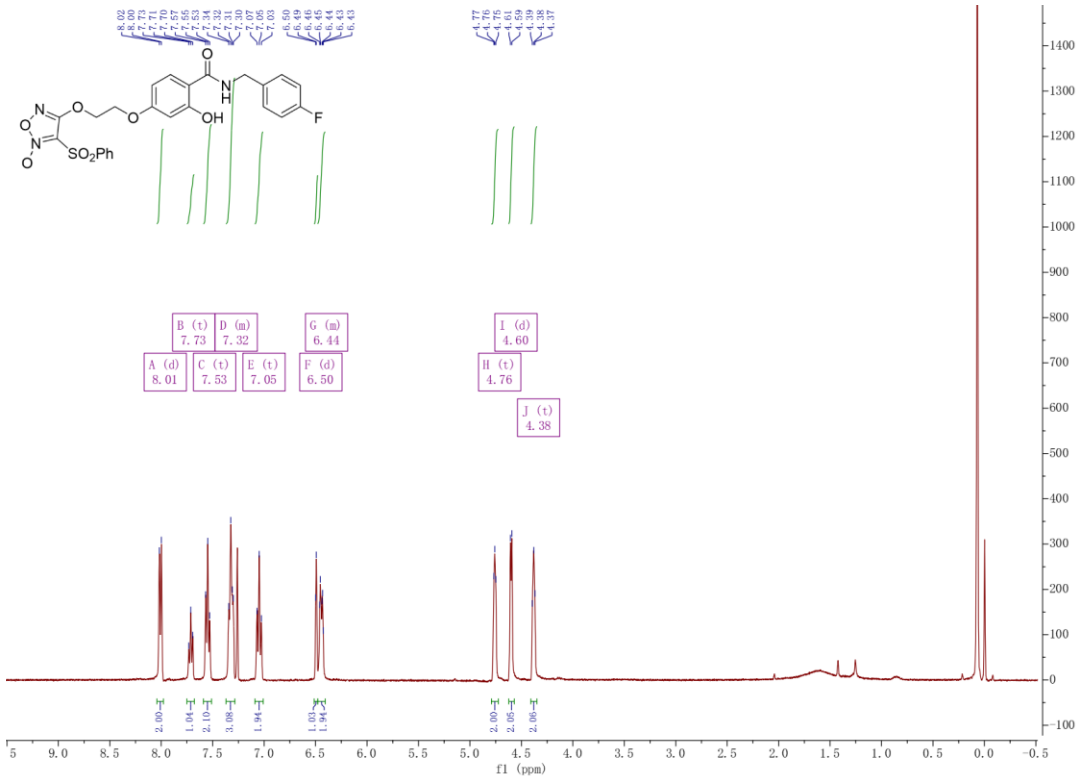

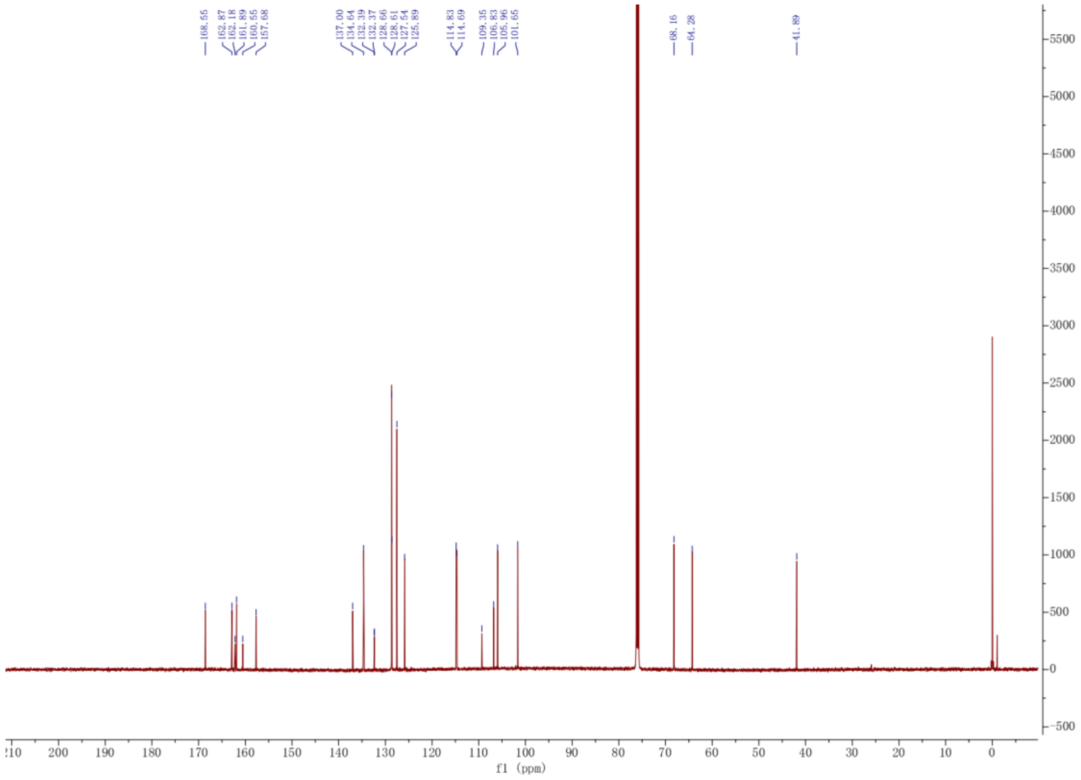

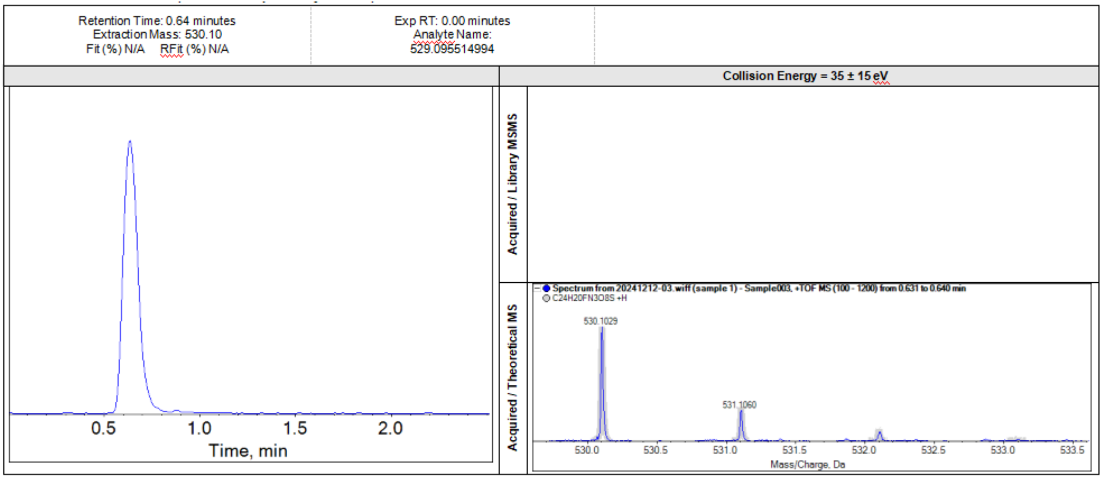

	Compound Name (Library Hit)	Score	Formula	Intensity	Threshold	Expected m/z	Found at m/z	Error (ppm)	Expected RT (min)	Found RT (min)	RT Delta (min)	Isotope Diff (%)	Library Score (%)
	529.095514994 (No data for Library Hit Name xcm)	98%	C24H20FN3 O8S	96458	5	530.1028	530.1029	0.2	0.00	0.64	0.64	2.8%	N/A

^{1}H NMR, ^{13}C NMR and HRMS Spectra of Compound 16c

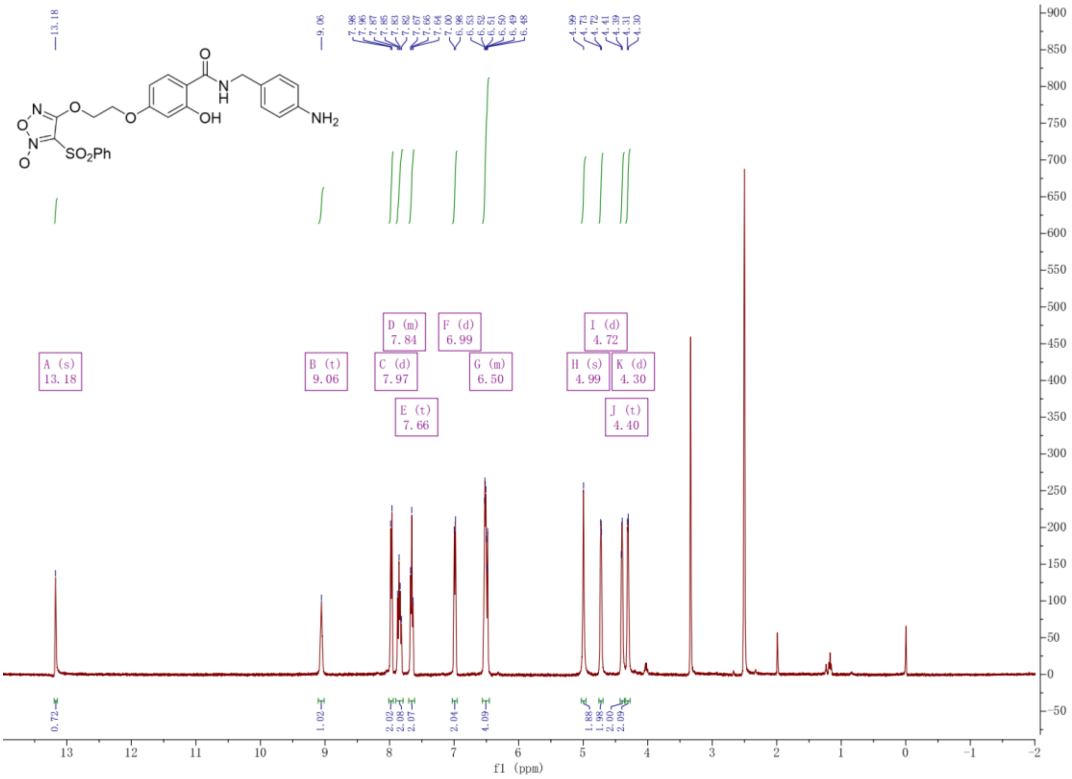

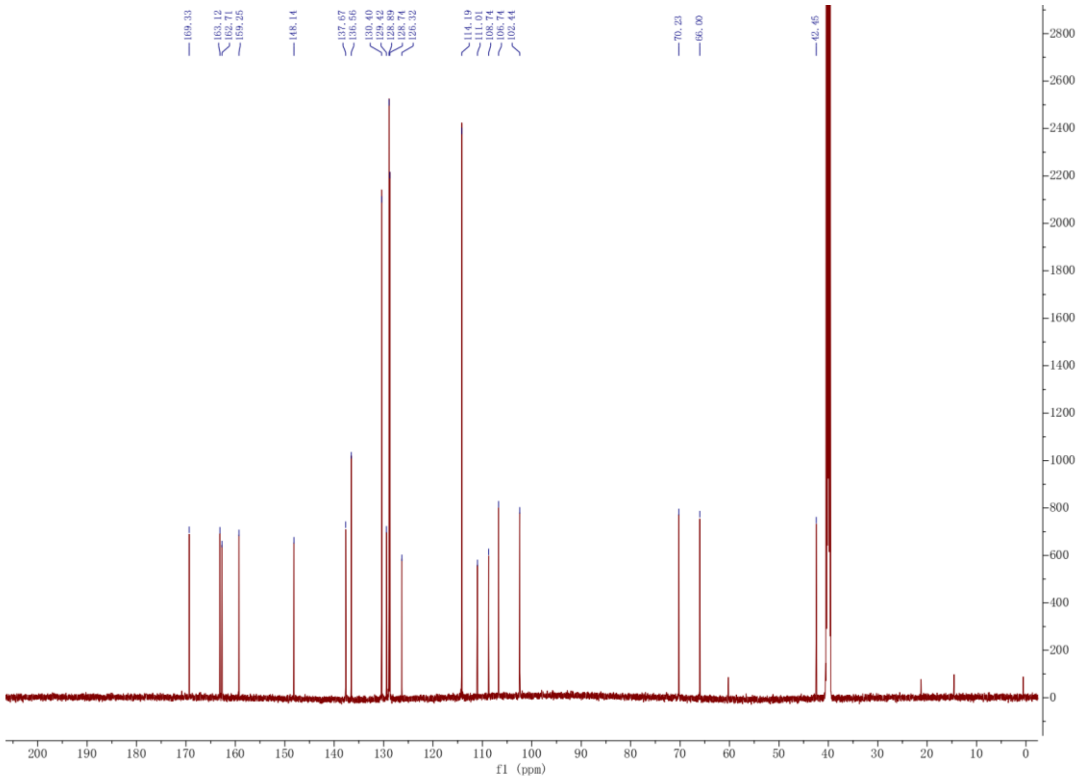

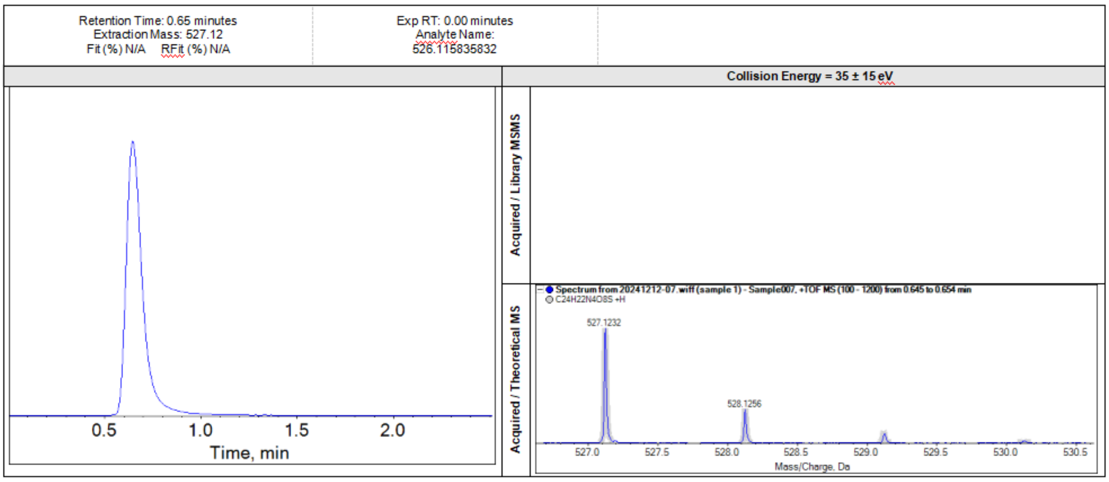

	Compound Name (Library Hit)	Score	Formula	Intensity	Threshold	Expected m/z	Found at m/z	Error (ppm)	Expected RT (min)	Found RT (min)	RT Delta (min)	Isotope Diff (%)	Library Score (%)
	526.115835832 (No data for Library Hit Name xcm)	99%	C24H22N4O8S	934145	5	527.1231	527.1232	0.1	0.00	0.65	0.65	2.3%	N/A

^{1}H NMR, ^{13}C NMR and HRMS Spectra of Compound 16d

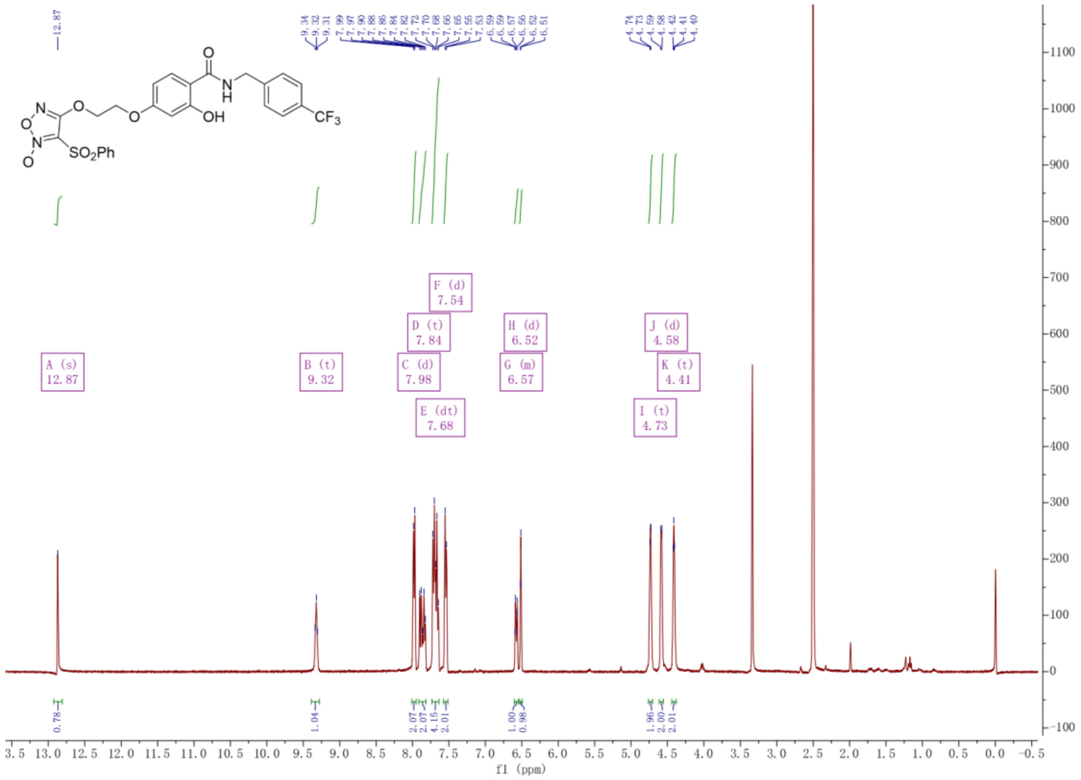

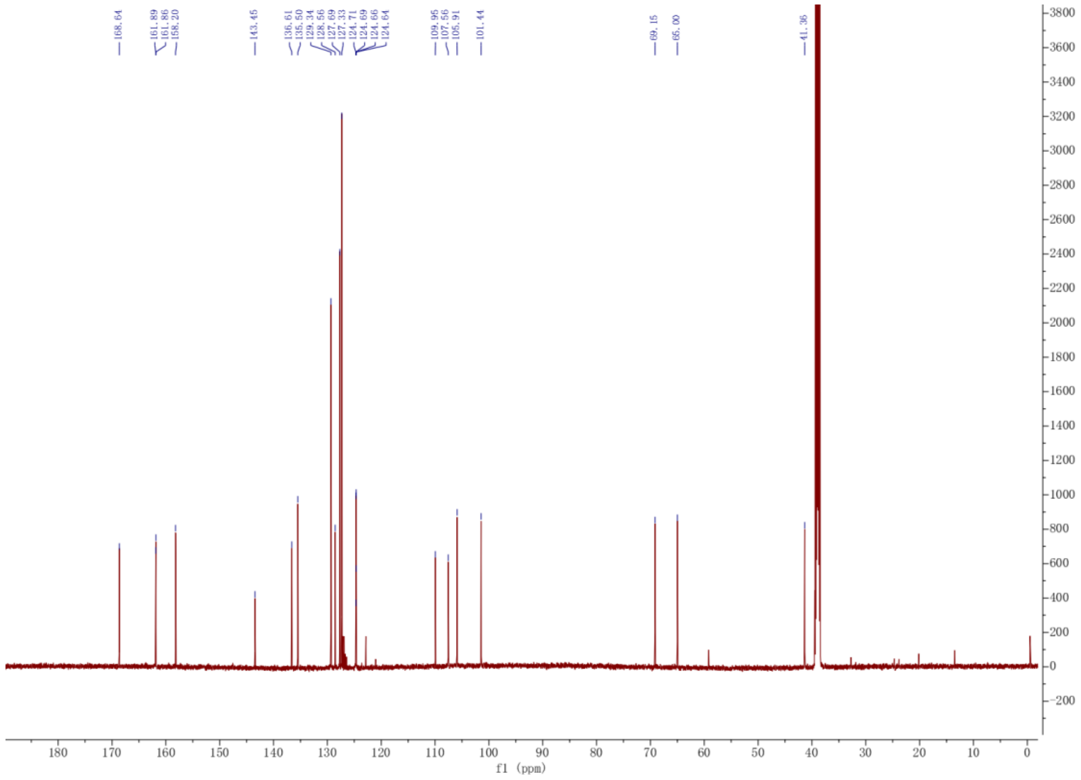

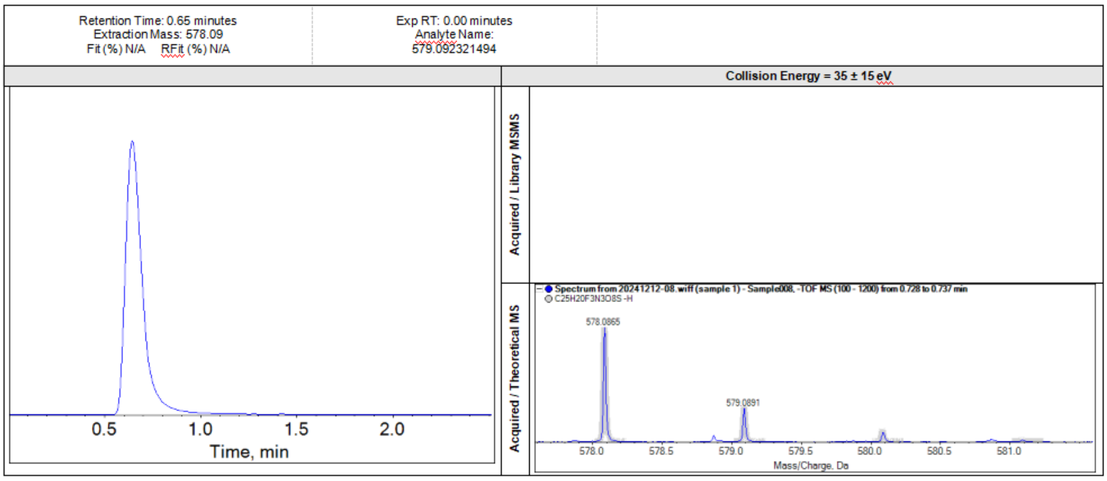

	Compound Name (Library Hit)	Score	Formula	Intensity	Threshold	Expected m/z	Found at m/z	Error (ppm)	Expected RT (min)	Found RT (min)	RT Delta (min)	Isotope Diff (%)	Library Score (%)
	579.092321494 (No data for Library Hit Name xcm)	79%	C25H20F3N3 O8S	180629	5	578.0850	578.0865	2.6	0.00	0.65	0.65	2.4%	N/A

^{1}H NMR, ^{13}C NMR and HRMS Spectra of Compound 16e

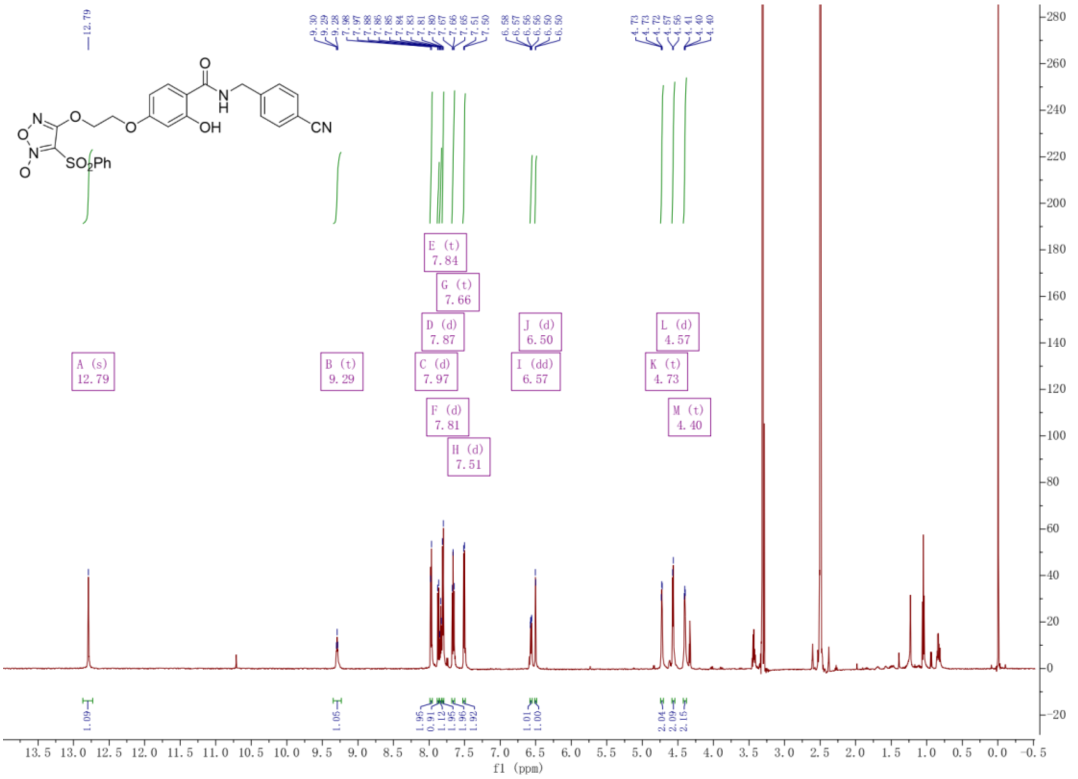

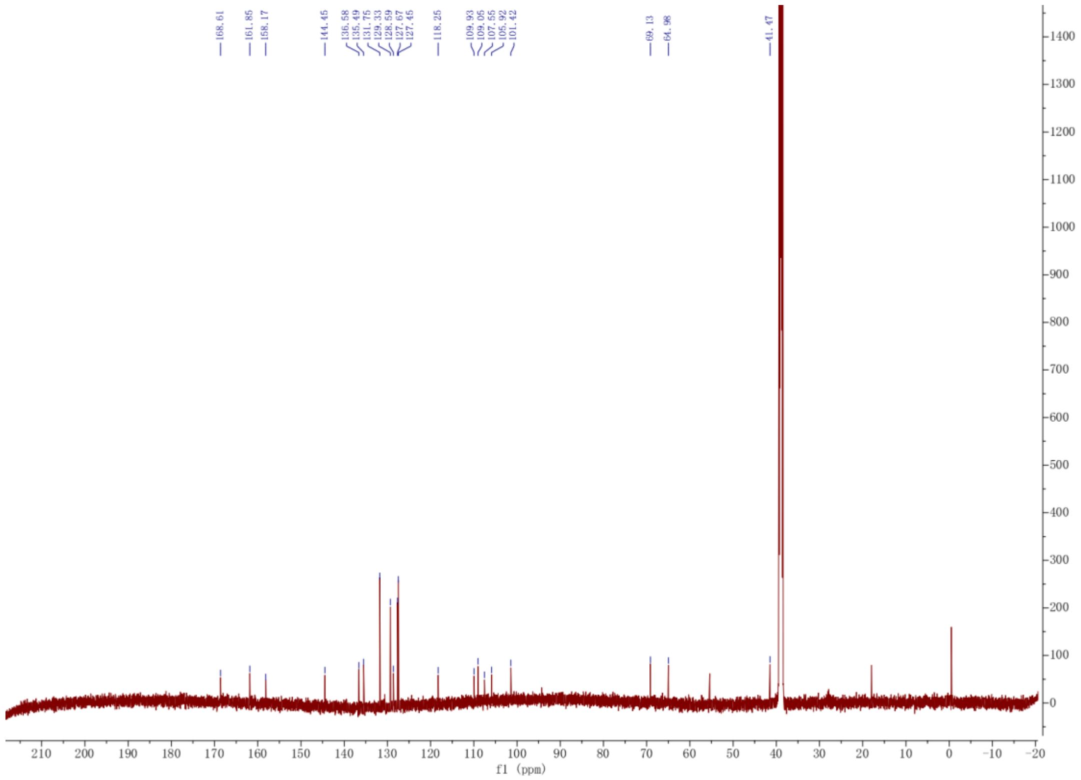

Retention Time: 0.60 minutes<br>Extraction Mass: 551.12<br>Fit (%) N/A RFit (%) N/A	Exp RT: 0.00 minutes<br>Analyte Name:<br>550.115835832

Collision Energy = 35 ± 15 eV

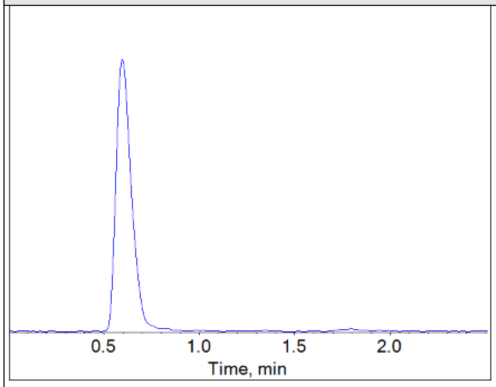

Acquired / Library MSMS

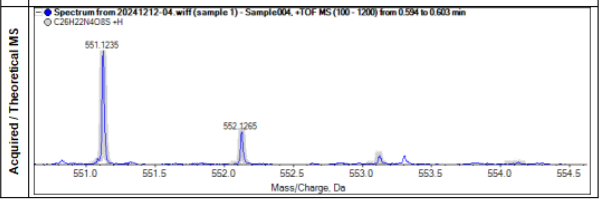

	Compound Name (Library Hit)	Score	Formula	Intensity	Threshold	Expected m/z	Found at m/z	Error (ppm)	Expected RT (min)	Found RT (min)	RT Delta (min)	Isotope Diff (%)	Library Score (%)
✓ ● ✓ ● ●	550.115835832 (No data for Library Hit Name.xcm)	93%	C26H22N4O8S	68653	5	551.1231	551.1235	0.8	0.00	0.60	0.60	3.4%	N/A

^{1}H NMR, ^{13}C NMR and HRMS Spectra of Compound 16f

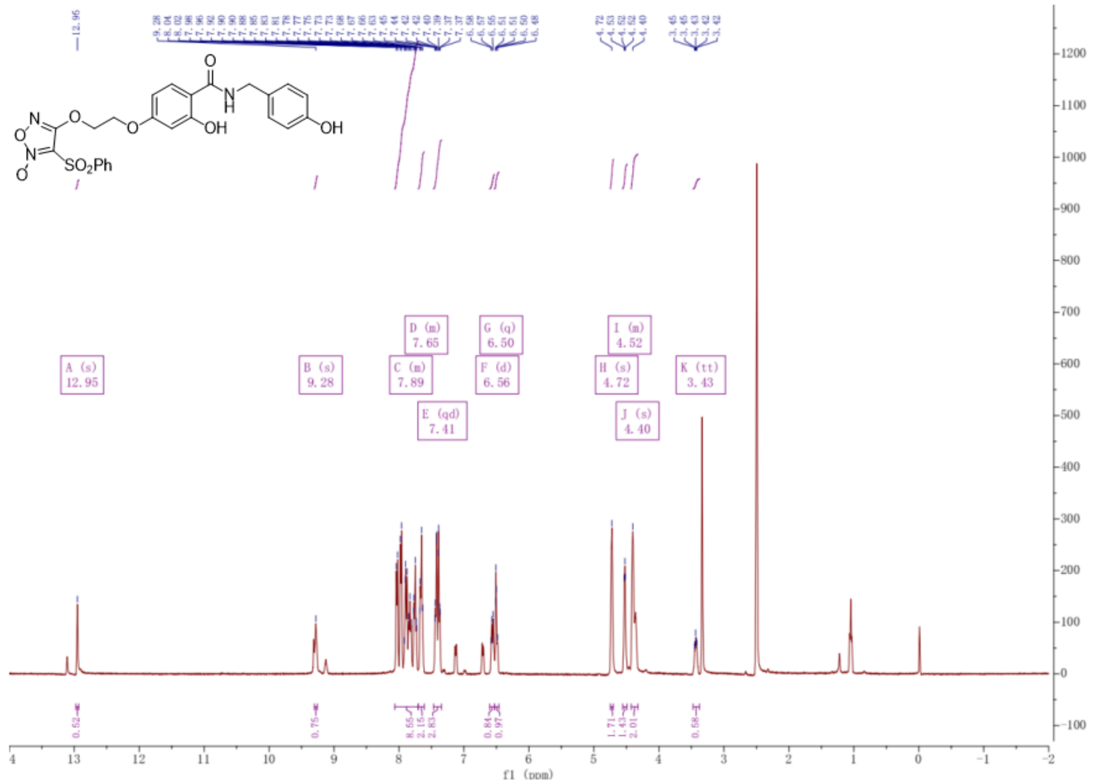

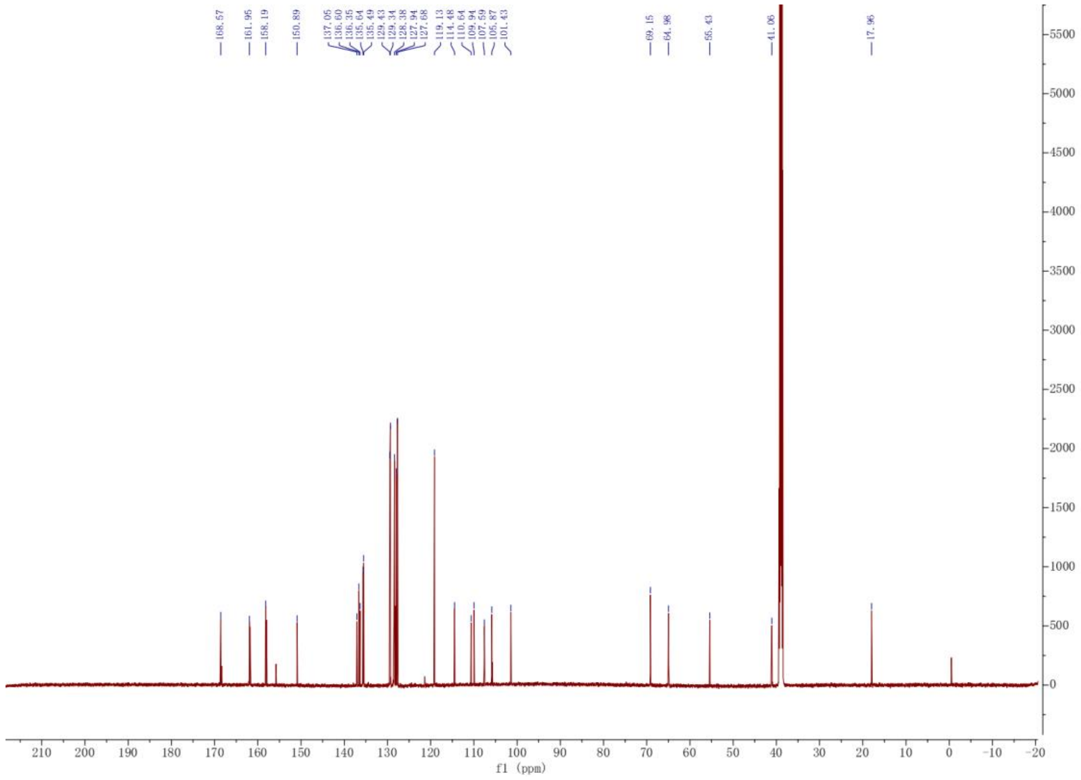

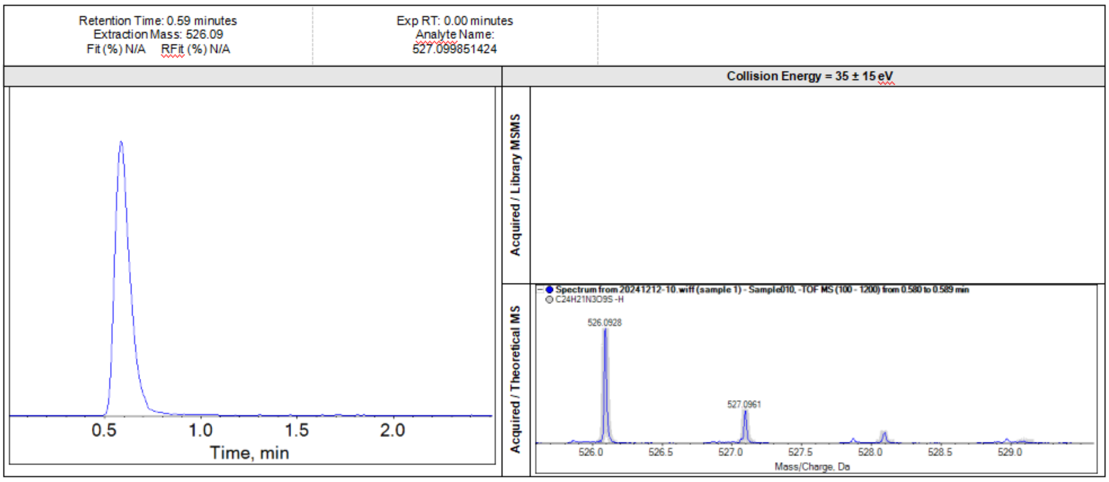

	Compound Name (Library Hit)	Score	Formula	Intensity	Threshold	Expected m/z	Found at m/z	Error (ppm)	Expected RT (min)	Found RT (min)	RT Delta (min)	Isotope Diff (%)	Library Score (%)
	527.099851424 (No data for Library Hit Name xcm)	96%	C24H21N3O9S	13811	5	526.0926	526.0928	0.4	0.00	0.59	0.59	1.3%	N/A

^{1}H NMR, ^{13}C NMR and HRMS Spectra of Compound 18a

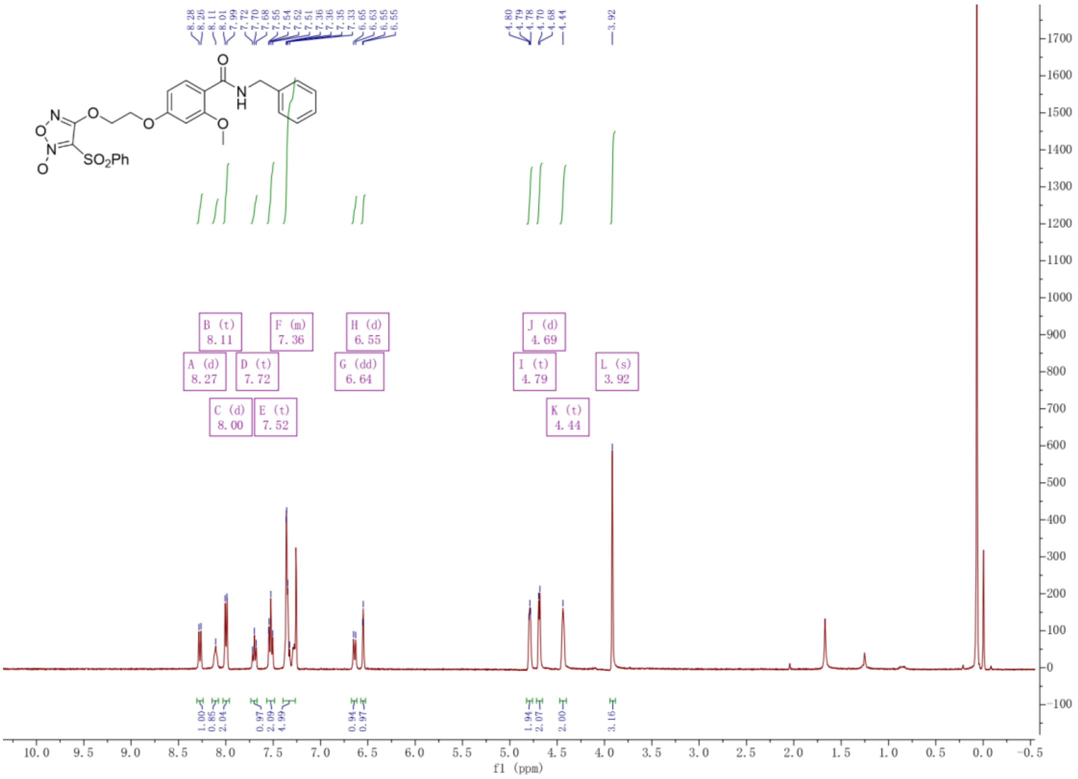

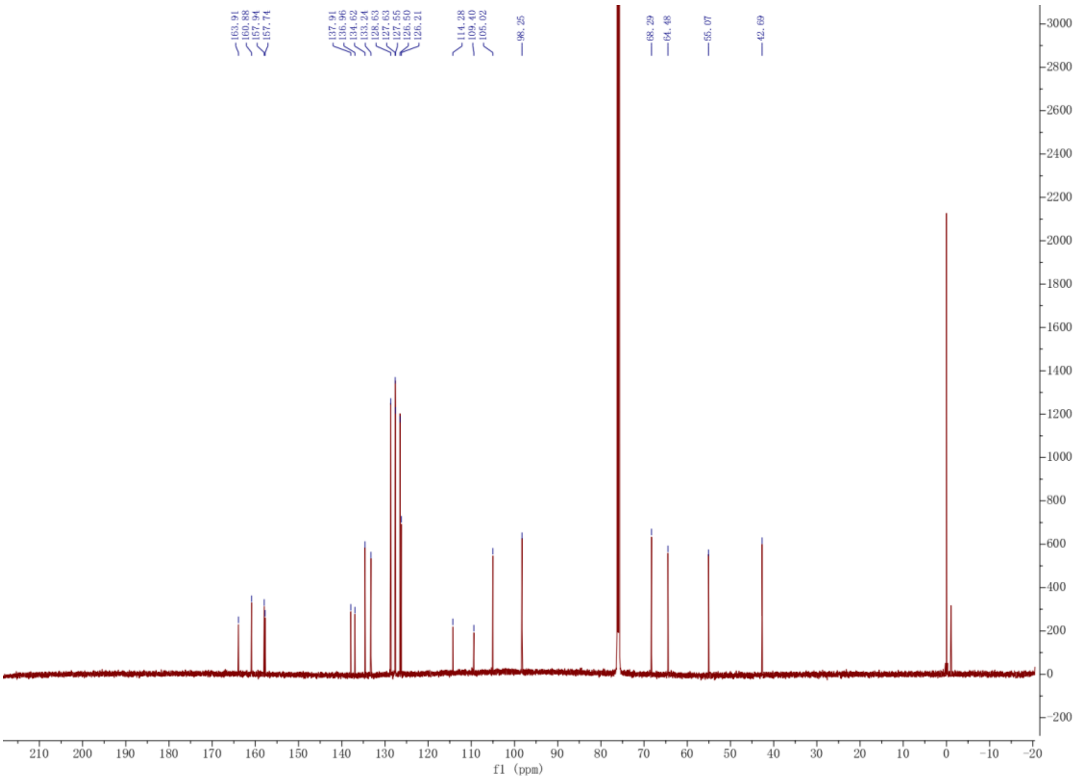

Retention Time: 0.64 minutes
Extraction Mass: 526.13
Fit (%) N/A RFit (%) N/A

Exp RT: 0.00 minutes
Analyte Name:
525.120586864

Collision Energy = 35 ± 15 eV

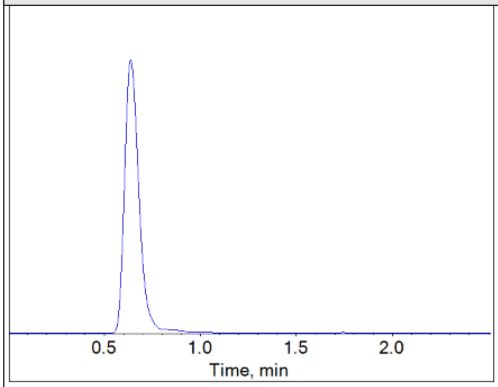

Acquired / Library MSMS

Acquired / Theoretical MS

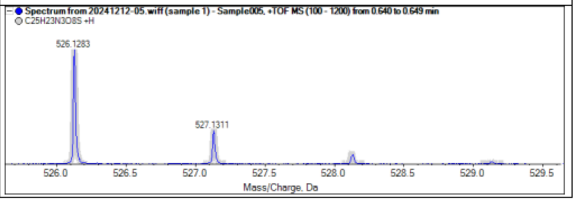

	Compound Name (Library Hit)	Score	Formula	Intensity	Threshold	Expected m/z	Found at m/z	Error (ppm)	Expected RT (min)	Found RT (min)	RT Delta (min)	Isotope Diff (%)	Library Score (%)
	525.120586864 (No data for Library Hit Name.xcm)	93%	C25H23N3O8S	520735	5	526.1279	526.1283	0.8	0.00	0.64	0.64	2.6%	N/A

^{1}H NMR, ^{13}C NMR and HRMS Spectra of Compound 18b

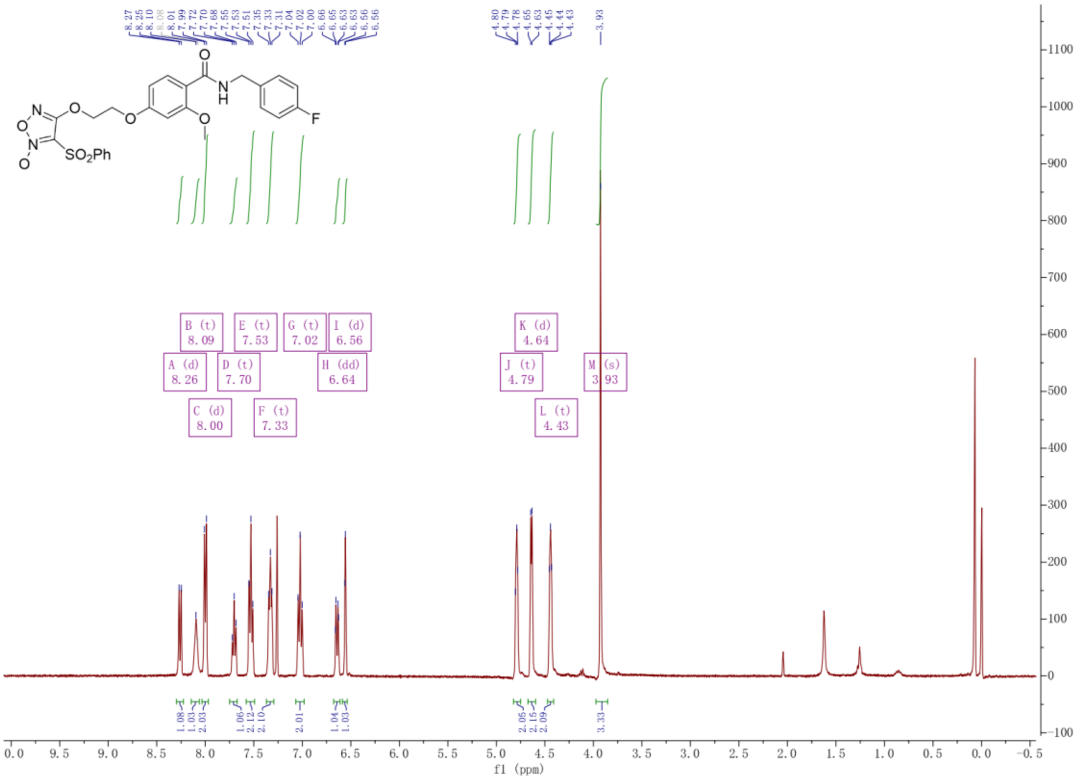

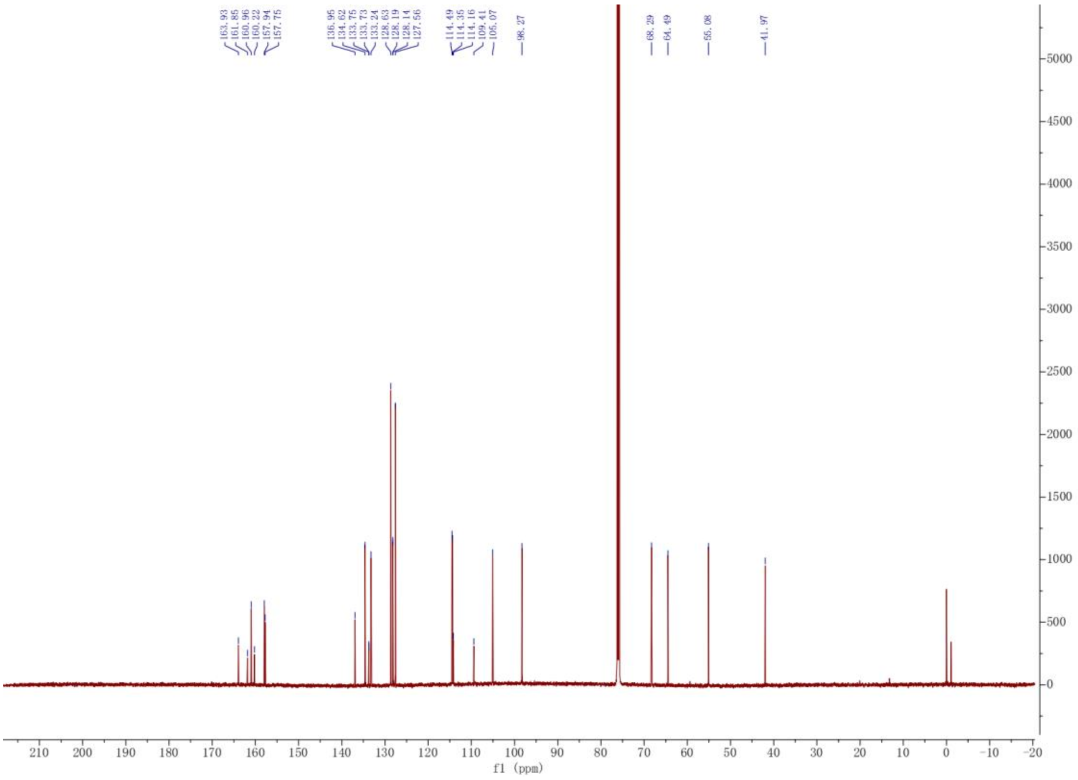

Retention Time: 0.63 minutes
Extraction Mass: 544.12
Fit (%) N/A   RFit (%) N/A

Exp RT: 0.00 minutes
Analyte Name:
543.111165074

Collision Energy = 35 ± 15 eV

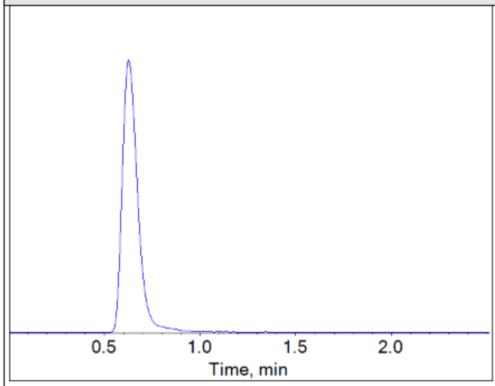

Acquired / Library MSMS

Acquired / Theoretical MS

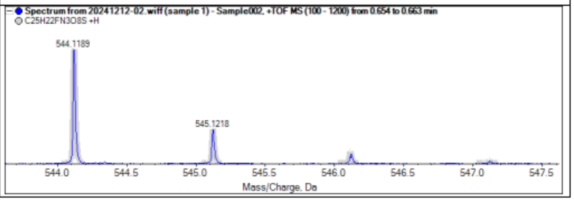

	Compound Name (Library Hit)	Score	Formula	Intensity	Threshold	Expected m/z	Found at m/z	Error (ppm)	Expected RT (min)	Found RT (min)	RT Delta (min)	Isotope Diff (%)	Library Score (%)
✓ ● ✓ ● ●	543.111165074 (No data for Library Hit Name xcm)	93%	C25H22FN3O8S	525927	5	544.1184	544.1189	0.9	0.00	0.63	0.63	2.3%	N/A

^{1}H NMR, ^{13}C NMR and HRMS Spectra of Compound 18d

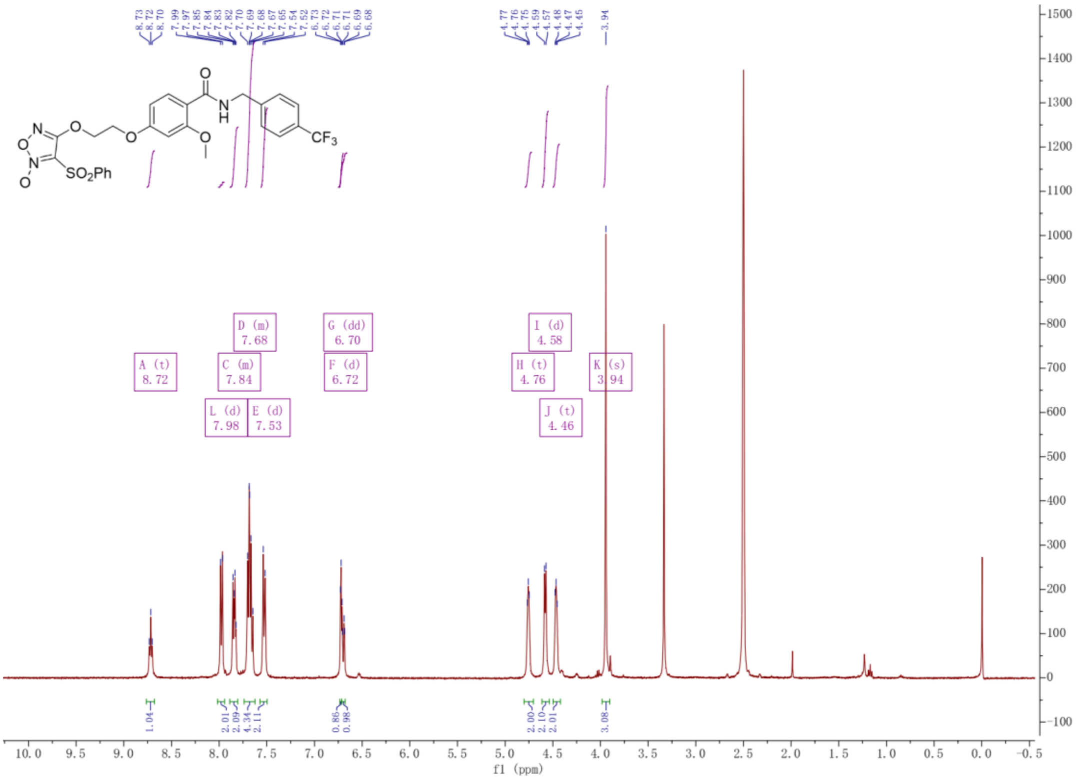

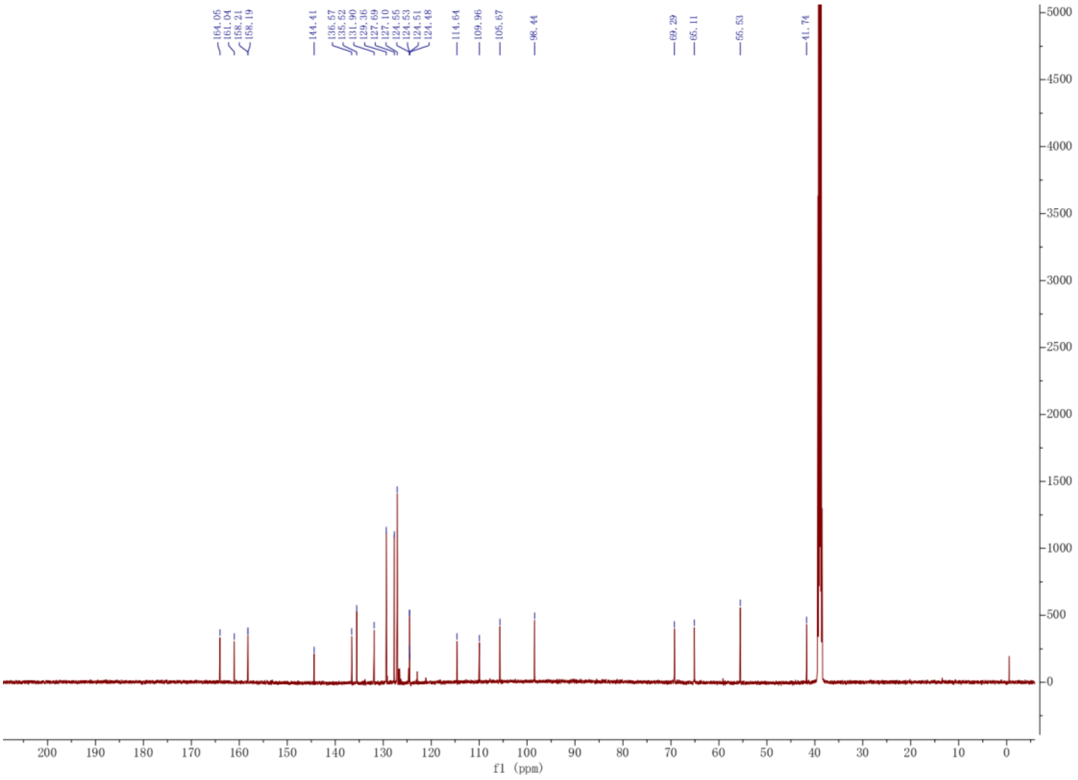

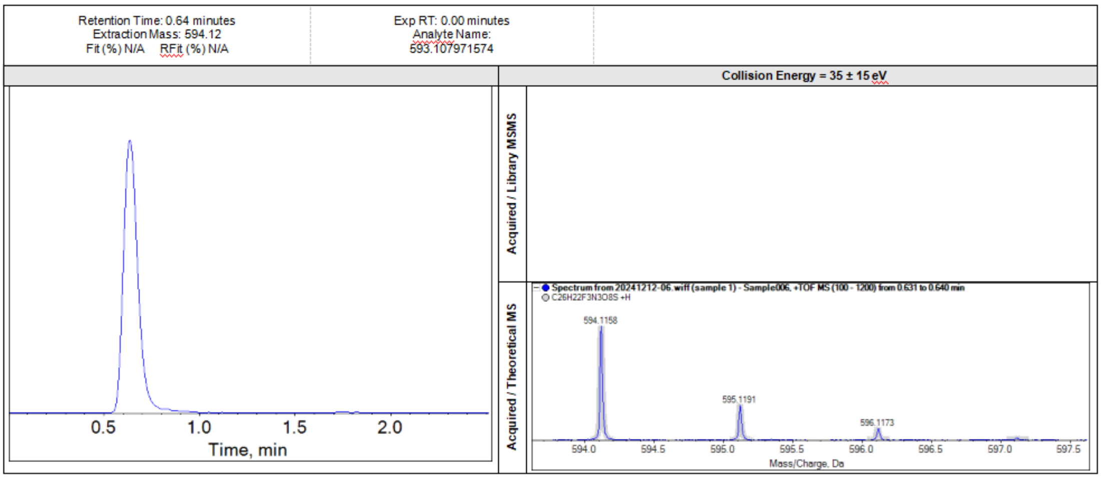

	Compound Name (Library Hit)	Score	Formula	Intensity	Threshold	Expected m/z	Found at m/z	Error (ppm)	Expected RT (min)	Found RT (min)	RT Delta (min)	Isotope Diff (%)	Library Score (%)
✓●✓●●	593.107971574 (No data for Library Hit Name xcm)	93%	C26H22F3N3O8S	218223	5	594.1152	594.1158	0.9	0.00	0.64	0.64	0.8%	N/A

^{1}H NMR, ^{13}C NMR and HRMS Spectra of Compound 18e

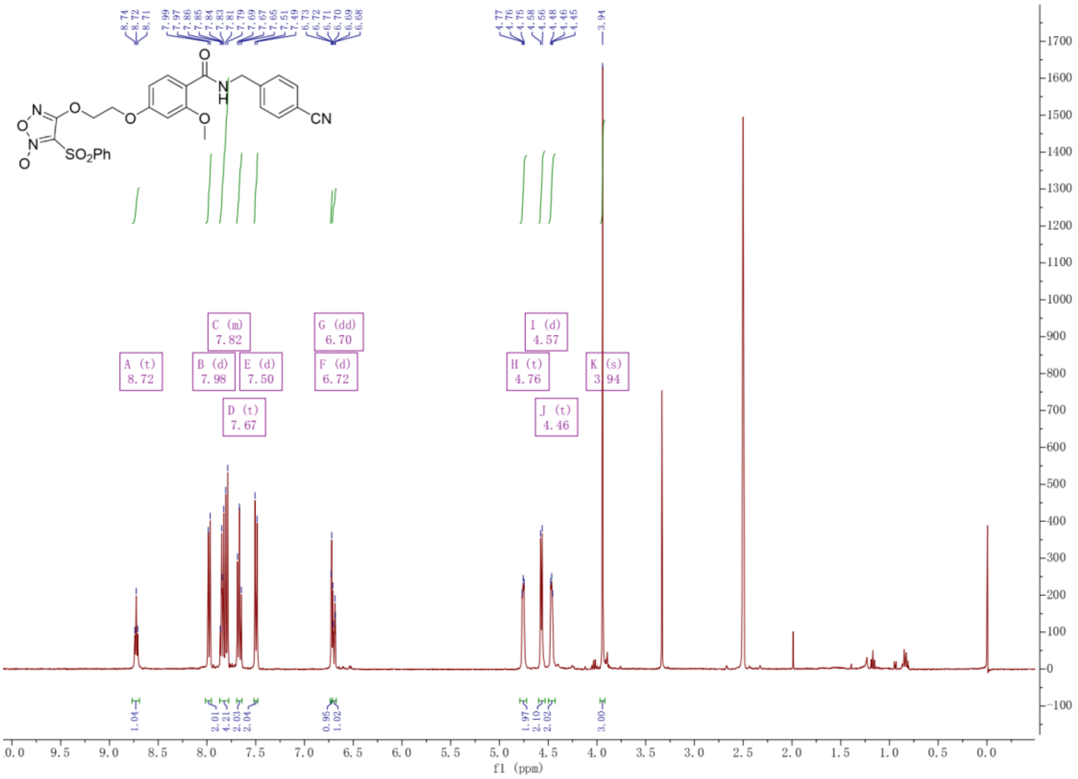

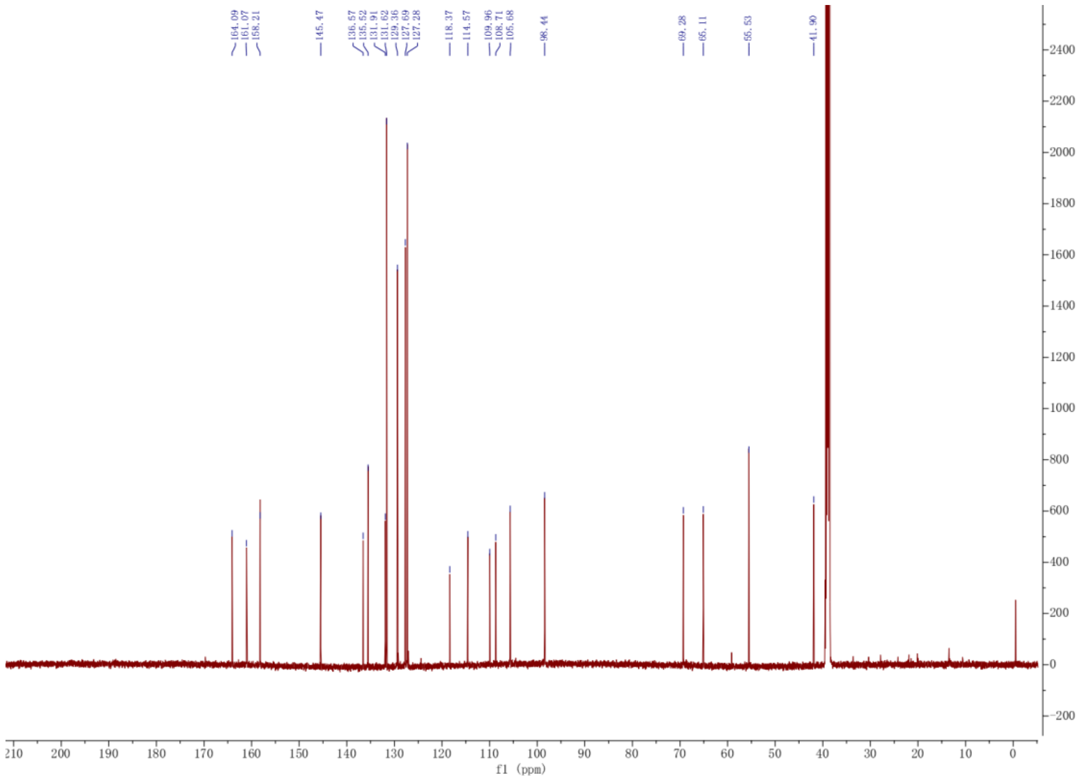

Retention Time: 0.60 minutes<br>Extraction Mass: 551.12<br>Fit (%) N/A RFit (%) N/A	Exp RT: 0.00 minutes<br>Analyte Name:<br>550.115835832

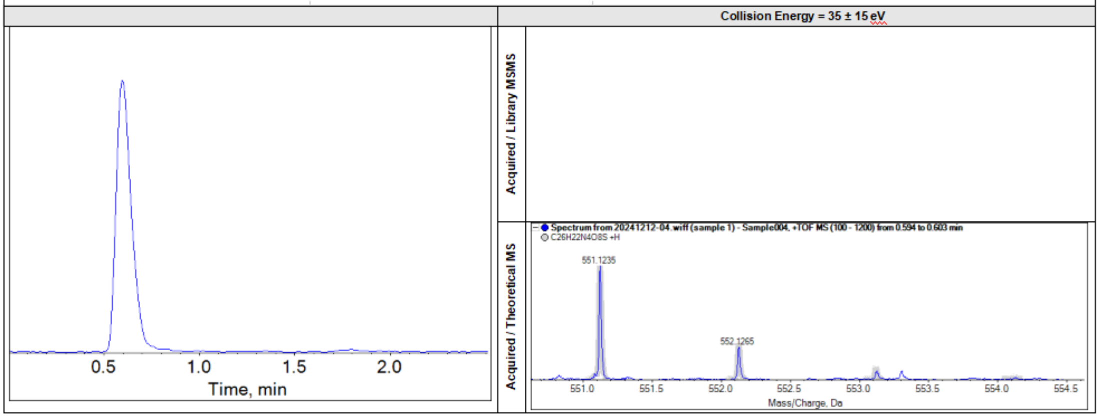

	Compound Name (Library Hit)	Score	Formula	Intensity	Threshold	Expected m/z	Found at m/z	Error (ppm)	Expected RT (min)	Found RT (min)	RT Delta (min)	Isotope Diff (%)	Library Score (%)
✓ ● ✓ ● ●	550.115835832 (No data for Library Hit Name xcm)	93%	C26H22N4O8S	68653	5	551.1231	551.1235	0.8	0.00	0.60	0.60	3.4%	N/A
